# 4D polycarbonates via stereolithography as scaffolds for soft tissue repair

**DOI:** 10.1038/s41467-021-23956-6

**Published:** 2021-07-05

**Authors:** Andrew C. Weems, Maria C. Arno, Wei Yu, Robert T. R. Huckstepp, Andrew P. Dove

**Affiliations:** 1 School of Chemistry, University of Birmingham, Birmingham, UK; 2 School of Life Sciences, University of Warwick, Coventry, UK

**Keywords:** Tissue engineering, Biomedical materials, Mechanical properties

## Abstract

3D printing has emerged as one of the most promising tools to overcome the processing and morphological limitations of traditional tissue engineering scaffold design. However, there is a need for improved minimally invasive, void-filling materials to provide mechanical support, biocompatibility, and surface erosion characteristics to ensure consistent tissue support during the healing process. Herein, soft, elastomeric aliphatic polycarbonate-based materials were designed to undergo photopolymerization into supportive soft tissue engineering scaffolds. The 4D nature of the printed scaffolds is manifested in their shape memory properties, which allows them to fill model soft tissue voids without deforming the surrounding material. In vivo, adipocyte lobules were found to infiltrate the surface-eroding scaffold within 2 months, and neovascularization was observed over the same time. Notably, reduced collagen capsule thickness indicates that these scaffolds are highly promising for adipose tissue engineering and repair.

## Abstract (web summary)

Shape memory scaffolds are needed for minimally invasive tissue repair and void filling. Here the authors report on the development of 4D printed polycarbonate-based scaffolds with surface degradation properties which fill voids without deforming tissue and allow for tissue ingrowth with reduced immune response.

## Introduction

Nature has created tissue designs that are ideally suited for their purpose: bone possesses high strength with enough flexibility to not be brittle; arteries are elastomeric without being flimsy; and adipose tissue is soft and yielding while being durable. Despite these evolutionary materials templates, tissue engineering is currently limited by an urgent, unmet need for improved restorative and healing techniques to address tissue defects. An example of this is in soft tissue repair, such as following breast cancer treatment, a mentally and physically debilitating cancer affecting millions of patients globally^1–4^. Tissue scaffold design has the potential to revolutionize patient care, with supportive materials being essential to resolve wounds that are otherwise untreatable. Porous materials in particular offer tissues the mechanical support that is necessary for them to rapidly infiltrate a space. This concept has been successfully demonstrated in applications such as siloxane maxillofacial implants, polyurethane cardiovascular occlusive devices, and collagen-derived adipose tissue scaffolds^5–11^. However, current porous materials leveraged for these applications are limited by their pore morphological distribution, in addition to other issues such as post-processing requirements prior to use^5–7,9–21^. 3D printing has emerged as one of the most promising methods to overcome these processing limitations as a consequence of the reproducible, interconnected pore features with micron-scale resolution that can be manufactured in precise designs. However, a significant current limitation in the field is the lack of suitable materials that can be processed efficiently by additive manufacturing techniques, particularly in the layer-integrating photopolymerization methods, coupled with long-term biocompatibility in vivo^5,17,22–27^. The primary 3D printing material focus has remained on acrylate- and epoxide-containing polymers, which have low toxicity thresholds, while the primary degradable biomaterial focus has been directed towards poly(l-lactic acid) (PLLA), which is limited by its poor processability in photopolymerizations and its acidic degradation products^25–29^. An alternative synthetic materials platform that can balance minimally invasive behavior with nutrient/waste diffusion and support tissue regrowth as they degrade in vivo to nontoxic byproducts without succumbing to the limitations of current tissue engineering therapies is needed.

Recently, the concept of 4D materials has emerged, where a 3D-printed material displays behavior such as shape memory, swelling, or controlled degradation in a 4th dimension, typically time^5,22,30,31^. Shape-changing polymers open avenues into printing minimally invasive medical devices and scaffolds as well as biomimetic designs, as smaller footprints result in lower surgical trauma without compromising, and in some cases enhancing, the patient outcomes^8,9,22,32–35^. While the current state-of-the-art in minimally invasive biomaterials, such as foams, display the desired reduced therapeutic footprint, their uneven pore distribution restricts nutrient diffusion. Furthermore, improvements in reproducibility of shape-changing behaviors and increased materials choice to improve biocompatibility and biodegradation would represent significant advances^10,11,13,36^. Despite the potential advantages of a 3D-printed, non-inflammatory, resorbable, and shape responsive polymer, there are few examples of 4D printing of minimally invasive, clinically relevant material designs^22,37^.

Herein, we describe an approach to deliver soft tissue engineering constructs by developing 4D printable resin inks that can be photopolymerized into patient-specific, self-fitting scaffolds that can be printed with a wide range of surface morphologies and display tunable shape memory with high strain recoveries and low expansion forces. Furthermore, the materials degrade by a surface-erosion profile to nonacidic products and display excellent cytocompatibility and biocompatibility. By focusing on the design of a material with a unique combination of features, we have been able to achieve a minimally invasive 4D structure that could reduce surgical impact while enhancing rates of healing and patient recovery.

## Results

### Fabrication of resin Inks and photopolymerization printing

**Fig. 1:**
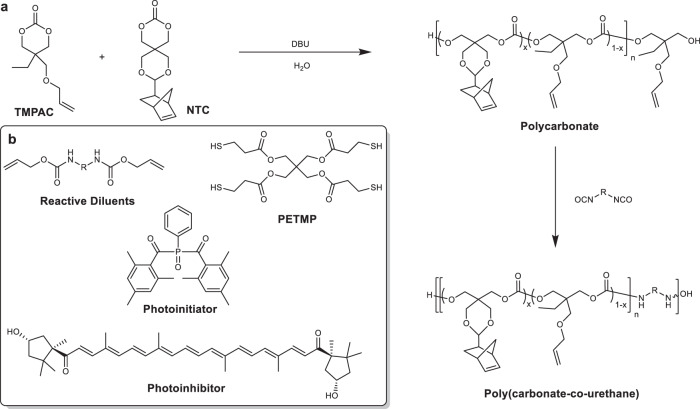
General reaction scheme for producing aliphatic polycarbonate-derived photopolymer resins. Aliphatic polycarbonates were synthesized using organobase-catalyzed ROP of cyclic carbonate monomers, producing low viscosity prepolymer polycarbonates to which were added allyl- and norbornene-functionalized crosslinkers and multifunctional thiols (**a**) and combined with reactive diluents (R = hexamethylene and isophorone), a photoinitiator and photoinhibitor to produce a liquid resin (**b**).

An ideal soft tissue biomaterial would be an elastomeric, compliant, and degradable void-filling 3D structure that can facilitate tissue infiltration. In order to achieve the degradable polymer backbones without acidic degradation while maintaining good control over the synthesis, organocatalytic ring opening polymerization (ROP) of aliphatic cyclic carbonates was selected. This process yielded homo- and co-oligocarbonates from allyl- and norbornene-containing monomers (2-allyloxymethyl-2-ethyltrimethylene carbonate (TMPAC) and 2-norbornene-5,5-bis(hydroxymethyl) trimethylene carbonate (NTC), respectively) with a targeted number-average molar mass (*M*_n_) *ca*. 2 kDa and a dispersity, *Ɖ*_M_, of 1.1 (Fig. 1a). Analysis by ^1^H nuclear magnetic resonance (NMR) and Fourier-transformed infrared (FT-IR) spectroscopy confirmed the presence of carbonyl, hydroxyl, and alkene groups which, in addition to size exclusion chromatography (SEC) analysis, confirm oligomer synthesis (Supplementary Figs. 1–28). Physically, oligomers with higher NTC content resulted in solid polymers, while polyTMPAC homopolymer and those with high TMPAC contents were slightly viscous oils.

**Fig. 2:**
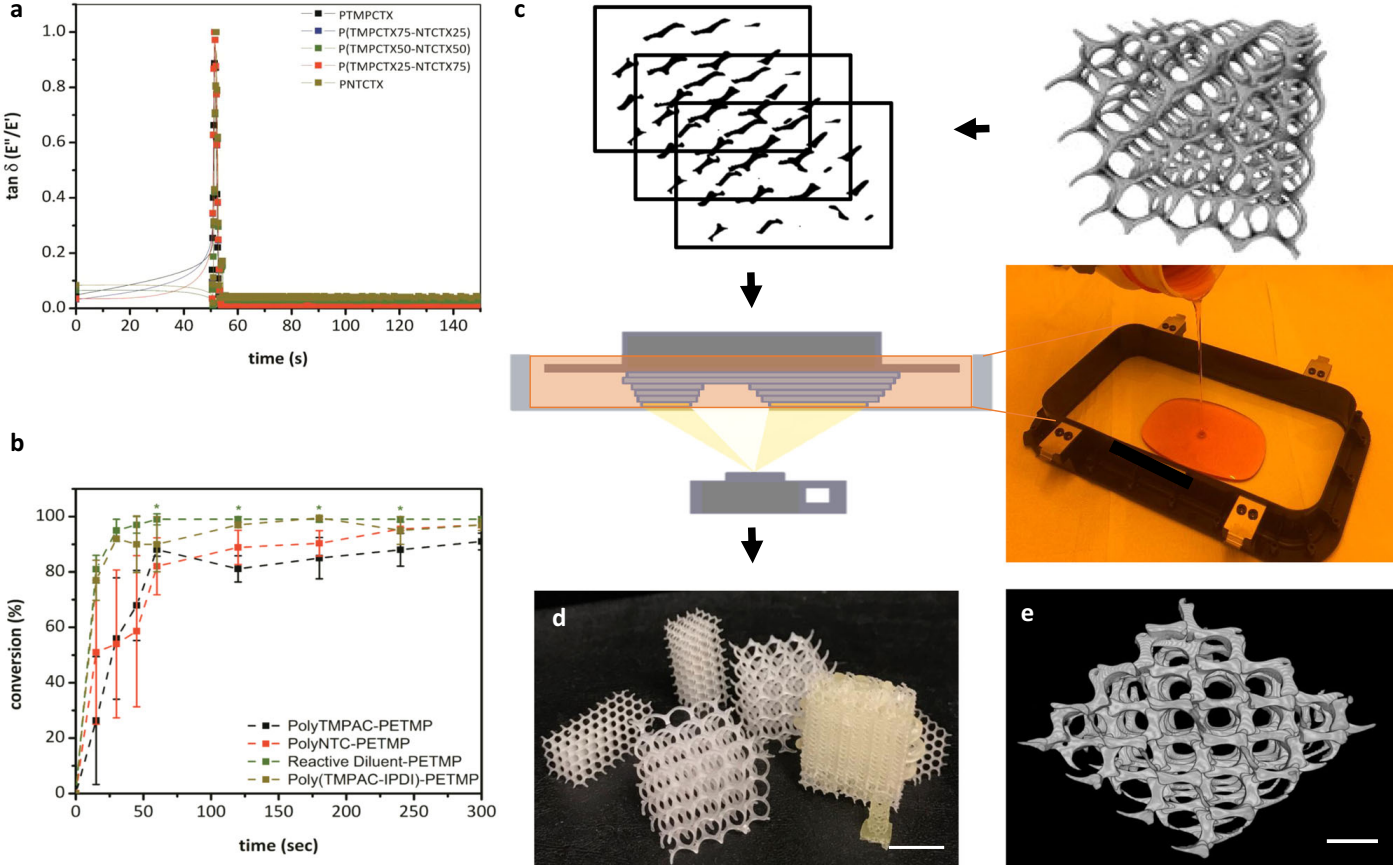
Materials formulation and 3D printing. Gelation times, which correspond with photorheological phase transition behavior studies of resins (**a**) and conversion of alkene monomers to thioether-containing species via thiol-ene radical reactions with PETMP (**b**) irradiated at 340–430 nm at 10 mW·cm^−2^ for 30 s for different resin compositions. Data are presented as mean values with error bars = standard deviation (*n* = 3). Porous scaffolds were reduced to 2D images and photocrosslinked in a digital light processing (DLP) 3D printing process (**c**) and were used to produce the same porous structures as the CAD renderings (pictograph of example prototoypes, scale bar = 2 mm (**d**) and micro-CT representative image, scale bar = 500 µm (**e**).

The miscibility of the oligomers was examined using chain extension with aliphatic diisocyanates to yield poly(carbonate urethane)s (PCUs) (Fig. 1a), or by the addition of urethane-containing reactive diluents that were used as viscosity modifiers that would incorporate into the final material and prevent shrinkage or cracking. Additionally, the oligomers were diluted and solubilized into PETMP (pentaerythritol tetrakis (mercaptopropanoic acid)) (Fig. 1b) to reduce viscosity below 10 Pa·s and create resins suitable for photo-initiated crosslinking and 3D printing^38^ via radical thiol-ene addition (Supplementary Fig. 29)^39,40^. The chain extended PCUs displayed viscosities more than an order of magnitude higher than the polycarbonate resins and would therefore be more difficult to process. For this reason, the focus remained on the urethane-containing reactive diluents for the majority of subsequent testing. A photoinitiator active at *λ* = 405 nm and a paprika extract-derived photoinhibitor with competitive absorbance in the same region (Supplementary Fig. 31) resulted in orange, slightly viscous resin inks (in batches up to 150 g) which when processed, allow a high degree of spatial control without competitive absorbance by the polymeric resin components. The liquid resins rapidly underwent phase transitions to gelled solids upon irradiation in the visible light spectrum (*λ*_max_ = 405 nm). In line with rapid solidification of the resin upon exposure to light, photorheological analysis revealed a peak loss factor ratio at 2 s after irradiation and a dramatic increase in both storage modulus and complex viscosity, from 179.6 ± 17.5 Pa to 1.5 ± 0.4 MPa and 3.1 ± 0.1 Pa·s to 23.1 ± 8.3 MPa·s, respectively, followed by a plateau even upon further irradiation (Fig. 2a, Supplementary Fig. 32). ^1^H NMR spectroscopic analysis of oligomers and model compounds in the presence of the PETMP crosslinker further confirms the rapid, efficient thiol-ene crosslinking within 30 s of exposure resulting in rapid consumption of the allyl and thiol groups and ultimately, gel formation (Fig. 2b). The chemical flexibility of the resin system enabled polyTMPAC to be used to produce PTMPCTX (polyTMPAC-derived thioether crosslinked) scaffolds, while polyNTC was used to produce PNTCTX (polyNTC-derived thioether crosslinked) scaffolds, where an example 50:50 copolymer of the materials would be P(TMPCTX50-NTCTX50).

Using digital light processing (DLP)^27^, a stereolithographic-type process, all compositions could be used to produce porous scaffolds with potential for void-filling devices, without the need for additional processing to remove foam cell membranes or additives typically found in porous biomaterials^11,15^. To demonstrate the ability to print a range of scaffold geometries, scaffolds were printed with pore sizes ranging from 200 to 1500 µm, and surface areas of between 1 and 3 cm^2^. Analysis of the resulting scaffolds by µCT revealed that the measured pore size values match the theoretical pore sizes calculated from the theoretical porous structure renderings within 5% error (Supplementary Fig. 33).

### Cellular response to carbonate-based materials

**Fig. 3:**
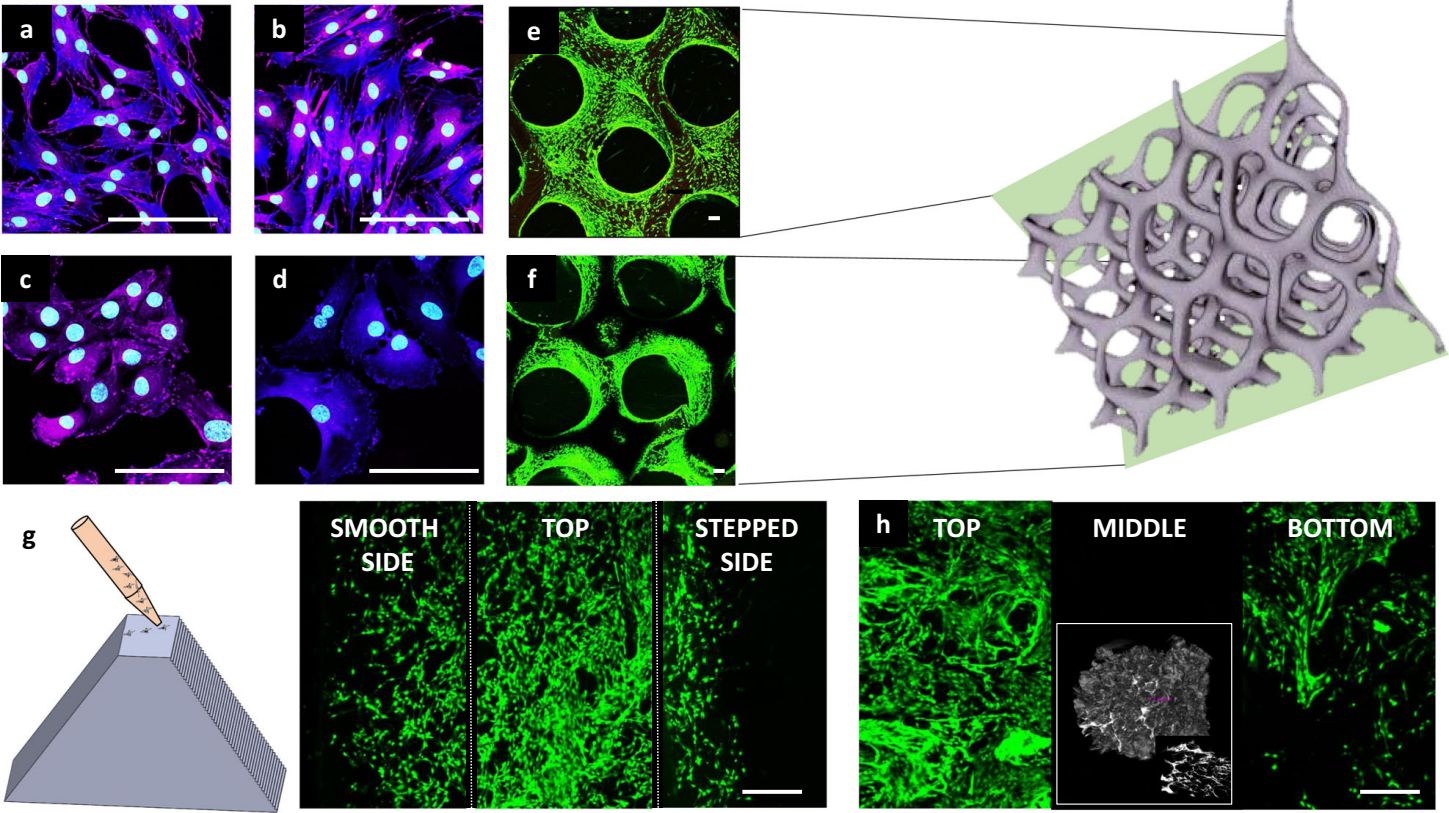
Cellular response to polycarbonate-based materials. Representative images of adipocytes (**a**, **b**) and fibroblasts (**c**, **d**) for PTMPTCX (**a, c**) and PNTCTX scaffolds (**b, d**). Confocal image of adipocytes on 3D PTMPTCX scaffolds at the top (**e**) and bottom (**f**) after 7 days proliferation. (Image **a–d** Scale bar = 10 µm; Image **e, f** Scale bar = 100 µm). Representative printed stair-step pyramidal-structure (with smooth glass-surface cast and alternating stair-step design), with corresponding images from both surfaces overlaid to display cell (pre-osteoblasts) migration after 7 days (**g**), displaying no differences between surface morphology and cellular proliferation. Representative images of cellular proliferation throughout PTMPTCTX foam with images taken at the top of the scaffold (where cells were seeded), bottom of the scaffold, and from the middle of the scaffold after the same time (**h**), inset µCT of foams. Scale bar = 500 µm.

Cytocompatibility screening was performed using 2D surfaces, in order to assess compositional factors prior to final scaffold development, and in 3D scaffolds as a more realistic model. No significant differences were found regarding proliferation or morphology when assessed over a 7-day period (murine fibroblasts, murine adipocytes, murine macrophages, and human fibroblasts) in both direct and indirect contact assays based upon ISO 10993 protocols. All cell types, including macrophages, adipocytes and fibroblasts (murine and human), representative of those found in native adipose tissue, displayed good cell spreading and adhesion (Fig. 3a–d, Supplementary Figs. 34–36). No statistical significance was found for live-dead ratios or proliferation rates over 7 days based on composition for both assay types. In 3D culture, cells were found to proliferate throughout the entire scaffold for pore sizes between 250 and 1500 µm, the essential range for tissue scaffolds to allow for nutrient diffusion and proliferation into a material (Fig. 3e-f; Supplementary Fig. 36)^41^. Attempts to manufacture scaffolds with pore sizes below this threshold were limited by irreproducible pore interconnectivity as a consequence of increased viscosity that prevents resin mobility out of the crosslinked pores and resulted in overcuring.

In order to determine if the step-layer structure that results from the 3D printing process was partly responsible for the high cytocompatibility of the scaffolds, PTMPCTX-based materials were 3D printed into pyramidal structures with a glass-cast smooth side opposite a stair-step side, joined by a flat-top for cell seeding (Fig. 3g). On the pyramidal scaffolds, pre-osteoblasts were found to proliferate equally down both the stair steps as the smooth surface, which indicates that the excellent cytocompatibility is a result of the polycarbonate chemistry rather than surface morphology. To allow further examination of the structural versatility allowed by this approach, materials were also foamed using a modified gas-blowing procedure to produce porous scaffolds (Fig. 3h)^11,15^. The gas-blown scaffolds displayed a high degree of cytocompatibility over 7 days, without the 3D proliferation that was observed in the 3D-printed structures. In the foams, pre-osteoblasts proliferated along the top outer layers of the foam with some also being found along the bottom surface but critically, in contrast the 3D-printed structures (Supplementary Fig. 34), no cellular infiltration into the center of the foams were observed in this time frame (Fig. 3h Middle). This most likely is a consequence of the very fine pore structure and limited interconnectivity between pores limiting diffusion and preventing cellular infiltration beyond the initial layer of pores.

### Scaffold thermomechanical behaviors

**Fig. 4:**
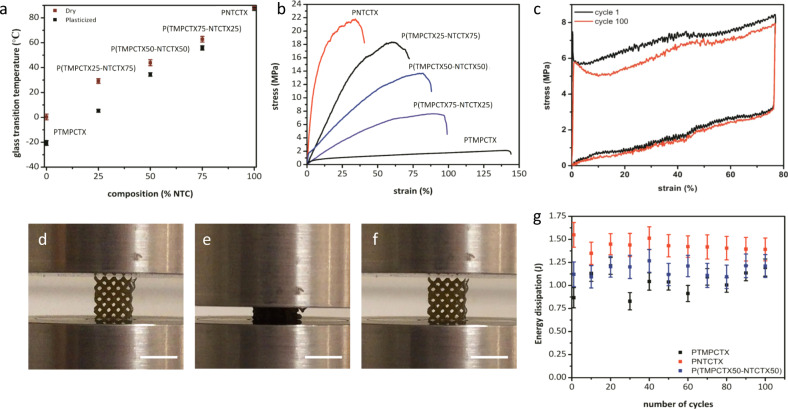
Thermomechanical properties of polycarbonate-based materials. The relationship between *T*_g_ and NTC concentration in the printed polycarbonate materials as determined from phase transitions examined using DMA compression (**a**), stress-strain behavior for dogbones tested at 10 mm·min^−1^ in uniaxial tensile mode (**b**), and representative cyclic compression behavior of printed porous PTMPCTX scaffolds in 37 °C PBS (**c**). Representative images of the PTMPTCX scaffold deformation at 25 °C are shown before loading (**d**), at 70% strain (**e**), and after the load is removed (**f**), with corresponding energy absorption for 100 cycles in alginate gels examined at 37 °C PBS, data are presented as mean values with error bars = standard deviation (*n* = 5) (**g**). Scale bar = 1 cm.

The synthetic versatility of the resin formulation allows the thermomechanical properties of the resultant photocured materials to be tuned with respect to stiffness and stimuli-response temperature (and in turn shape memory response temperature or plasticization in vivo). The carbonate monomer ratio and the presence of urethane linkages were used to tune the glass transition temperature across a range of more than 100 °C in both dry and solvated conditions (Fig. 4a, Table 1, Supplementary Fig. 37). It was found that the same *T*_g_ increase observed with PCUs that are produced through chain extension of polycarbonates could be achieved through incorporation of the isophorone-derived reactive diluent. Higher NTC content in the material increased the dry and plasticized *T*_g_s and also decreased the extent of polymer chain relaxation, as determined thermomechanically through immersion testing in phosphate buffered saline (PBS, pH = 7.4) of cast films examined with dynamic mechanical analysis (DMA). Similarly, the mechanical performance of the materials could also be controlled over a wide range by modulation of the resin composition (Fig. 4b, Table 1). PNTCTX, the highest *T*_g_ composition, displayed a tensile elastic modulus of nearly 660 MPa and ultimate tensile strength of ~22 MPa at 32% strain, after which the material fractured. In contrast, the PTMPCTX material displayed *ca* 140% strain to failure, with an elastic modulus of nearly 15.2 MPa and ultimate strength of 2.1 MPa (Fig. 4b) showing that the materials can be tuned to a potentially broad set of application areas with differing mechanical demands. The materials were all found to be fully elastic until failure at both room temperature and when immersed in PBS at 37 °C (Supplementary Fig. 38).

**Table 1:** Thermomechanical properties of 3D-printed polycarbonates (data are taken from five independent tests on different bars and are presented as mean values with errors = standard deviation).

Composition	Glass transition temperature (*T*_g_) (°C)	Plasticized glass transition temperature (°C)	Compressive modulus (MPa)	Elastic modulus (MPa)	Strain at break (%)	Ultimate tensile strength (MPa)	Toughness (MPa/m^2^)
PTMPCTX	0.3 ± 2.3	−20.6 ± 1.9	1.1 ± 0.5	15.2 ± 7.6	144.1 ± 33.2	2.1 ± 0.1	213.3 ± 51.4
P(TMPTCTX75-NTCTX25)	29.2 ± 1.9	5.3 ± 1.2	3.0 ± 1.2	36.2 ± 5.5	99.0 ± 22.4	7.6 ± 1.4	514.6 ± 97.1
P(TMPTCTX50-NTCTX50)	43.9 ± 2.4	34.5 ± 1.4	12.2 ± 5.0	196.5 ± 12.8	87.8 ± 21.1	13.7 ± 4.9	821.7 ± 157.3
P(TMPTCTX25-NTCTX75)	62.8 ± 2.3	55.8 ± 1.7	9.3 ± 2.0	122.6 ± 18.1	67.2 ± 18.7	18.3 ± 3.8	906.8 ± 192.8
PNTCTX	88.2 ± 1.1	87.1 ± 1.1	12.3 ± 4.8	776.0 ± 59.1	40.6 ± 13.5	22 ± 4.1	723.5 ± 167.9

Generally, all of the polycarbonate scaffolds undergo compression of up to 85% without catastrophic failure (Fig. 4c–g), and above 90% with rearrangement of the macroscale struts into a more compressible orientation, before returning to the original geometry (as a function of the material’s 4D nature). The same mechanical property trends found in tensile testing were repeatable in compressive loading (Supplementary Fig. 38, Supplementary Video 1). Cyclic testing over 100 loading cycles of PTMPCTX in PBS at 37 °C resulted in minimal mechanical behavior change (elastic moduli of 1.1 MPa over the duration of testing; yield stress = 7.4 MPa vs 5.8 MPa, ultimate stress = 8.4 MPa vs 8.0 MPa, respectively, for cycle 1 compared with cycle 100). We postulate that this is a result of the elastic shape memory response, where the shape is fully recovered upon removal of the load, as opposed to the thermally driven shape memory response in which the shape is gradually recovered as the material thermally equilibrates^8,9^. By comparison, the stiffer PNTCTX (compressive elastic moduli of 12.3 MPa) displayed a gradual reduction in recovered strain after each cycle under ambient conditions, decreasing initially by ∼25% before stabilizing by cycle 15 at ~30% of the original strain; increased delays between compression cycles resulted in further recovery of the material when unloaded.

In order to test the mechanical behavior of the 4D scaffolds in a suitable soft tissue-mimicking 3D environment, alginate hydrogels with tuned temporary crosslinks were selected, owing to their comparable mechanical properties to adipose tissue (elastic moduli of ∼60 kPa)^42,43^. Cyclic compressive testing of alginate gels that contain 3D-printed scaffolds, similar to the testing of bare scaffolds, was further used to examine the scaffold migration and risk of soft tissue damage that result from the scaffold’s presence^42^. After a mock surgical opening using an eye-shaped incision, minimal changes in mechanical behavior were recorded for the compression of gels that contained scaffolds (Fig. 4c, Supplementary Fig. 38). This indicated that despite the gel-material mechanical property mismatch that results from their different composition (primarily for PNTCTX scaffolds), the scaffold deforms with the surrounding hydrogel-based tissue model and remains locked in the void as opposed to nonresponsive adipose implants, which may migrate in vivo^43^. This conclusion is further supported by polymer relaxation studies using immersion DMA, where mechanical properties were measured as a function of immersion time in PBS (Supplementary Fig. 39). While the time to the phase transition peak varies with composition, all compositions become fully relaxed at 37 °C in PBS solution. This relaxation behavior is crucial for the designed shape memory response.

### 4D scaffold behavior

**Fig. 5:**
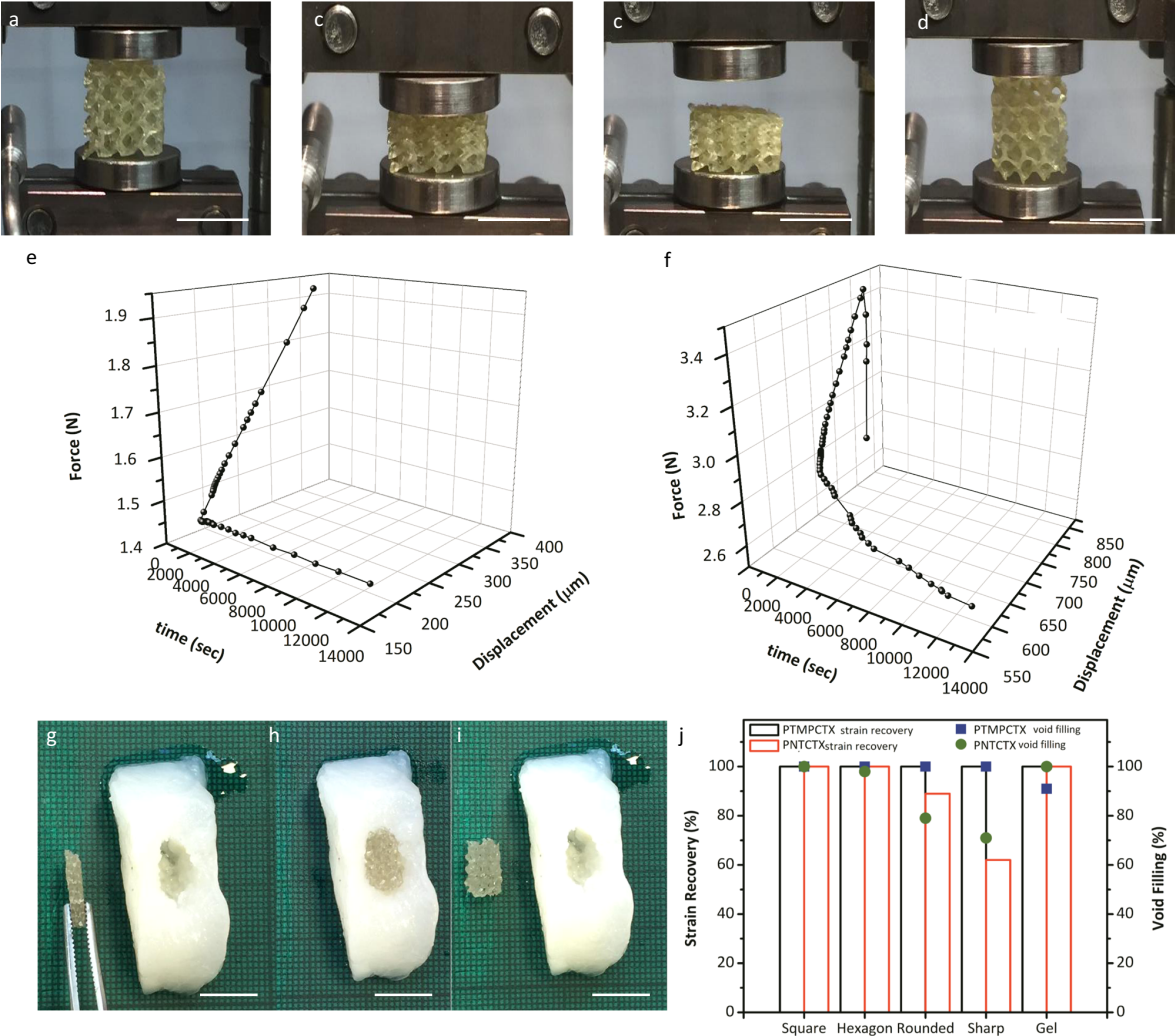
Shape memory behavior. Representative shape memory behavior for a printed porous polyNTC scaffold as it is transitioned from its original geometry (**a**) to a compressed state under loading (~50% strain, **b**), after which it is cooled to 25 °C and will retain its secondary shape after the deformation load is removed (**c**), and the return to the original geometry upon heating of the sample (**d**). The expansion forces of the PTMPTCX (**e**) and PNTCTX (**f**) using compression kinetic studies under in vitro conditions. In vitro void-filling behavior was further examined using compressed scaffolds (represented by PNTCTX here) in soft alginate molds, displaying shape fixation (**g**), void filling without deformation of the alginate (**h**), and shape fixation to the void shape even after removal of the scaffold (**i**); 3D-printed molds were further examined for void-filling efficiency and strain recovery (**j**). (All scale bars = 1 cm).

The carbonate-based materials’ shape memory behavior was quantified by DMA in uniaxial tension, optical measurements (samples were compressed to 80% strain and allowed to recover at ambient conditions and at 37 °C in PBS, Fig. 5a–d) and comparison of expansion behavior in alginate hydrogels, as well as more rigid acrylate-based 3D-printed models, with simplified computational models (Supplementary Fig. 39). The role of NTC content and *T*_g_ (both wet and dry) provided direct correlations with strain recovery behavior for compressed polycarbonate scaffolds (Table 1, Fig. 4a, Supplementary Fig. 37). All of the scaffold compositions displayed shape memory behavior upon immersion at 37 °C in PBS (Supplementary Table 1, Supplementary Fig. 40); less than 25% NTC content in the starting oligomer decreased strain fixation at room temperature, although all compositions displayed 100% strain fixation and recovery when tested at 20 °C below their *T*_g_ (tan *δ* peak) and 20 °C above *T*_g_, respectively. Conversely, increasing NTC content reduced scaffold elasticity and the recovery speed, presumably as a function of scaffold *T*_g_, thereby reducing void filling in irregularly shaped rigid voids (Supplementary Fig. 41). The polycarbonate composition altered the strain fixation and the strain recovery kinetics without impacting the stress recovery, which corresponded well with the thermal behavior. Importantly, this tunability allows for a range of working times when considering potential medical applications; however, specific working times and the selection of an “ideal” formulation will require application-specific performance criteria. Furthermore, this application-specific criterion must also recognize that at sufficiently low or high temperatures utilizing shape memory may not be feasible for all procedures.

Crucial design features of the printed materials are the expansion forces and the relationship with surrounding soft tissue nerves, as the tissue compression that results from scaffold expansion could result in pain, as well as the need to control material deployment in vivo. PTMPTCX scaffolds underwent rapid shape recovery (100% strain recovery) within 45 s, which distorted the alginate by ∼15% (maximum strain) and decreased void-filling efficiency to ~90% as a function of scaffold shape. By comparison, the PNTCTX scaffold displayed slower shape recovery. Passive shape recovery at 37 °C required ∼50 min for full 100% strain and had to be stimulated using H_2_O at 50 °C (active shape memory) to achieve recovery within 10 mins in the alginate void. PNTCTX scaffolds conform to the soft void with 100% void filling and 90% strain recovery (measured at the center of the scaffold), displaying a low expansion force attributed to minimal polymer chain reorientation as a result of their high *T*_g_. Unlike the PTMPTCX scaffolds, which display only decreasing expansion force with immersion (peak expansion force value of 0.52 N ± 0.24 N at 25 °C) and an initial relaxation rate of 1.3 mN·s^−1^ (initial 10 mins), PNTCTX displays an increasing tan *δ* and storage moduli at 37 °C in PBS, followed by a gradual decrease corresponding with the material’s creep response that is indicated by a peak expansion force of 0.71 N ± 0.19 N (at ∼3 mins), and an average relaxation rate of 0.3 mN·s^−1^ (initial 30 mins of immersion). This is ideal behavior because it requires activation of the shape memory response by a surgeon but also enables self-fitting in the soft void. In vivo, this will allow for the void-shape fixing without personalization of the scaffold (i.e., a scaffold capable of fitting itself to a variety of soft voids in a similar manner as injectable hydrogels), and over time the ingrowth of clotting factors and tissue would hold the polymer in the final, void-fitted shape (Fig. 5h–k, Supplementary Video 2). A computational model of a simplistic soft tissue void, using alginate gel mechanical behaviors, revealed maximum deformation similar to what was found experimentally, and indicates the polycarbonates’ ability to undergo typical deformations subjected to native tissue during daily life.

**Fig. 6:**
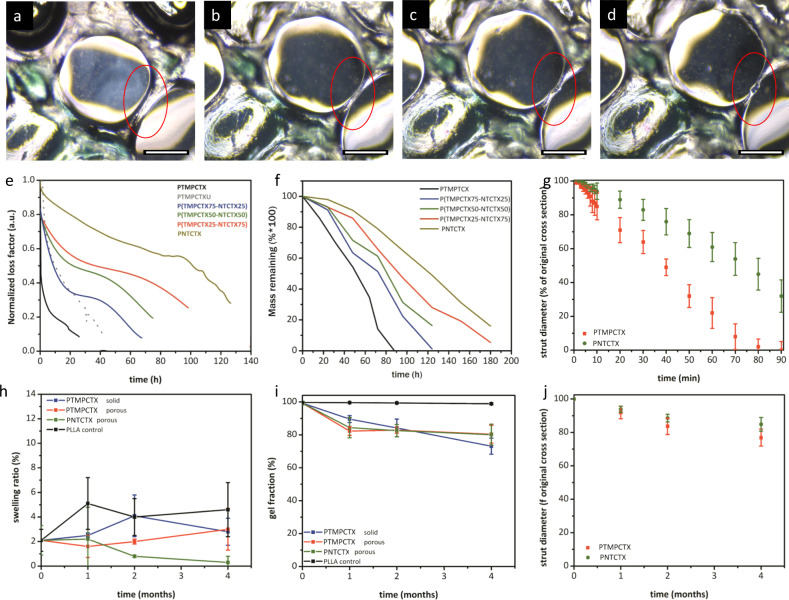
Swelling and degradation behavior of 3D-printed materials. Representative microscopy images of a printed PTMPTCX scaffold immersed in 5 M NaOH over 15 min, demonstrating surface-erosion behavior of the denoted strut (red circle) immediately upon immersion in the solution (**a**), at 5 min (**b**), 10 min (**c**), and 15 min (**d**). Representative curves of uniaxial testing of polycarbonate-derived material films immersed in 5 M hydrolytic degradation solution at 37 °C, with samples deformed 50 µm at 1 Hz until failure (**e**) and corresponding static gravimetric degradation analysis at the same conditions (**f**), along with in vitro strut erosion measurements from microscopic analysis of printed scaffolds immersed in 5 M NaOH at 37 °C (**g**). Post-implantation assessment of sample swelling (**h**) and gel fraction (**i**) were used to assess the extent and type of degradation of the samples after removal from tissue at discrete timepoints, with accompanying measurements of strut diameters from printed scaffolds ex vivo (**j**). Scale bar = 200 µm. Data are presented as mean values with error bars = standard deviation (*n* = 6).

When exposed to hydrolytic degradation conditions, scaffold surface-erosion rates via hydrolysis (Fig. 6a–d) could be predicted by thermal transitions (Fig. 6e-f); the concentration of base also impacted the acceleration of gravimetric change (Supplementary Fig. 42A). Nonporous films were immersed in 5 M NaOH at physiological temperature and were subjected to 10 µm deformation at 1 Hz, resulting in material failure behavior (as defined by film erosion and cracking). While basic conditions can lead to surface erosion^44^, this trend was similar to what is found using static gravimetric analysis with both films and printed, as well as decreased concentrations of NaOH and PBS solution, porous scaffolds, albeit with surface erosion occurring more rapidly as a consequence of the surface deformation caused by mechanical loading as well as the hydrolytic solution to as previously demonstrated in polyesters. All of the materials degraded through a surface-erosion behavior that is demonstrated by the gradual reduction in strut cross-sectional area in printed scaffolds (Fig. 6h, Supplementary Fig. 42, Supplementary Video 3).

In vivo analysis was performed over 4 months in murine subcutaneous implant studies comparing PLLA disks, nonporous PTMPTCX disks, and printed, porous disks of PTMPTCX and PNTCTX with 500 µm pores. Material degradation was evaluated using swelling (Fig. 6i) and gel fraction analysis (Fig. 6j) post-implantation from subcutaneously implanted samples and compared with in vitro behaviors to approximate mass loss over the implantation period as well as surface-erosion rates (Fig. 6k), as well as microscopically for ex vivo porous samples after tissue removal (Supplementary Fig. 42). Material swelling was found to be statistically unchanged over the course of the 4-month study, with PNTCTX displaying the least swelling compared with the other compositions; PTMPTCX swelling ratio is not affected by the porosity or surface area, which indicates that the minimal swelling that does take place is limited by the crosslink density of the residual network. Regarding the intact thermoset network, all compositions displayed greater than 99% gelation prior to implantation. By month 4, all SMPs displayed statistically comparable mass remaining (*ca* 80%), which by extrapolation indicates that total mass loss would most likely occur for the materials within 20 months. Comparatively, the PLLA control materials did not display significant mass loss, which indicates that minimal chain fragmentation is taking place in this same time period. The degradation displayed by the PTMPTCX and PNTCTX would provide sufficient support for more than a year, a seemingly ideal time frame that allows for mature tissue ingrowth before the mechanical support of the scaffolds is sufficiently reduced via degradation^3,45^. Spectroscopic analysis of the implanted samples by FT-IR spectroscopy supports this claim, where minimal shifting of the carbonyl peak indicates less than 30% mass loss has occurred by semi-quantitative analysis of the carbonyl change^11^. This is further supported by ^1^H NMR spectroscopic analysis of extracted samples.

### Host-material response

**Fig. 7:**
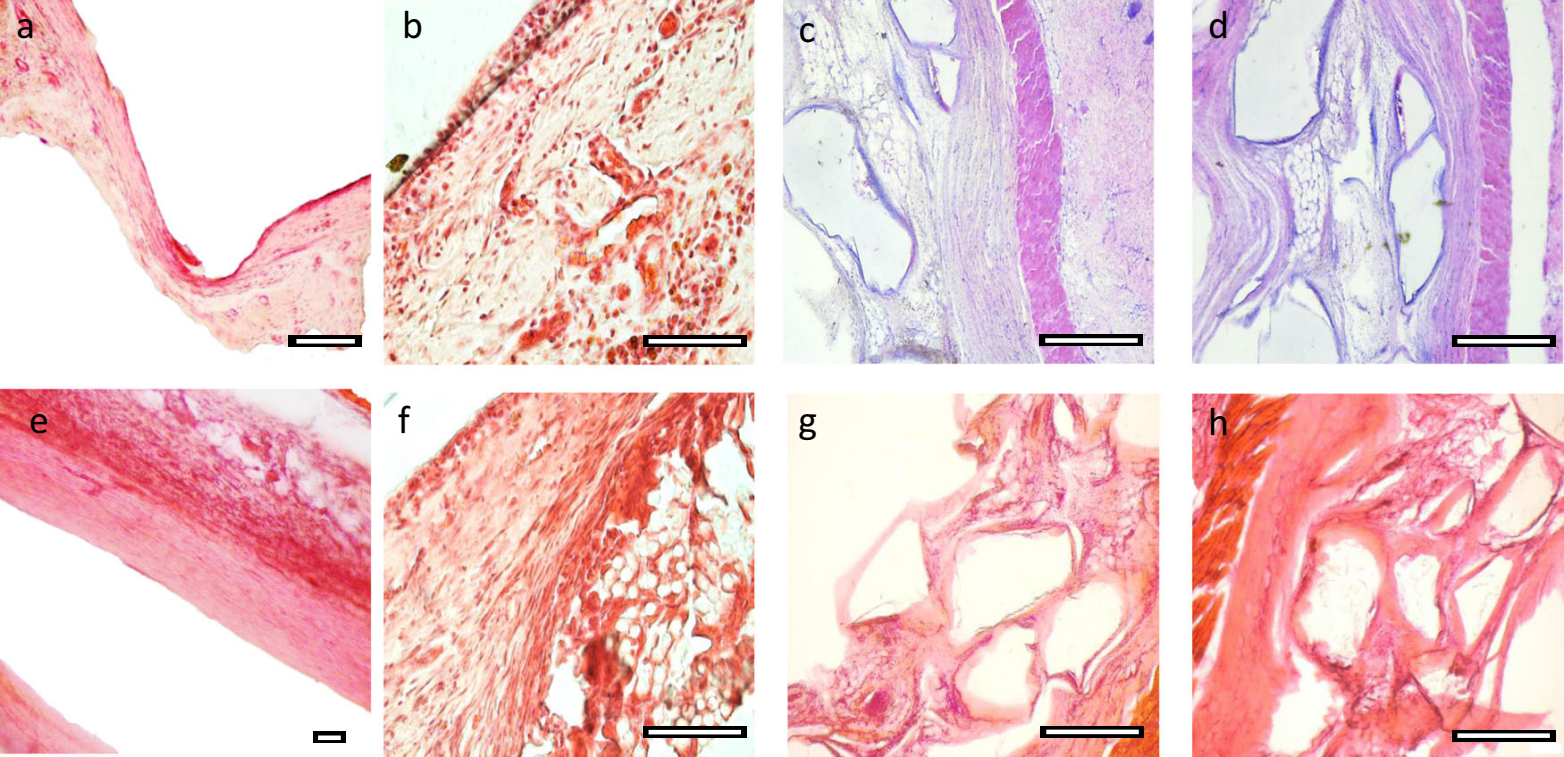
Histopathological analysis. Representative histological images from PLLA control materials at 1 month (**a**) and 4 months (**e**) compared with PTMPTCX films at the same times (**b**, **f**). Masson’s Trichrome (**c**, **d**) and H&E (**g**, **h**) images of PTMPTCX (**c, g**) and PNTCTX (**d**, **h**) printed scaffolds after 4 months, respectively, with corresponding histological scoring and assessment. Scale bar = 200 µm.

Histopathological analysis further indicated the promise of these materials for tissue engineering applications. Haemotoxylin and Eosin (H&E) and Trichrome stains (Fig. 7a–h, Supplementary Figs. 43–50) revealed the presence of adipocyte infiltration by the 1-month timepoint in the porous prints, with minimal lobule formation at this time. However, by 2 months distinct lobules were seen within the pores of the scaffolds as well as on the periphery at the material-tissue original interface which indicates restoration of normal tissue as opposed to damaged or scarred tissue^46–48^. For nonporous polycarbonate-derived material disks, lobules were found within 100 µm of the material surface. Adipocyte shape in vivo further reflects the positive response to the surface, as the characteristic round morphology is found within both the lobules as well as individually^49–51^. Our results indicate that nearly 40% of the infiltrated tissue is represented by adipocyte lobules, with fibroblasts representing another majority of the tissue; PLLA did not display this type of integration over the same time period. Capsule formation around all examined implants was less than 200 µm thick, well below the 500 µm threshold used for biocompatibility in other studies^52^. Importantly, the capsule formation was reduced with increased surface area; the porous implants displayed approximately half the capsule thickness (∼50 µm) as solid polycarbonates, which suggests that there may be a benefit to the 3D structure for tissue engineering implants; PLLA displayed ∼120 µm capsules. Macrophage presence (Table 2) was found to be indicative of healing rather than severe inflammatory response, and the presence of macrophages have been linked to healthy function in adipose tissue, supported by fibroblast presence^53,54^.

**Table 2:** Pathological scoring of the polycarbonate implants (out of 5).

			Adipocytes	Neutrophils	Lymphocytes	Plasma cells	Mononuclear macrophage	Multinucleated foreign giant cells	Platelets	Necrosis	Neovascularization
1 Month	Exterior	Solid PTMPCTX	0.9 ± 0.4	0.0 ± 0.0	0.0 ± 0.0	0.1 ± 0.1	1.7 ± 1.2	0.0 ± 0.0	—	0.0 ± 0.0	—
		Porous PTMPCTX	1.7 ± 0.9	0.0 ± 0.0	0.0 ± 0.0	0.0 ± 0.0	2.6 ± 0.7	0.0 ± 0.0	—	0.0 ± 0.0	—
		Porous PNTCX	2.2 ± 0.7	0.0 ± 0.1	0.0 ± 0.0	0.0 ± 0.0	2.2 ± 0.7	0.0 ± 0.0	—	0.0 ± 0.0	—
	Interior	Solid PTMPCTX	—	—	—	—	—	—	—	—	—
		Porous PTMPCTX	2.1 ± 0.6	0.0 ± 0.0	0.0 ± 0.0	0.0 ± 0.0	2.6 ± 0.9	0.0 ± 0.0	2.1 ± 1.1	0.0 ± 0.0	0.3 ± 0.5
		Porous PNTCX	3.0 ± 1.2	0.0 ± 0.0	0.0 ± 0.0	0.0 ± 0.0	2.1 ± 0.8	0.0 ± 0.0	2.7 ± 1.2	0.0 ± 0.0	0.3 ± 0.4
2 Month	Exterior	Solid PTMPCTX	1.5 ± 0.8	0.0 ± 0.0	0.0 ± 0.0	0.1 ± 0.2	1.4 ± 0.9	0.0 ± 0.0	—	0.0 ± 0.0	—
		Porous PTMPCTX	3.0 ± 1.1	0.0 ± 0.0	0.0 ± 0.0	0.0 ± 0.0	2.2 ± 1.0	0.0 ± 0.0	—	0.0 ± 0.0	—
		Porous PNTCX	3.2 ± 1.3	0.0 ± 0.1	0.0 ± 0.0	0.0 ± 0.0	3.1 ± 1.3	0.0 ± 0.0	—	0.0 ± 0.0	—
	Interior	Solid PTMPCTX	—	—	—	—	—	—	—	—	—
		Porous PTMPCTX	3.0 ± 1.1	0.0 ± 0.0	0.0 ± 0.0	0.0 ± 0.0	2.5 ± 1.1	0.0 ± 0.0	2.8 ± 1.3	0.0 ± 0.0	1.1 ± 0.6
		Porous PNTCX	3.2 ± 1.3	0.0 ± 0.0	0.0 ± 0.0	0.0 ± 0.0	3.1 ± 1.3	0.0 ± 0.0	2.6 ± 1.1	0.0 ± 0.0	0.7 ± 0.6
4 Month	Exterior	Solid PTMPCTX	2.1 ± 0.9	0.0 ± 0.1	0.0 ± 0.0	0.1 ± 0.3	2.1 ± 1.2	0.0 ± 0.0	—	0.0 ± 0.0	—
		Porous PTMPCTX	3.6 ± 0.8	0.0 ± 0.2	0.0 ± 0.0	0.0 ± 0.0	3.1 ± 1.5	0.0 ± 0.0	—	0.0 ± 0.0	—
		Porous PNTCX	3.3 ± 0.9	0.0 ± 0.3	0.0 ± 0.0	0.0 ± 0.0	3.0 ± 1.6	0.0 ± 0.0	—	0.0 ± 0.0	—
	Interior	Solid PTMPCTX	—	—	—	—	—	—	—	—	—
		Porous PTMPCTX	3.2 ± 0.9	0.0 ± 0.0	0.0 ± 0.0	0.0 ± 0.0	2.9 ± 1.4	0.0 ± 0.0	1.1 ± 0.8	0.0 ± 0.0	2.1 ± 1.1
		Porous PNTCX	3.3 ± 0.7	0.0 ± 0.0	0.0 ± 0.0	0.0 ± 0.0	3.1 ± 1.1	0.0 ± 0.0	1.0 ± 0.9	0.0 ± 0.0	2.4 ± 1.2

Vascular bud formation and vascularization occurred by 2 months, with several small, mature vessels found at 4 months in the surrounding tissue but no additional budding. Vascular budding allows for healing to occur, and then ideally will be reduced to match the original tissue as seen here, as adipose tissue is typically not heavily vascularized^1,55^. One of the main failures in contemporary clinical techniques for restoring soft tissue, such as in adipose repair using autologous fat transplantation, is the 40–60% loss of graft volume as a result of poor graft vascularization post-implantation, and aspirated adipocytes are easily damaged by the mechanical force of the procedure, ultimately leading to cyst and localized necrosis that causes immune response and loss of the graft. Neither calcification was found in the implants, nor was necrosis.

## Discussion

Tissue engineering is necessary for many soft tissue deformities, including damage to the head, neck, torso and limbs, and breast cancer treatment^1–3,56,57^. Of particular concern are traumas where tissue is removed, leaving a void that if left unfilled will result in deformity and cosmetic changes. 3D-printed synthetic scaffolds offer a unique opportunity to resolve tissue voids without the limitations of biological materials. Many synthetic materials suffer from degrading to acidic byproducts, cannot be processed well, and do not possess sufficient elasticity and strain recovery to fit to irregular voids. Despite these limitations PLLA and other polyesters are frequently used in tissue engineering applications^3^. However, our carbonate-based, shape memory, 4D scaffolds are nonacidic upon chain scission and can be 3D printed into highly porous scaffolds that provide structural support on a mechanically suitable substrate where surface chemistry may be modified. This allows production of self-fitting scaffolds, which will take on soft tissue void geometry in a minimally invasive surgery without deforming or applying pressure to the surrounding tissue. Over implantation time, our data indicates that the scaffold will hydrolyze and erode away, primarily through a surface-erosion mechanism, with minimal swelling, allowing for a slow, continuous tissue infiltration without acidosis and mechanical failure that is characteristic of long-term PLLA degradation^58^. Unlike previous work which requires aspiration or other biological factors, such as bone morphogenic protein-2, the advantage of our 4D printed scaffolds is the relative simplicity of the system, where the printed material only acts to support the ingrowth of native tissues rather than introducing cellular species, which may increase the host’s immune response^46–48^. This type of behavior is ideal, as the material will provide mechanical support in the void space until sufficient time has passed (and tissue remodeling cycles have taken place).

Ultimately, the unique combination of printability, shape memory, surface-erosion, and excellent biocompatibility, in concert with the ability to tune the thermomechanical properties of the resultant polycarbonates enables them to demonstrate significant potential for application in tissue engineering for various applications. This rationally designed materials allow for synthetic polymeric tissue scaffolds to be easily synthesized and efficiently processed using photopolymerization additive manufacturing. This opens a wide variety of applications in healthcare, manufacturing, and therapeutics geared towards improving patient quality of life. The versatility of these polycarbonate-based systems could enable their applications to be extended to laparoscopic device design or tuned for use in applications such as catheters as well as for use in other minimally invasive surgical techniques for cardiovascular, nephrological, or hepatological surgeries where injectable or compressible stents and patches are required.

## Methods

### Instrumentation

All starting reagents were commercially available, purchased from Sigma–Aldrich (unless otherwise stated), and used without purification. Solvents were of ACS grade or higher. NMR spectra (400 MHz for ^1^H and 125 MHz for ^13^C) were recorded on a Bruker 400 spectrometer and processed using MestReNova v9.0.1 (Mestrelab Research, S.L., Santiago de Compostela, Spain). Chemical shifts were referenced to residual solvent peaks at *δ* = 7.26 ppm (^1^H) and *δ* = 77.16 ppm (^13^C) for CDCl_3_ and *δ* = 2.50 for (^1^H) and *δ* = 39.52 ppm (^13^C) for *d*_*6*_-DMSO. Size exclusion chromatography (SEC) was performed using an Agilent 1260 Infinity II Multi-Detector GPC/SEC System equipped with both RI and ultraviolet (UV) detectors (*λ* = 309 nm); PLGel 3 μm (50 × 7.5 mm) guard column and two PLGel 5 μm (300 × 7.5 mm) mixed-C columns with CHCl_3_ with 5 mM triethylamine as the eluent (flow rate 1 mL/min, 50 °C) were used for separation. A 12-point calibration was developed using poly(methyl methacrylate) standards (PMMA, Easivial PM, Agilent) and applied for determination of molecular weights and dispersity (*Ɖ*_M_). An Anton Paar rheometer (Anton Paar USA Inc, Ashland, VA, USA) fitted with a detachable photoillumination system with two parallel plates (10 mm disposable aluminum hollow shaft plate, Anton Paar) was used for rheology studies using RheoCompass software (v1.20.496). Uniaxial tensile testing was performed using a Testometric MCT-350 equipped with a 100 kgf load cell (Testometric Company Ltd, Rochdale, United Kingdom) and manual tension clamps. Dynamic mechanical analysis was performed using a Mettler-Toledo TT-DMA system (Mettler-Toledo AG, Schwerzenbach, Switzerland) fitted with an immersed static water bath with external recirculating heater system, and samples analyzed using Mettler-Toledo STARe v.10.00 software. 3D printing scaffolds and templates were processed using Solidworks 2019 (Dassault Systemes, Vélizy-Villacoublay, France) and printed using a custom digital light processing system that has been previously reported^59^. Micro-computed tomography analysis was performed using a Skyscan 1172 Micro-CT (e2v technologies plc, Chelmsford, UK) using CT-analyser software V1.15.4.0 (CTAn) (Bruker Micro-CT, Belgium) at an isotropic pixel size of 7-13 μm, a camera exposure time of 500 ms, a rotation step of 0.4°, frame averaging of 5 and medium filtering with a flat field correction. Image reconstruction was performed using a NRecon 1.6.2 (SkyScan, e2v technologies plc, Chelmsford, UK).

### Synthesis of TMPAC monomer^60^

Trimethylolpropane allyl ether (100.0 g, 573.7 mmol) was added to a round bottom flask with 200 mL tetrahydrofuran (THF), and cooled to 0 °C for 1 h. Ethyl chloroformate (124.5 g, 1.1 mol) was added as a single volume to the solution and allowed to again cool to 0 °C for 15 min. Triethylamine (116.2 g, 1.1 mol) was added dropwise over the course of 1 h, at which time the solution was allowed to slowly return to ambient temperature over a 12 h period. The precipitate was filtered off and the solute concentrated to a slightly yellow oil and dissolved in ethyl acetate. The organic layer was washed twice with 1 M HCl and once with brine, and concentrated to a colorless, slightly viscous oil. The oil was distilled to achieve TMPAC (98.8 g, 493.8 mmol, 86% yield). Characterization matched previously reported materials^60^. ^1^H NMR (CDCl_3_, 400 MHz): *δ* = 0.94 (t, ^3^*J*_H–H_ = 7.6 Hz), 1.55 (q, 2 H, ^3^*J*_H–H_ = 7.6 Hz), 3.47 (s, 2 H), 3.88–4.05 (m, 2 H), 4.23 (d, ^3^*J*_H–H_ = 10.1 Hz, 2 H), 4.52 (d, ^3^*J*_H–H_ = 10.1 Hz, 2 H), 5.21–5.42 (m, 2 H), 5.78–5.90 (m, 1 H) ppm. ^13^C NMR (CDCl_3_, 125 MHz): *δ* = 148.59 (C = O), 134.02 (CH), 117.49 (CH_2_), 72.81 (CH_2_), 72.42 (CH_2_), 68.24 (CH_2_), 35.45 (C), 23.31 (CH_2_), 7.37 (CH_3_).

### Synthesis of NTC monomer^61^

Pentaerythritol (40.9 g, 300.6 mmol) was added to a round bottom flask containing deionized water (500 mL), and was subsequently heated to 80 °C with stirring. Once the solids had dissolved, the colorless mixture was then cooled to 20 °C, at which time concentrated HCl (~500 µL, ~2 drops) was added, followed by 5-norbornene-2-carboxaldehyde (30.5 g, 253.8 mmol). The mixture was then stirred for 8 h, and the resulting orange precipitate was isolated using vacuum filtration before being recrystallized from hot toluene/isopropyl alcohol (80/20) as white crystals to yield 2-norbornene-5,5-bis(hydroxymethyl)-1,3-dioxane (NHD). NHD (17.0 g, 71.0 mmol) was dissolved in THF (400 mL) in a round bottom flask and cooled to 0 °C, at which point ethyl chloroformate (20.4 mL, 212 mmol) was added as a single volume, followed by dropwise addition of triethylamine (29.5 mL, 212 mmol) 1 h while maintaining a 0 °C temperature. Upon completing the addition, the reaction was allowed to come to 20 °C and was allowed to stir for 12 h, after which the precipitate was filtered and concentrated to yield white crystals. The white crystals were recrystallized in hot cyclohexane/THF (90/10) (15.4 g, 58.7 mmol, 71%) as the NTC monomer. Characterization matched previously reported materials^61,62^. ^1^H NMR (DMSO-*d*_*6*_, 400 MHz): *δ* = 6.17 (m, 1 H, ^3^*J*_H–H_ = 5.7, 3.0 Hz), 5.93 (m, 1H, ^3^*J*_H–H_ = 5.7, 2.8 Hz), 4.51 (s, 2 H), 4.06 (s, 2 H), 3.89–3.83 (m, 3 H), 3.61–3.58 (m, 2 H), 2.85 (s, 1 H), 2.78 (s, 1 H), 2.22 (ddd, 1 H, ^3^*J*_H–H_ = 12.8, 8.6, 3.9 Hz), 1.75 (m, 1H, ^3^*J*_H–H_ = 12.8, 9.3, 3.8 Hz), 1.31–1.17 (m, 2 H), 0.74 (m, 1 H, ^3^*J*_H–H_ = 11.9, 4.1, 2.6 Hz). ^13^C NMR (DMSO-*d*_*6*_, 125 MHz): *δ* = 148.52 (C = O), 138.00 (CH), 132.48 (CH), 107.38 (CH), 71.49 (CH_2_), 70.37 (CH_2_), 68.91 (CH_2_), 49.28 (CH_2_), 43.57 (CH), 42.20 (CH), 31.53 (CH), 28.34 (CH_2_).

### Synthesis of aliphatic polycarbonate

Ring opening polymerization of the cyclic monomers was used to obtain oligomers. To an open round bottom flask, CHCl_3_ and cyclic monomer(s) were added followed by 1,8-diazabicyclo[5.4.0]undec-7-ene (DBU). For PolyTMPAC, TMPAC (100 g, 500.0 mmol) was dissolved in 100 mL CHCl_3_. DBU (1.44 g, 9.5 mmol) and water (150 µL, 8.3 mmol) were added as a single unit. The resulting solution was stirred for 24 h at 20 °C, after which the DBU was quenched with the addition of Amberlyst A15 H^+^ acidic resin, precipitated into ice cold hexanes, and was then filtered through a silica plug in ethyl acetate. The solution was concentrated in vacuo to yield a viscous, colorless liquid (96.2 g, 96%).

#### 100% TMPAC

^1^H NMR (DMSO-*d*_*6*_, 400 MHz,): *δ* = 0.82 (t, ^3^*J*_H–H_ = 7.6 Hz, 3H), 1.45 (d, ^3^*J*_H–H_ = 9.4 Hz, 2H), 3.32 (s, 2H), 3.87 (dd, ^3^*J*_H–H_ = 5.4 Hz ^3^*J*_H–H_ = 1.8 Hz, 2H), 4.04–4.21 (m, 4H), 5.11–5.32 (m, 2H), 5.79–5.93 (m, 1H), 6.88 (s, 1H). ^13^C NMR (DMSO-*d*_*6*_, 125 MHz;): *δ* = 155.15 (C = O), 134.67 (CH), 116.73 (CH_2_), 72.27 (CH_2_), 69.46 (CH_2_), 67.78 (CH_2_), 41.87 (C), 22.58 (CH_2_), 7.45 (CH_3_). SEC (RI detection, CHCl_3_) *M*_n_: 2.1 kDa, *Đ*_M_ = 1.2.

#### 75% TMPAC/25% NTC

^1^H NMR (400 MHz, CDCl_3_): *δ* 6.15 (m, 1H), 5.93 (m, 1H), 5.83 (m, 3H), 5.26 (m, 6H), 4.41 (m, 2H), 4.10 (m, 12H), 3.40–4.00 (m, 13H), 3.32 (s, 6H), 2.93 (m, 2H), 2.30 (m, 1H), 1.81 (m, 1H), 1.49 (q, ^3^*J*_H–H_ = 9.4 Hz, 6H), 1.44–1.15 (m, 2H), 0.89 (m, 11H). ^13^C NMR (400 MHz, CDCl_3_): 155.14 (C = O), 134.68 (CH), 132.92 (CH), 116.73 (CH_2_), 106.93 (CH), 72.27 (CH_2_), 69.46 (CH_2_), 67.78 (CH_2_), 49.26 (CH_2_), 43.60 (CH), 41.87 (CH), 37.30 (C), 28.38 (C), 22.59 (CH_2_), 7.46 (CH_3_). SEC (RI detection, CHCl_3_) *M*_n_ = 2.0 kg·mol^−1^, *Đ*_M_ = 1.31

#### 50% TMPAC/50% NTC

^1^H NMR (400 MHz, CDCl_3_): *δ* 6.14 (m, 1H), 5.92 (m, 1H), 5.83 (m, 1H), 5.25 (m, 2H), 4.41 (m, 2H), 4.10 (m, 4H), 3.40–4.00 (m, 10H), 3.32 (s, 2H), 2.93 (m, 2H), 2.30 (m, 1H), 1.81 (m, 1H), 1.49 (q, ^3^*J*_H–H_ = 9.4 Hz, 2H), 1.44–1.15 (m, 2H), 0.89 (m, 5H). ^13^C NMR (400 MHz, CDCl_3_): 154.87 (C = O), 137.71(CH), 134.68 (CH), 132.66 (CH), 116.73 (CH_2_), 106.93 (CH), 72.27 (CH_2_), 69.46 (CH_2_), 67.78 (CH_2_), 49.26 (CH_2_), 43.60 (CH), 41.87 (CH), 37.30 (C), 28.38 (C), 22.59 (CH_2_), 7.46 (CH_3_). SEC (RI detection, CHCl_3_) *M*_n_ = 2.5 kg·mol^−1^, *Đ*_M_ = 1.37.

#### 25% TMPAC/75% NTC

^1^H NMR (400 MHz, CDCl_3_): *δ* 6.16 (m, 3H), 5.93 (m, 3H), 5.83 (m, 1H), 5.27 (m, 2H), 4.41 (m, 6H), 4.10 (m, 4H), 3.40–4.00 (m, 22H), 3.32 (s, 2H), 2.93 (m, 6H), 2.30 (m, 3H), 1.81 (m, 3H), 1.49 (q, ^3^*J*_H–H_ = 9.4 Hz, 2H), 1.44–1.15 (m, 6H), 0.89 (m, 6H). ^13^C NMR (400 MHz, CDCl_3_): 154.87 (C = O), 137.71(CH), 134.68 (CH), 132.66 (CH), 116.73 (CH_2_), 106.93 (CH), 72.27 (CH_2_), 69.46 (CH_2_), 67.78 (CH_2_), 49.26 (CH_2_), 43.60 (CH), 41.87 (CH), 37.30 (C), 28.38 (C), 22.59 (CH_2_), 7.46 (CH_3_). SEC (RI detection, CHCl_3_) *M*_n_ = 2.5 kg·mol^−1^, *Đ*_M_ = 1.34.

#### 100% NTC

^1^H NMR (400 MHz, CDCl_3_): *δ* 6.14 (m, 1H), 5.92 (m, 1H), 4.42 (m, 2H), 3.40–4.00 (m, 7H), 2.80–2.93 (m, 2H), 2.30 (m, 1H), 1.81 (m, 1H), 1.44–1.15 (m, 2H), 0.83 (m, 1H). ^13^C NMR (400 MHz, CDCl_3_): 154.62 (C = O), 137.08 (CH), 132.68 (CH), 106.84 (CH), 72.04 (CH_2_), 69.49 (CH_2_), 67.00 (CH_2_), 49.25 (CH_2_), 43.61 (CH), 42.22 (CH), 37.30 (C), 28.38 (CH_2_). SEC (RI detection, CHCl_3_) *M*_n_ = 2.6 kg·mol^−1^, *Đ*_M_ = 1.37.

### Synthesis of aliphatic poly(carbonate urethane)

In a representative synthesis of the poly((TMPAC-*co-*hexamethylene diurethane), PolyTMPAC (2 kDa, 5.0 g, 2.5 mmol) was dissolved in a round bottom flask containing dry THF at 60 °C under N_2_, to which hexamethylene diisocyanate (HDI) (1.0 g, 6.0 mmol) was added. The mixture was allowed to stir for 48 h, during which time the viscosity visually increased dramatically. At 48 h, the temperature was increased to 80 °C and allowed to stir for 12 h, at which time the entire solution was added to 50 mL MeOH. The solution was concentrated, washed with 1 M HCl twice and once with saturated brine solution, and collected as a highly viscous, transparent oil (5.94 g, 99%). ^1^H NMR (400 MHz, CDCl_3_): *δ* 5.77 (m, 1H), 5.17 (m, 2H), 4.01 (s, 4H), 3.85 (m, 2H), 3.24 (m, 2.5H), 3.06 (m, 0.5H), 1.54(m, 0.5H), 1.14–1.45 (m, 4H), 0.87 (t, ^3^*J*_H-H_ = 7.6 Hz, 3H). ^13^C NMR (400 MHz, CDCl_3_): *δ* 155.15 (C = O), 134.67 (CH), 116.73 (CH_2_), 72.27 (CH_2_), 69.46 (CH_2_), 67.78 (CH_2_), 41.87 (C), 40.82 (CH_2_), 29.88 (CH_2_), 26.17(CH_2_), 22.58 (CH_2_), 7.45 (CH_3_). SEC (RI detection, CHCl_3_) *M*_n_ = 3.5 kg·mol^−1^, *Đ*_M_ = 1.81.

### Synthesis of isophorone di(allyl urethane)

Isophorone diisocyanate (10.00 g, 0.045 moles) was added by canula transfer to a round bottom flask (dried 120 °C overnight and sealed) followed by dry THF (40 mL). Freshly distilled allyl alcohol (5.54 g, 0.095 moles), stored over molecular sieves, was added dropwise to the solution while stirring at 300 rpm. Upon complete transfer of the allyl alcohol, the reaction was heated to 50 °C and held isothermally for 24 h, at which point residual diisocyanate was quenched with water (at 50 °C). Crude urethane was obtained after dissolving the reaction mixture in ethyl acetate, washing with 1 M HCl (3 washes) and brine (1 wash) and concentrating the product. A viscous clear oil was collected after column chromatography (25:75 EtOAc:Hexane) and concentrated in vacuo to yield a colorless oil (1.2 g, 3.5 mmol, 7.8 %). ^1^H NMR (400 MHz, CDCl_3_): *δ* 5.85–5.94 (m, 2H), 5.18–5.30 (m, 4H), 4.86 (m, 1H), 4.53–4.55 (m, 5H), 3.79–3.82 (m, 1H), 2.91-2.92 (d, ^3^*J*_H–H_ = 3.0 Hz, 2H), 1.67–1.74 (t, ^3^*J*_H–H_ = 9.0 Hz, 2H), 1.17–1.21 (m, 2H), 0.83–1.05 (m, 11H), ^13^C NMR (400 MHz, CDCl_3_): 156.69 (C = O), 155.51 (C = O), 132.94 (CH), 117.72 (CH_2_), 65.59 (CH_2_), 54.89 (CH_2_), 47.05 (CH), 45.62 (CH_2_), 44.66 (CH_2_), 41.85 (CH_2_), 36.41 (C), 35.04 (CH_3_), 31.86 (C), 29.70(CH_3_), 27.63(CH_3_), 23.24(CH_3_). Mass spectrometry (ESI); *m/z* = 338.22 (M^+^). Elem. anal. Calcd for C_18_H_30_N_2_O_4_: C, 63.88; H, 8.93; N, 8.28%. Found: C, 63.81; H, 8.77; N, 8.24%.

### Formulation of poly(TMPAC) resins

Oligomer, reactive diluents and other diluents were added to a vial, along with the 4 arm tetrathiol (pentaerythritol tetrakis(3-mercaptopropionate) (PETMP)). After mixing, photointiator and photoinhibitor were added. As an example, the polyTMPAC resin consisted of isophorone di(allyl urethane) reactive diluent (13.78 g, 40.7 mmol), polyTMPAC (15.28 g, 7.6 mmol), 1,3,5-triallyl-1,3,5-triazine-2,4,6(1H,3H,5H)-trione as a second reactive diluent species (14.65 g, 58.7 mmol), PETMP (24.41 g, 53.2 mmol), and propylene carbonate as an unreactive diluent (16.54 g, 162.1 mmol) mixed together for 8 h at ambient temperature. To this was added Irgacure 819 (photoinitiator, 0.82 g, 1 wt%), and paprika extract (photoinhibitor, 0.50 g, 0.75 wt%) in a dark room with little ambient light, followed by 1 h of stirring. After homogenization of the resin, the resin was placed in a brown glass container and stored at room temperature in the dark.

### Spectroscopic analysis of thiol-ene crosslinking

Conversion of alkenes in the oligomeric and monomeric reactive components by reactions with the thiols in PETMP with 1% wt photoinitiator and no inhibitor were performed to study crosslinking kinetics. Experiments were performed in 0.5 mL CDCl_3_ at ambient conditions, exposed to *λ* = 340–430 nm light for discrete timepoints prior to storage in brown glass vials.

### Photorheology

Crosslinking kinetics of resin samples were examined by measuring the dampening or phase ratio (tan *δ*), storage moduli, loss moduli, complex viscosity, and film thickness to determine the gelation time(s)^63^. Resin samples were subjected to oscillatory shearing between two parallel plates (500 µm gap), one made of glass and transparent, at 1 Hz for 50 sec without irradiation, at which time the resins were irradiated with *λ* = 430–520 nm light and measurements were taken every 0.2 s over the course of 2 min. The inflection points of the moduli plots (storage and loss), and the peak tan *δ* values, were used to determine the time to gelation of the resin. Sample shrinkage was determined by measuring the gap between the plates at the same sampling rate as the other metrics.

### Mechanical testing

Printed modified ASTM Type IV dogbones were examined in uniaxial tensile testing at ambient moisture and temperature. Samples were placed in the tension clamps and allowed to vibrationally equilibrate for 10 min, at which point each sample was extended at 5 mm·min^−1^ until failure (defined as total sample failure). Seven samples were run per composition.

### Dynamic mechanical analysis

Rectangular dynamic mechanical analysis (DMA) samples were prepared via 3D printing sample bars (2.0 × 0.5 × 0.2 cm). Samples were analyzed in tension mode using the standard autotension mode, with a testing frequency of 1 Hz, a preload force of 1 N, and a static force of 0.1 N. Thermal sweeps were conducted at 2 °C·min^−1^. Samples were equilibrated at −30 °C for 5 min and were heated to 200 °C at a rate of 10 °C·min^−1^. The peak ratio between the loss and storage moduli (*E”*/*E’*, *tan δ*) was defined as the *T*_*g*_.

Relaxation kinetics studies of the printed scaffolds were conducted using submerged samples at 37 °C in phosphate buffered saline (PBS) solution, in compression mode. Printed porous scaffolds (1 cm^3^) were placed in compression and deformed 10 µm, 1 Hz with a preload of 0.1 N at ambient conditions (not submerged) for ~60 sec. At this time, the scaffold was then immersed in the PBS solution and held isothermally at 37 °C for 60 min. Storage moduli and tan *δ* values were recorded as a function of time to determine the behavior of the polymer during initial submersion/introduction to biologically mimicking conditions. Expansion forces were measured using the same method in creep mode.

Shape memory experiments were performed using the same porous scaffolds in compression mode. The samples were equilibrated at 60 °C for 1 h, deformed by ∼30% (load dependent deformation) and cooled to −20 °C. Once the sample was isothermal with the cooled chamber, the load was removed, and the sample expansion was monitored as a function of force and displacement of the compression clamp as the sample was heated to 60 °C at 10 °C·min^−1^. Testing was performed in triplicate.

### 3D printing

Scaffolds based upon previously reported geometries were printed from polycarbonate resins using optimized conditions (dependent upon composition)^63,64^. Resins were added in 10 mL quantities to the resin tray, allowing for complete and even coverage of the printer optical window on the surface of the printing plate. Porous scaffolds were printed by exposing resins to *λ* = 405 nm light using a custom-built digital light processing unit and printing parameters were individually determined for each resin composition through optimization of irradiance, irradiation time, resulting film thickness, and semi-quantified feature resolution (percentage of theoretical resolution), and were further optimized in the printing vat as necessary^59^. The z-stage step transition was set to 100 µm, and each slice was exposed for 6 s, on average. Print resolution was determined through image analysis (Image J) of the theoretical structure, and pore size analysis using microscopy from the printed structure. The final structures are rinsed with acetone to remove residual resin and photoinhibitor, as denoted by colour removal.

### Degradation analysis

Porous scaffolds and nonporous scaffolds were immersed in degradation solution, following previously established protocols for static degradation analysis^17^. For dynamic degradation studies, films were tested using the DMA in tension mode with the autotensioner, loaded with a 0.1 N preload and 10 Hz oscillation. Films were immersed in 5 M NaOH solution at 37 °C for the duration of testing. Samples were tested until failure, with the phase ratio and the storage moduli recorded over the course of the study.

For in vivo degradation, samples were removed from subcutaneous tissue and sterilized using EtOH. Tissue was removed and scaffolds were extracted with hexane or methanol over a 48 h period, after which the extracted solutions were concentrated down and dissolved in either CDCl_3_ or DMSO-*d6*. Scaffold swelling ratio was determined by Eq. 1:

1 $${\rm{Swelling}}\; {\rm{ratio}}=\frac{({m}_{{\rm{f}}}-{m}_{{\rm{i}}})}{{m}_{{\rm{i}}}}$$

where *m*_i_ is the original mass of the scaffold (dry) and m_f_ is the mass of the scaffold after swelling (but blotted dry to remove droplets or excess solvent). The crosslink density, and therefore the remaining mass of the material, was determined by Eq. 2:

2 $${\rm{Gel}}\; {\rm{fraction}}( \% )=\frac{{m}_{{\rm{f}}}}{{m}_{{\rm{i}}}}$$

where *m*_f_ is the final scaffold mass (dry) and *m*_i_ is the original scaffold mass (dry).

### Printed void filling

A hexagonal void was produced in Solidworks, and the cross-sectional area was varied to produce irregular voids, one which is sharply irregular and the other possessing rounded edges. The voids were printed and used for studying void-filling behavior, using cross-sectional area of the void and the printed scaffold (cube) to determine void filling as a qualitative function of shape.

### Expansion forces in alginate gels

Alginate was dissolved in water at a concentration of 10 mg·mL^−1^, to which was added 5 mL of calcium chloride dihydrate (0.1 mg·mL^−1^). The two components were mixed until gelation, and 10 mL of H_2_O was added as the gels were incubated at 37 °C overnight. Gel mechanical properties were matched adipose and glandular tissue using literature protocol^42^. Gels were cut with an eye-shaped opening, in the same manner as a lumpectomy surgery. Cubic scaffolds were shape fixed at 60% strain and inserted into the opening, where void filling and gel deformation were examined optically using the same cross-sectional analysis described for the “Printed Void Filling” section. The shape fixation behavior of the scaffold was further examined upon removal of the scaffold from the gel, and the shape recovery efficiency compared with the void-filling behavior, as well as the deformation of the alginate. The thin-walled computational molds previously described were then examined using determined loading forces and compared with the deformation found in alginate gels. An interior force of 1 N was initially applied uniformly to the interior (cut) surface of the gel in the same manner as the scaffold would be in contact and expand. The force was then scaled until deformation matched experimental results.

### Cytocompatibility and cellular analysis

Samples for cell culture studies (*n* = 4) were prepared by spin coating a solution of 0.4 wt% polymer in CHCl_3_ on a glass coverslip (1 min at 1000 rpm). Spin-coated glass coverslips were then sterilized by immersion in a 70% ethanol solution, fully dried before use, and placed into 12-well plates. NOR-10 (murine fibroblasts), Hs 792 (human fibroblasts), IC21 (murine macrophages), and D16 (murine adipocytes) cell lines were purchased from ATCC UK and cultured in DMEM (NOR-10 and Hs 792), RPMI-1640 (IC21), and DMEM/F12 (D16) media supplemented with 10% FBS (20% for NOR-10) and 1% pen/strep, at 37 °C and 5% CO_2_. 1% L-Alanyl-L-Glutamine was added in DMEM/F12 medium. MC3T3 (murine pre-osteoblasts) were purchased from Public Health England and cultured in Alpha Minimum Essential Medium with ribonucleosides, deoxyribonucleosides, 2 mM L-glutamine and 1 mM sodium pyruvate, but without ascorbic acid, 10% FBS and 1% pen/strep, at 37 °C and 5% CO_2_.

### Cell proliferation

Cell proliferation assays were performed on spin-coated glass slides by seeding the above cell lines (*n* = 4, 2000 cells cm^−2^) and measuring metabolic activity at selected timepoints (24 h, 3, 7, and 14 days of culture). Cell proliferation was evaluated by using a PrestoBlue® metabolic assay following the supplier’s instructions. Briefly, after removing the medium, 1 mL of PrestoBlue® solution (10% in cell culture medium) was added to each well, followed by incubation at 37 °C for 1–4 h. 100 µL of solution was taken from each well and placed in triplicate into a 96-well plate. The fluorescence intensity (FI) was detected in a BMG Labtech Fluostar Omega Microplate Reader at wavelengths of 590 nm for excitation and 610 nm for emission.

### Cell spreading

Cells were seeded on spin-coated coverslips (*n* = 4) at 4000 cells·cm^−2^. After 72 h, cells were fixed using a 4% paraformaldehyde solution for 10 min, permeabilized using 0.5% Triton X-100 in cytoskeleton stabilization (CS) buffer (0.1 M PIPES, 1 mM EGTA, and 4% (w/v) 8000 MW polyethylene glycol) at 37 °C for 10 min, rinsed thrice for 5 min each in CS buffer, and incubated in 0.1% sodium borohydride in PBS at ambient temperature for 10 min to quench aldehyde autofluorescence. Samples were then blocked in 5% donkey serum for 20 min at 37 °C and incubated overnight at 4 °C with mouse primary anti-vinculin antibody (Abcam, 1:100). Samples were then washed three times with 1% donkey serum for 5 min each, and then incubated with Alexa Fluor 647 Phalloidin for cytoskeleton staining (1:200) for 1 h followed by Alexa Fluor® IgG-594 secondary antibody (Invitrogen, donkey anti-mouse, 1:100). DAPI was used to stain the cell nuclei. Cells were imaged with a FV3000 Olympus confocal fluorescence microscope using 350, 594, and 633 nm excitation filters and a ×20 or ×40 objectives^65^.

### 3D cell experiments

3D-printed scaffolds were sterilized by immersion in 70% ethanol, dried, and then placed in 24-well plates, and incubated for 24 h in cell culture medium at 37 °C, 5% CO_2_. The medium was then removed and cells (100,000 in 20 μL of medium) were seeded on top of the scaffolds (*n* = 3) and incubated at 37 °C, 5% CO_2_ for 3 h. After this time, 2 mL of culture medium was added and the cells were incubated again at 37 °C, 5% CO_2_ for the selected timepoints (24 h, 3 days, 7 days). A live/dead assay (Invitrogen) was performed at each of the selected timepoints. Briefly, scaffolds were washed with PBS (3 × 2 mL) and incubated with a calcein/ethidium homodimer solution at 25 °C for 20 min, following the supplier’s instructions. Scaffolds were then washed with PBS (3 × 2 mL) and placed on a microscope slide for fluorescent imaging. Cells were imaged with a FV3000 Olympus confocal fluorescence microscope using 488 nm and 594 nm excitation filters and a ×4 air objective^65^. Image J was used for analysis.

### Surgical procedure

Experiments were performed in accordance with the European Commission Directive 2010/63/EU (European Convention for the Protection of Vertebrate Animals used for Experimental and Other Scientific Purposes) and the United Kingdom Home Office (Scientific Procedures) Act (1986) with project approval from the institutional animal welfare and ethical review body (AWERB). Anaesthesia was induced in adult male (8 weeks old) Sprague Dawley rats (200–300 g) with isofluorane (2–4%; Piramal Healthcare) in pure oxygen (BOC). Animals were placed prone onto a thermocoupled heating pad (TCAT 2-LV; Physitemp), and body temperature was maintained at 36.7 °C. The experimental material and control material (PLLA) were implanted over either the spinotrapezius or lateral aspect of the external obliques. Following an incision of ∼3 cm, the skin was separated from the muscle with large forceps, and any excess fat was removed. The implants were tunnelled under the skin and placed in direct contact with the muscle, at sites distal to the incision. The order of the implants was randomized but constrained so that each implant appeared in each location bilaterally at least once. The wounds were sealed with a subcuticular figure of 8 purse string suture with a set-back buried knot using 3-0 vicryl rapide suture (Ethicon). The surgical procedure was performed under the strictest of aseptic conditions with the aid of a nonsterile assistant. Post-surgical analgesia was administered, and rats were placed into clean cages with food and water *ad libitum*.

### Histological analysis

At 1-month and 2-month timepoints, samples were excised from the subcutaneous tissue and fixed with 4% paraformaldehyde for 24 h. After fixation, samples were washed with increasing percentages of ethanol (70–100%) for 30 min each, washed thrice with xylene, and embedded in paraffin wax blocks for sectioning. Slices (10–30 µm thick) were cut using a Leica Biosystems microtome for histological analysis before being stained using hematoxylin and eosin stains or Masson’s Trichrome staining using protocols available through Sigma–Aldrich. Analysis was performed using light microscopy (Leica, ×4 and ×10 objectives) and image stitching was performed in ImageJ (NIH, Bethesda, MD). Brightfield images were analyzed and qualitatively assessed for general inflammation compared to PLLA control samples. Samples were also analyzed for a number of inflammatory cells utilizing a modified scoring system designed by the International Organization for Standardization (ISO 10993-6 Annex E). Scoring was based on a scale from 0 to 4 (0 = none; 1 = Rare, 1–5 Minimal; 2 = 5–10, Mild; 3 = Heavy Infiltrate, Moderate; 4 = Packed, Severe).

### Statistics and reproducibility

Statistical analysis of thermal, thermomechanical, and degradation results was performed using a standard one-way Student’s *t*-test, with probabilities of 0.05 used to assess the probability of differences between compositional behaviors. Specific reproduction numbers and experimental iterations are included in Figure captions and methodology text.

### Reporting summary

Further information on research design is available in the Nature Research Reporting Summary linked to this article.

## Supplementary information

Supplementary Information

Description of Additional Supplementary Files

Supplementary Video 1

Supplementary Video 2

Supplementary Video 3

Reporting Summary

## Data Availability

The authors declare that full experimental details and characterization of materials are available in the Supplementary Information. All raw data that support the findings in this study are available from the corresponding authors upon request.
