# Supplementary material for: 4D polycarbonates via stereolithography as scaffolds for soft tissue repair
Source: Nat Commun. 2021 Jul 5;12:3771. doi: 10.1038/s41467-021-23956-6 (PMC8257657; doi:10.1038/s41467-021-23956-6)
Supplement: Supplementary file 1 — Supplementary Information [file 41467_2021_23956_MOESM1_ESM.docx]

Supplementary Information: 4D Polycarbonates *via* Stereolithography as Scaffolds for Soft Tissue Repair

Andrew C. Weems, Maria C. Arno, Wei Yu, Robert T. R. Huckstepp, Andrew P. Dove

**Table of Contents**

*Spectroscopic Analysis 3*

*Polycarbonate Characterization 18*

*Printed Scaffold Morphological Characterization 20*

*Cellular Proliferation and Imaging 21*

*Thermomechanical Characterization 24*

*Shape Memory Characterization 26*

*Gravimetric Analysis 28*

*Histology Analysis 29*

**Spectroscopic Analysis**

**
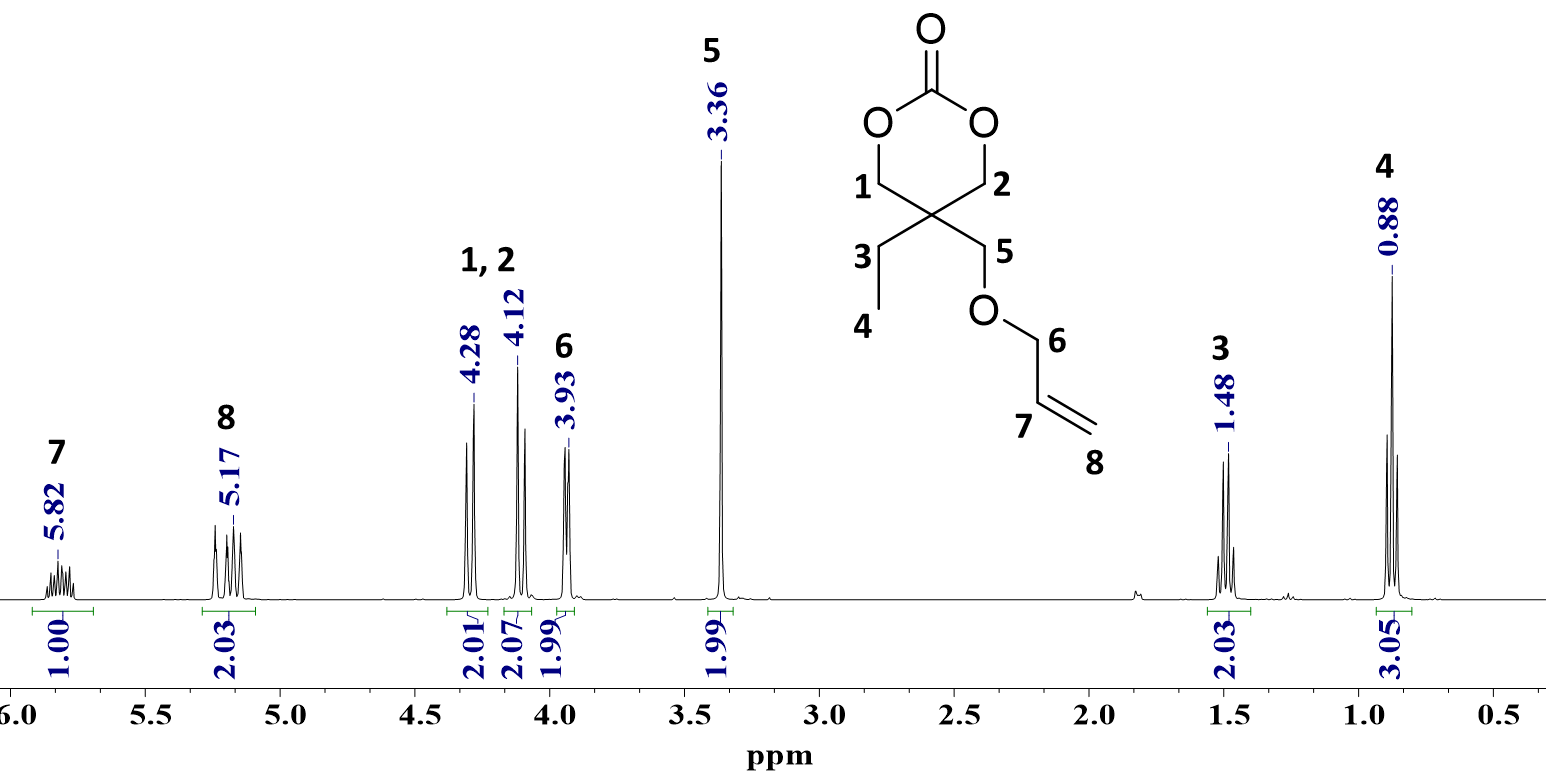
**

**Supplementary Figure 1**. ^1^H NMR spectrum of TMPAC monomer (CDCl_3_, 400 MHz, 300 K).

***
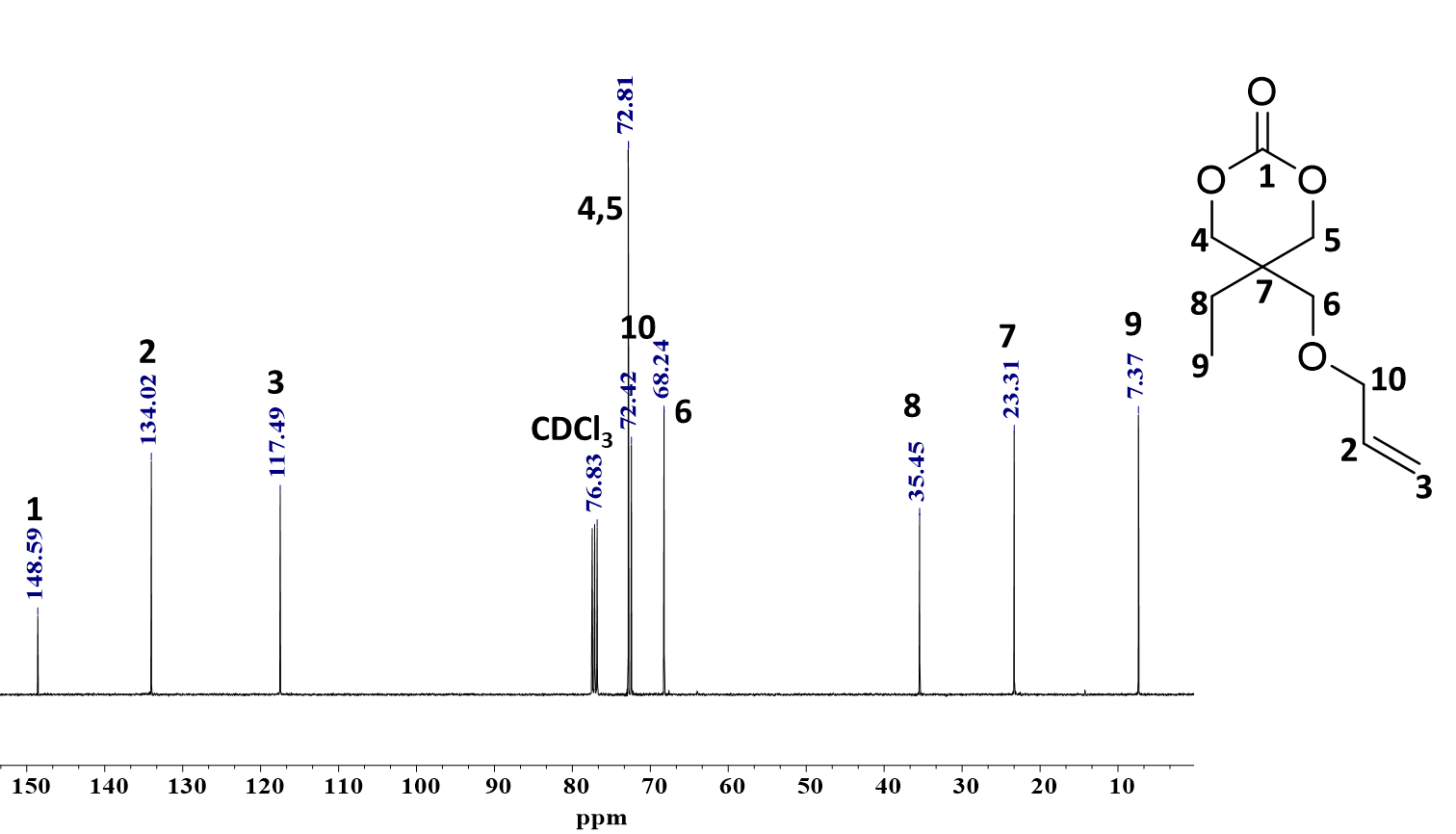
***

**Supplementary Figure 2**. ^13^C NMR spectrum of TMPAC monomer (CDCl_3_, 100 MHz, 300 K).

***
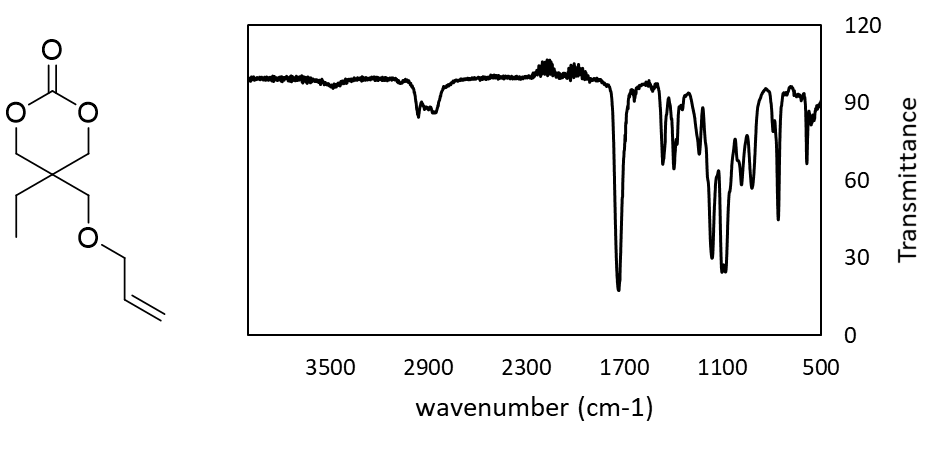
***

**Supplementary Figure 3**. FTIR spectrum of TMPAC monomer.


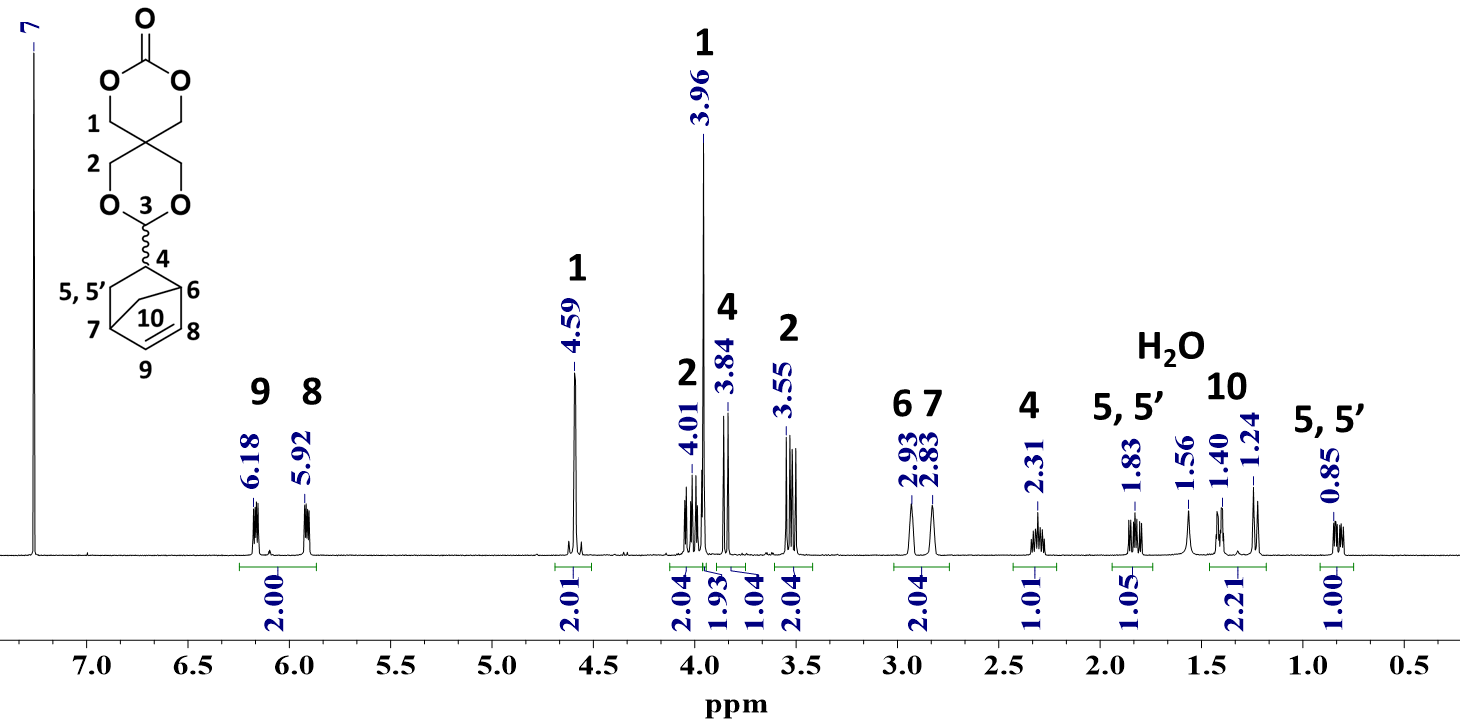


**Supplementary Figure 4**. ^1^H NMR spectrum of NTC monomer (CDCl_3_, 400 MHz, 300 K).

***
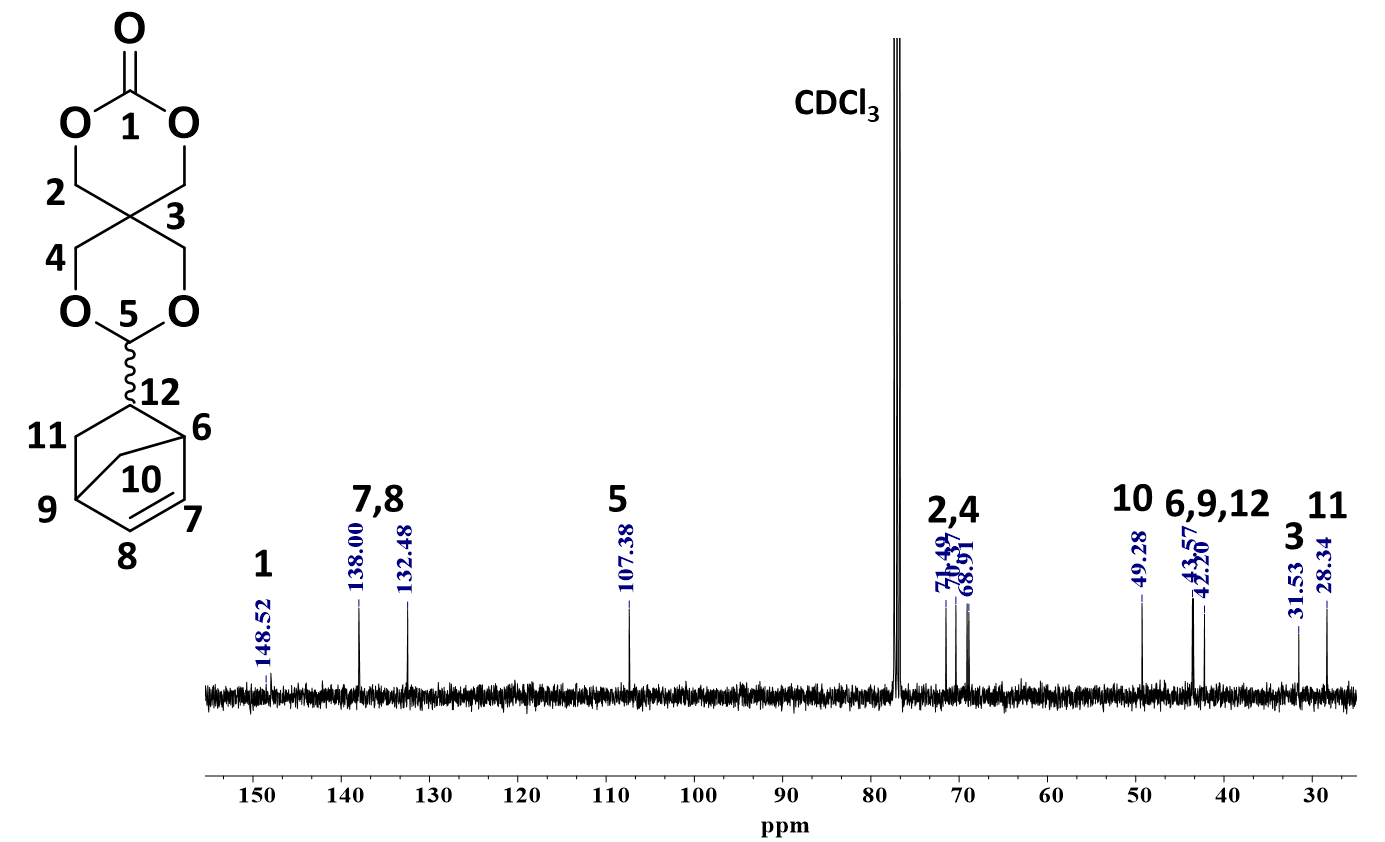
***

**Supplementary Figure 5**. ^13^C NMR spectrum of NTC monomer (CDCl_3_, 100 MHz, 300 K).

***
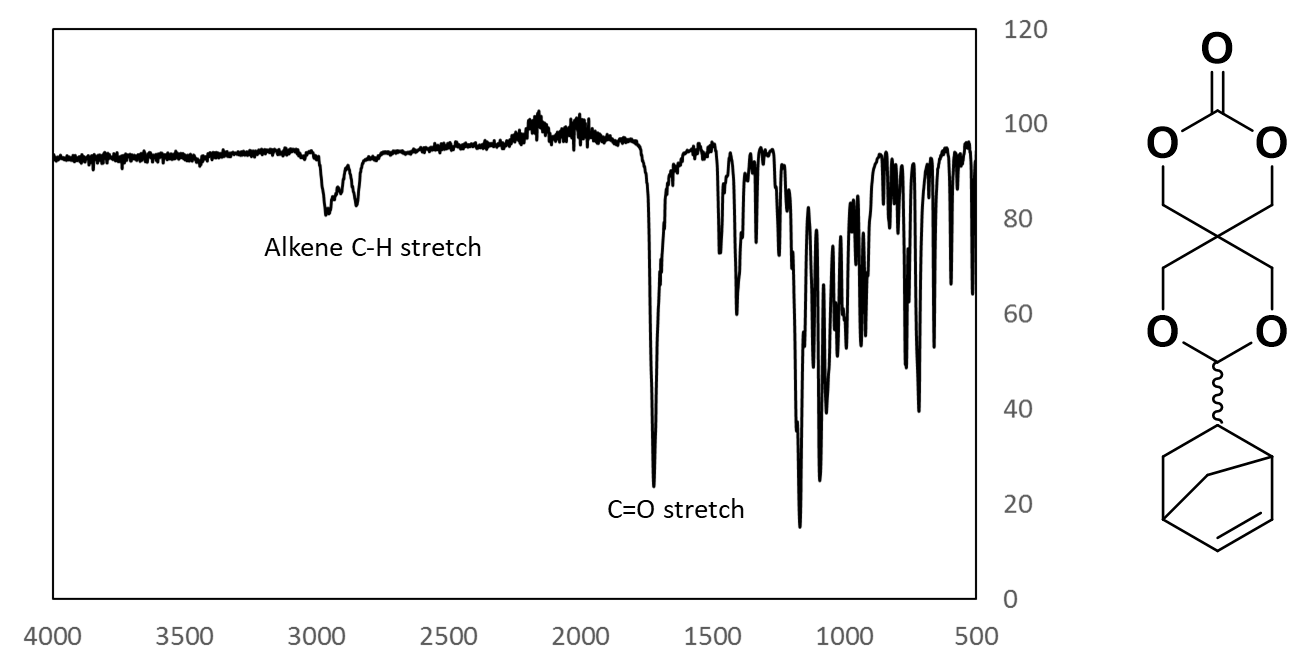
***

**Supplementary Figure 6**. FTIR spectrum of NTC monomer.


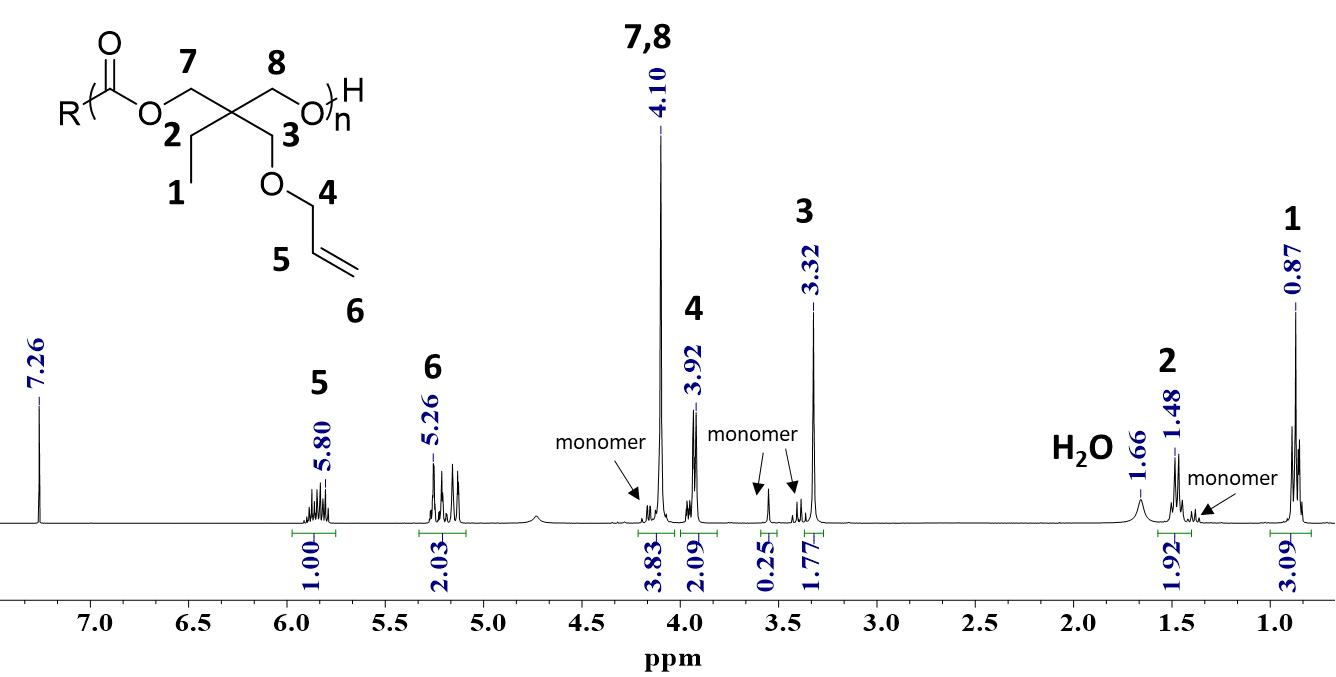


**Supplementary Figure 7**. ^1^H NMR spectrum of as-used polyTMPAC (CDCl_3_, 400 MHz, 300 K).


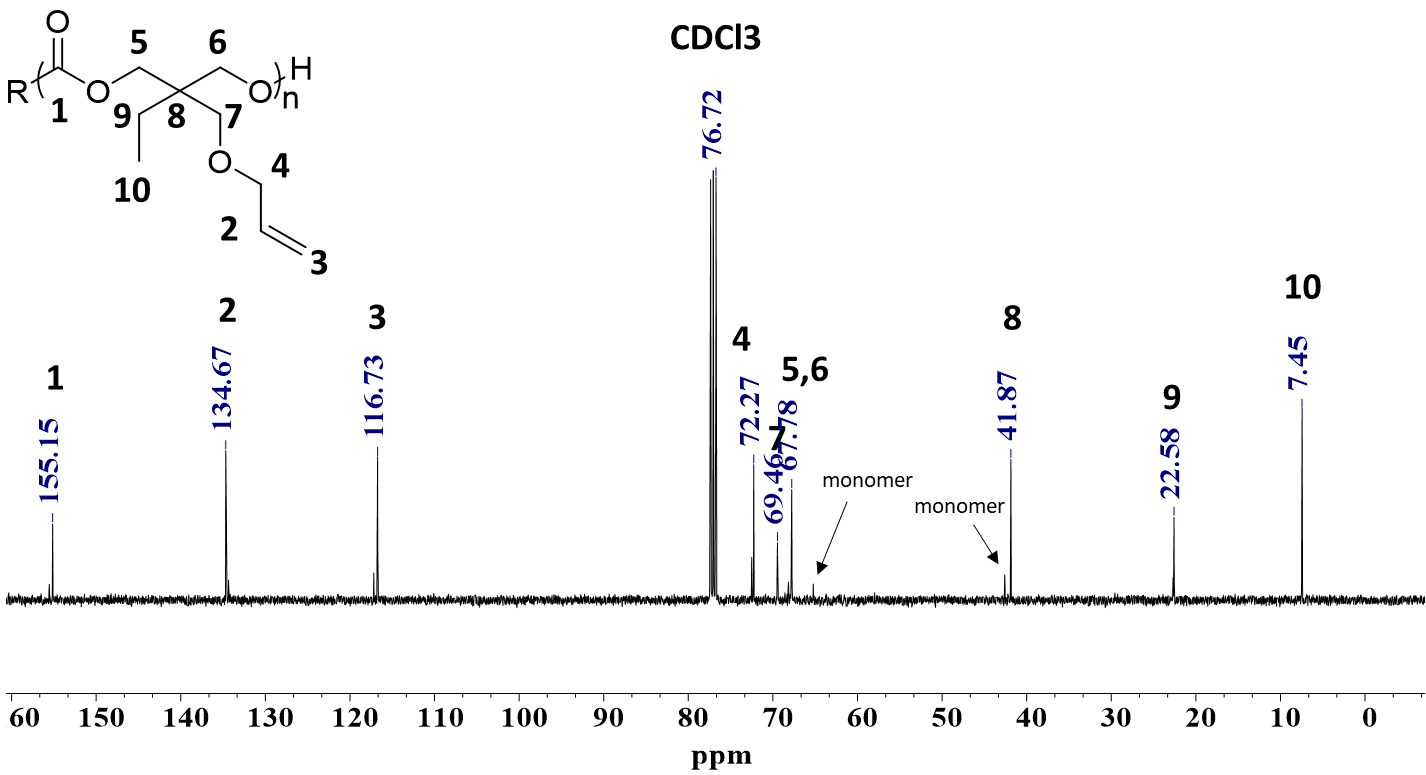


**Supplementary Figure 8**. ^13^C NMR spectrum of as-used polyTMPAC (CDCl_3_, 100 MHz, 300 K).


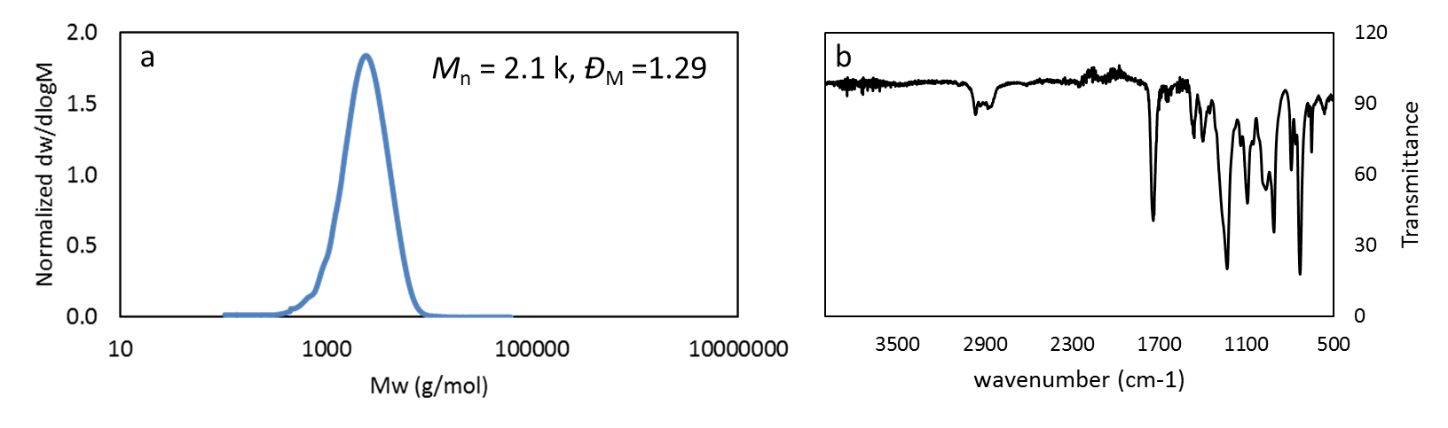


**Supplementary Figure 9**. (a) Size exclusion chromatogram and (b) FTIR spectrum of as-used polyTMPAC.


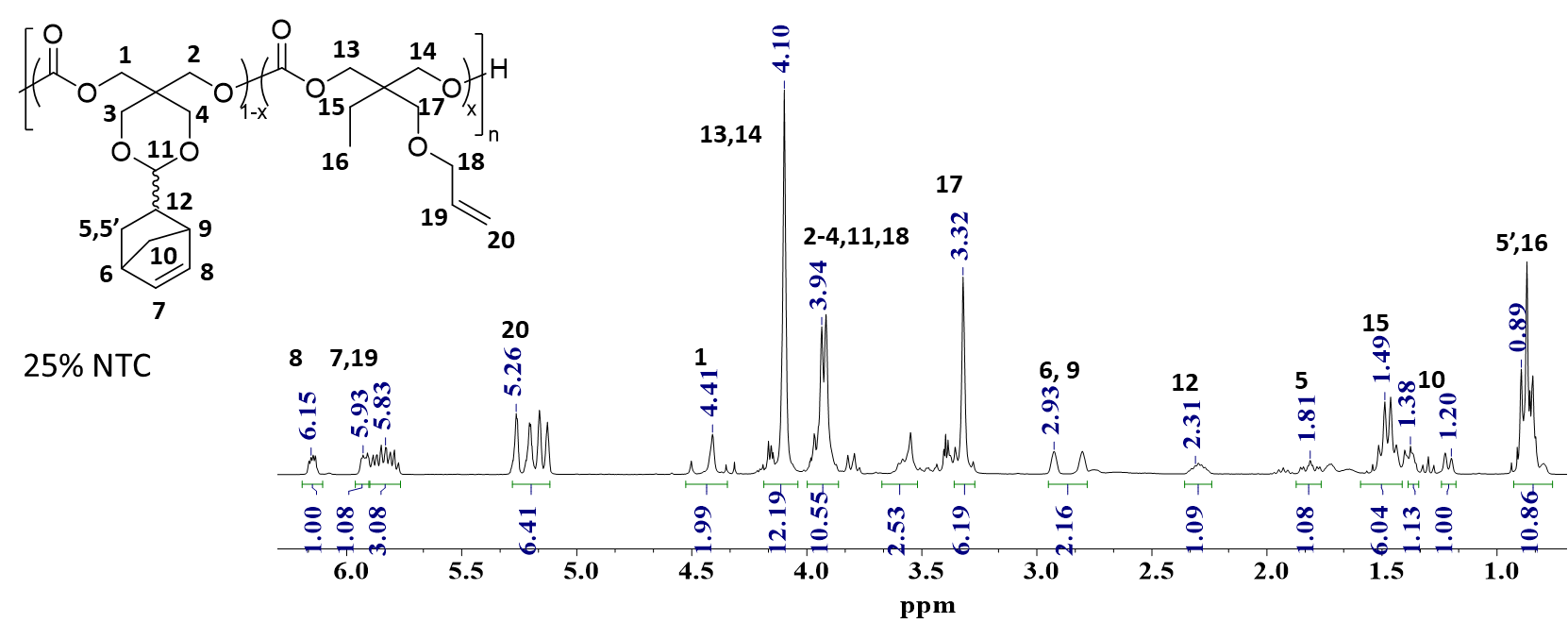


**Supplementary Figure 10**. ^1^H NMR spectrum of as-used poly(TMPAC-co-NTC) 75%TMPAC:25% NTC (CDCl_3_, 400 MHz, 300 K).


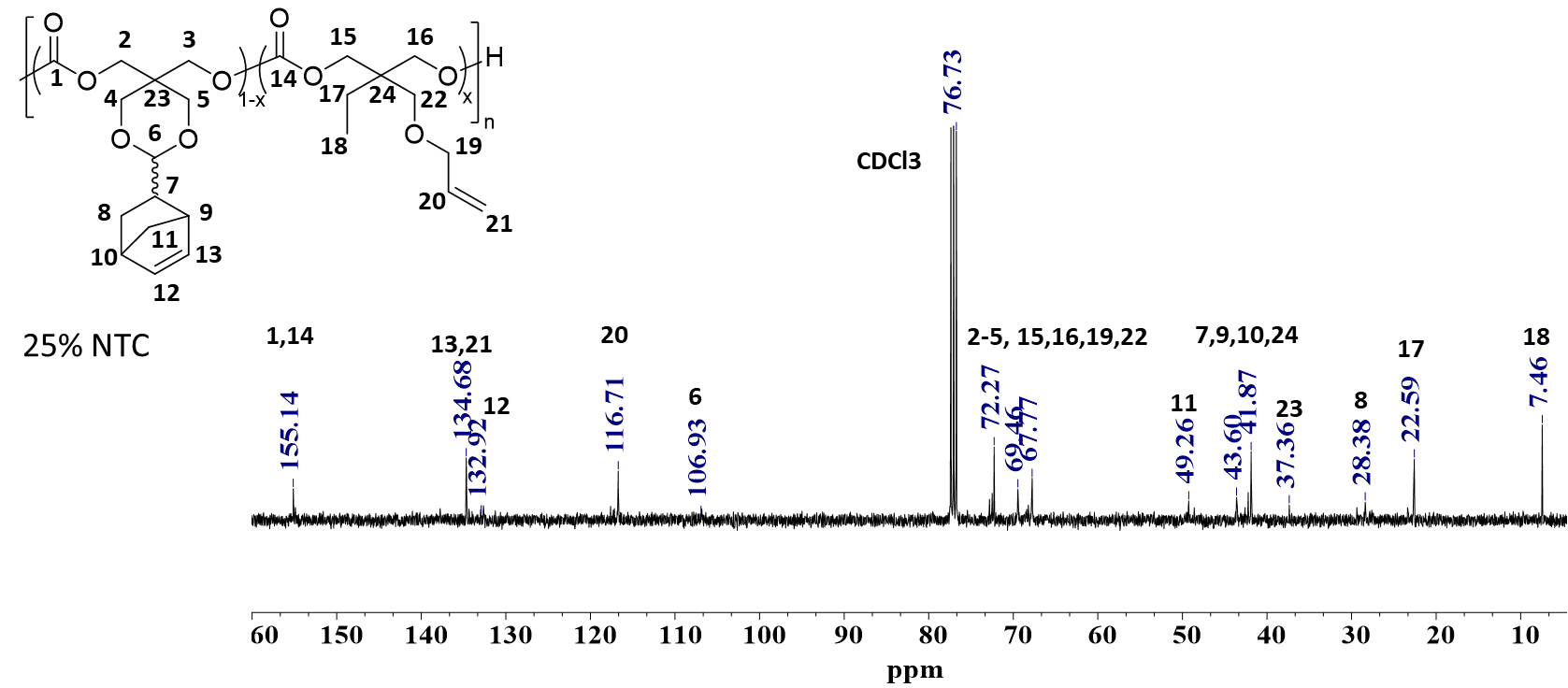


**Supplementary Figure 11**. ^13^C NMR spectrum of as-used poly(TMPAC-co-NTC) 75%TMPAC:25% NTC (CDCl_3_, 100 MHz, 300 K).


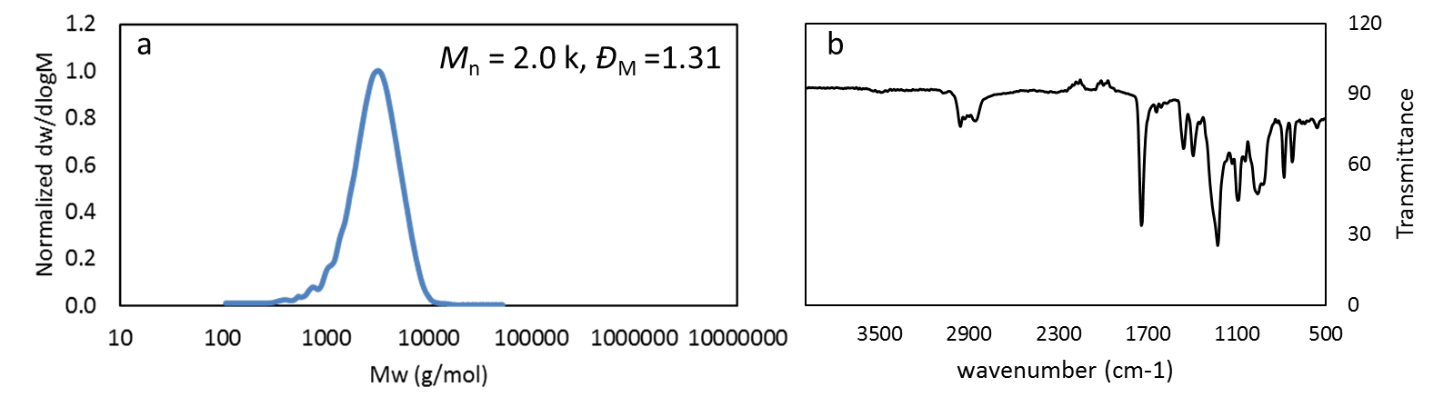


**Supplementary Figure 12**. (a) Size exclusion chromatogram and (b) FTIR spectrum of as-used poly(TMPAC-co-NTC) 75%TMPAC:25% NTC.


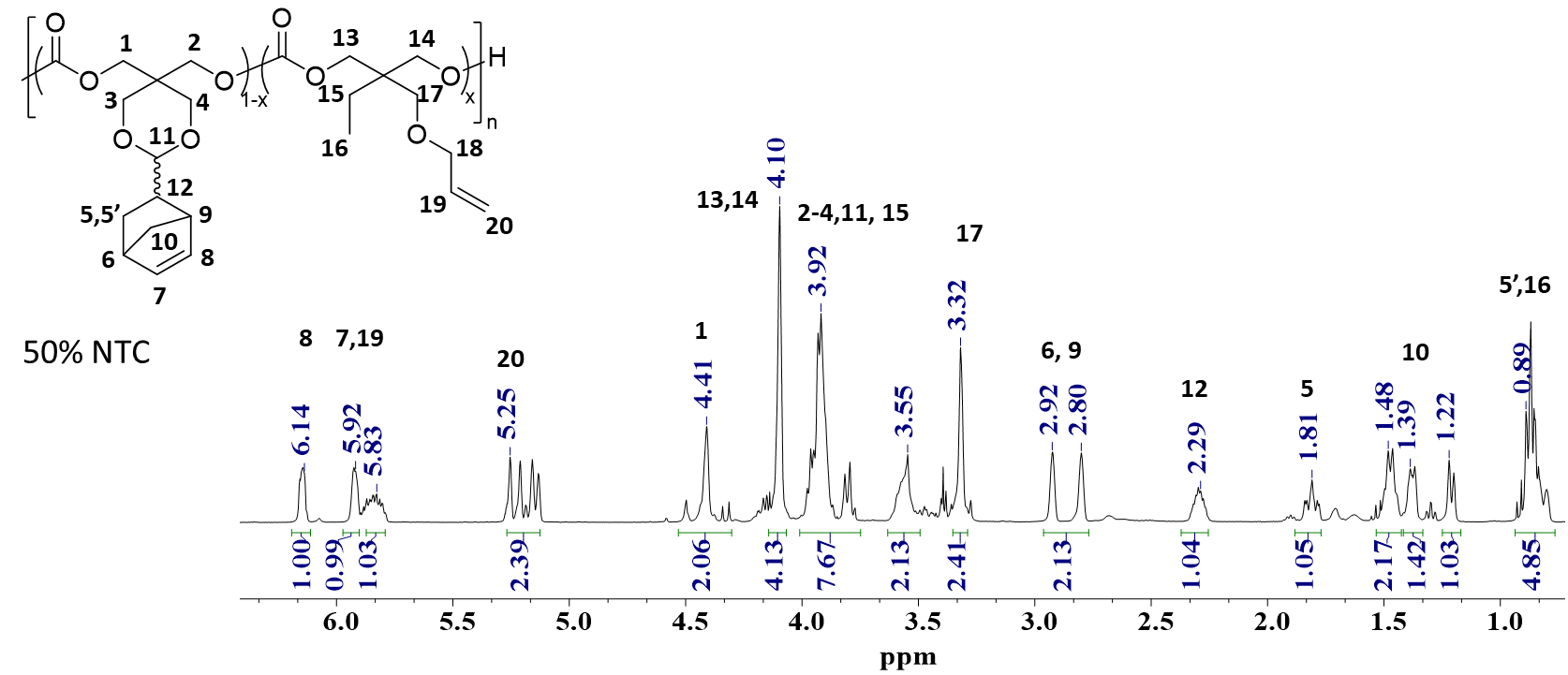


**Supplementary Figure 13**. ^1^H NMR spectrum of as-used poly(TMPAC-co-NTC) 50%TMPAC:50% NTC (CDCl_3_, 400 MHz, 300 K).


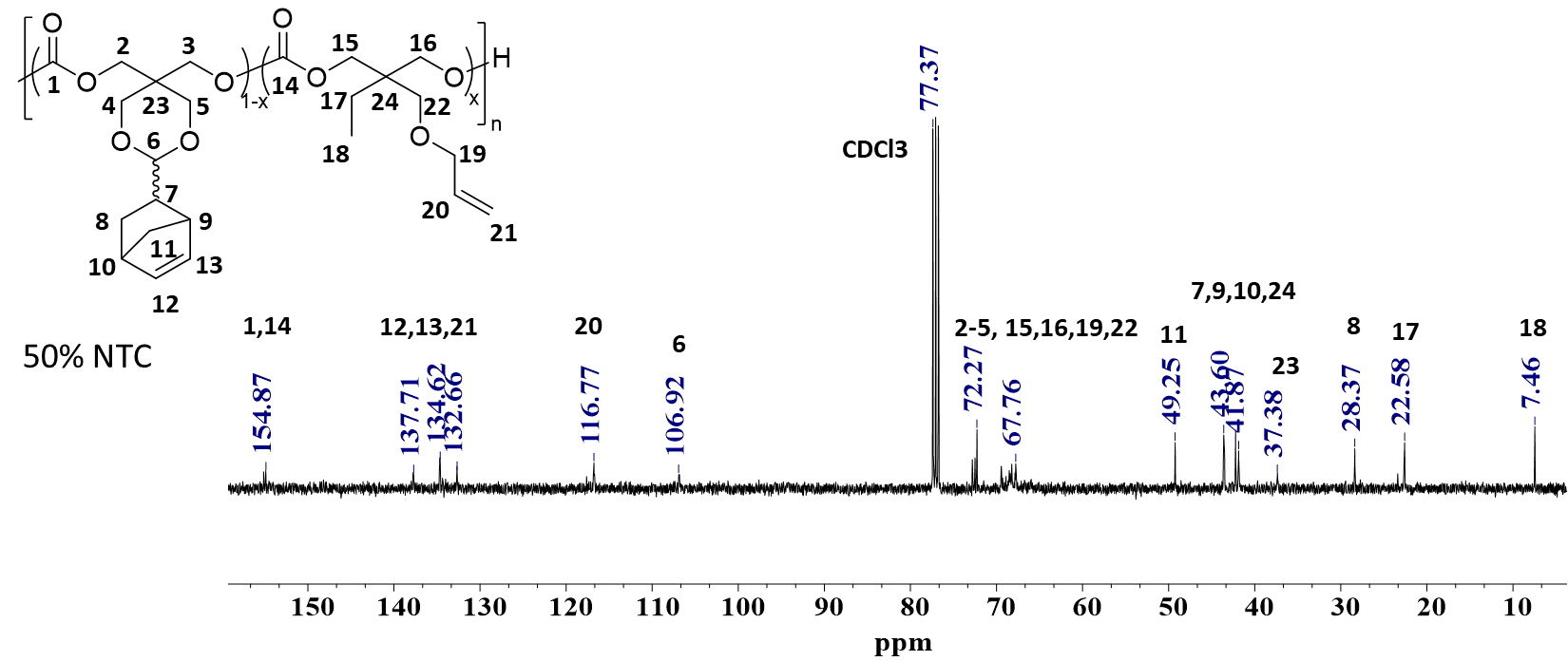


**Supplementary Figure 14**. ^13^C NMR spectrum of as-used poly(TMPAC-co-NTC) 50%TMPAC:50% NTC (CDCl_3_, 100 MHz, 300 K).


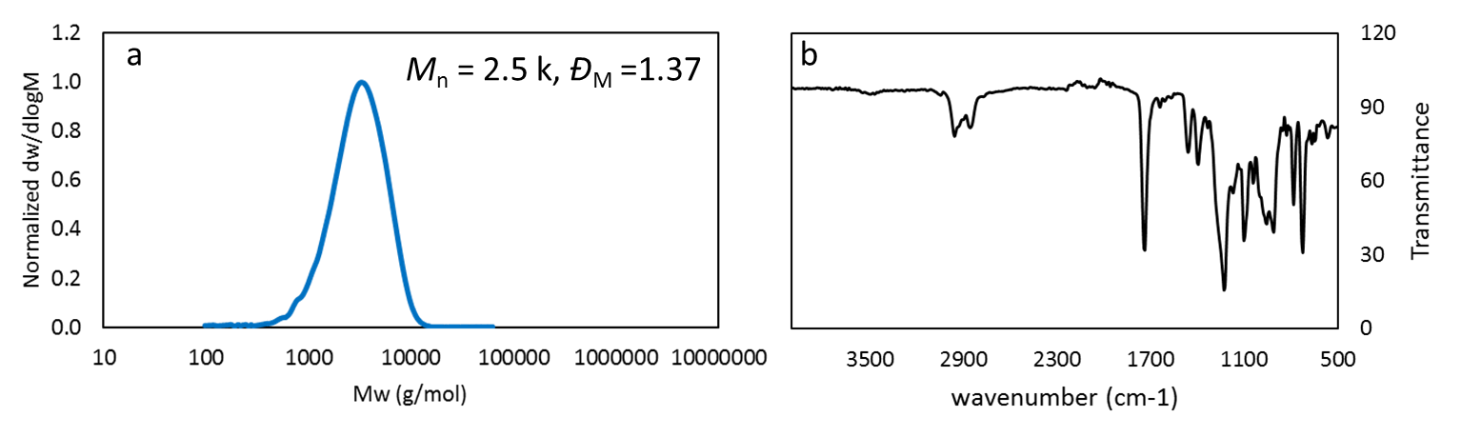


**Supplementary Figure 15**. (a) Size exclusion chromatogram and (b) FTIR spectrum of as-used poly(TMPAC-co-NTC) 50%TMPAC:50% NTC.


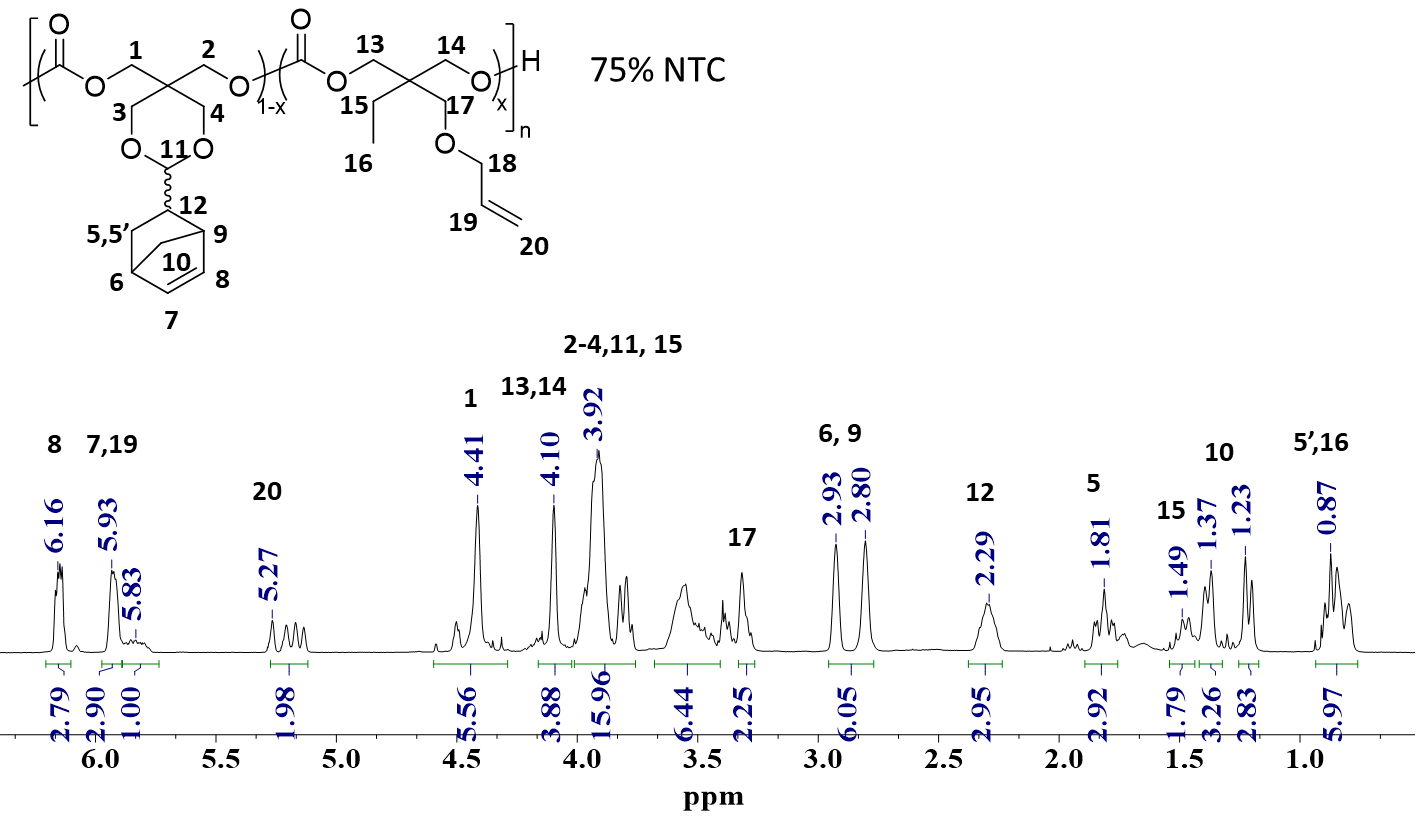


**Supplementary Figure 16**. ^1^H NMR spectrum of as-used poly(TMPAC-co-NTC) 25%TMPAC:75% NTC (CDCl_3_, 400 MHz, 300 K).


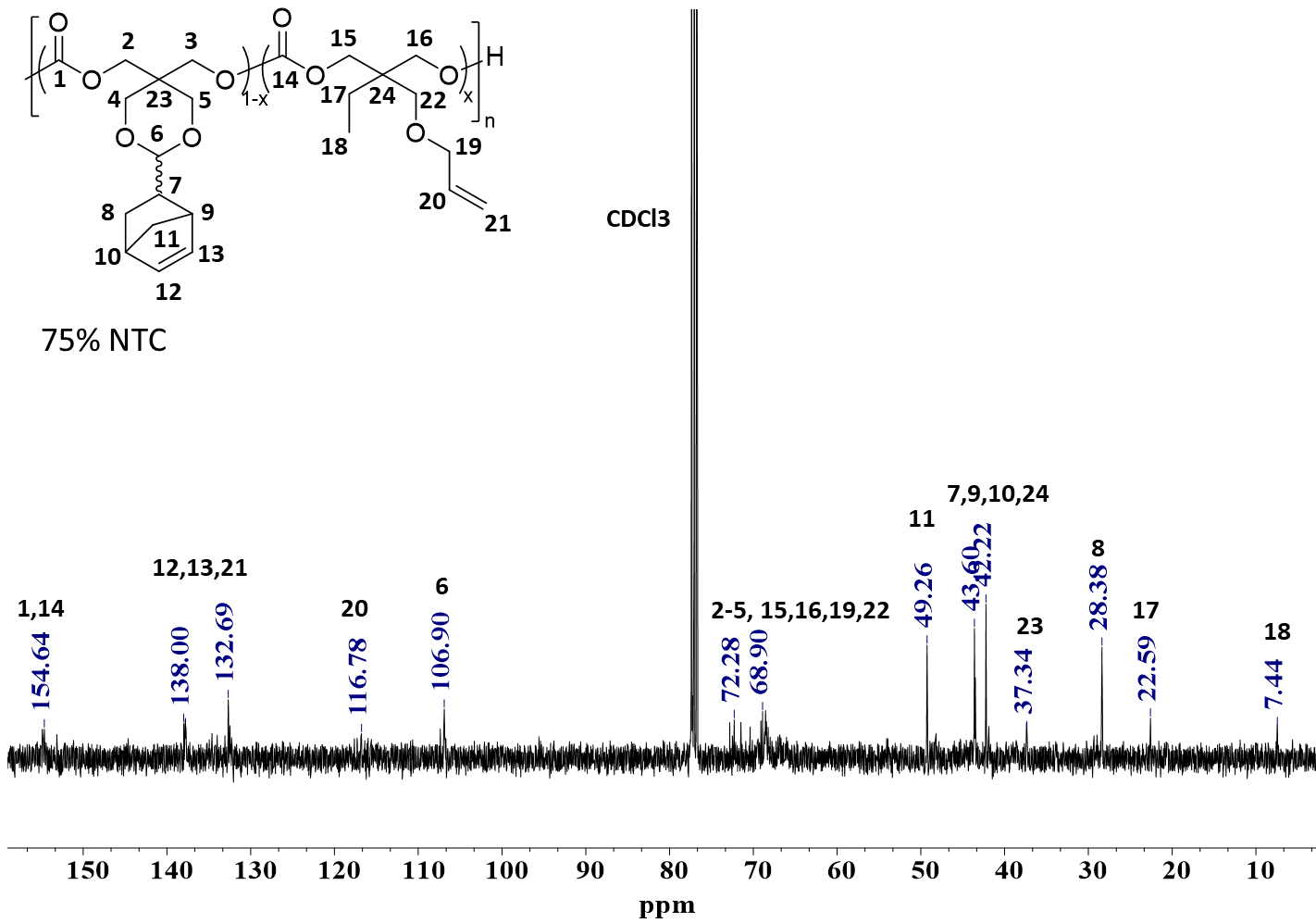


**Supplementary Figure 17**. ^13^C NMR spectrum of as-used poly(TMPAC-co-NTC) 25%TMPAC:75% NTC (CDCl_3_, 100 MHz, 300 K).


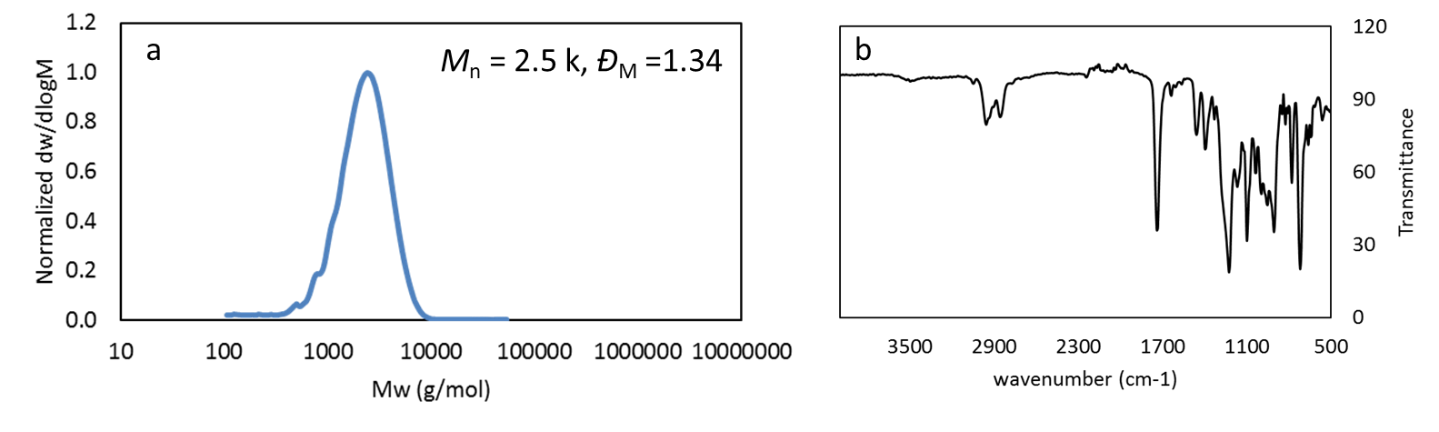


**Supplementary Figure 18**. (a) Size exclusion chromatogram and (b) FTIR spectrum of as-used poly(TMPAC-co-NTC) 25%TMPAC:75% NTC.


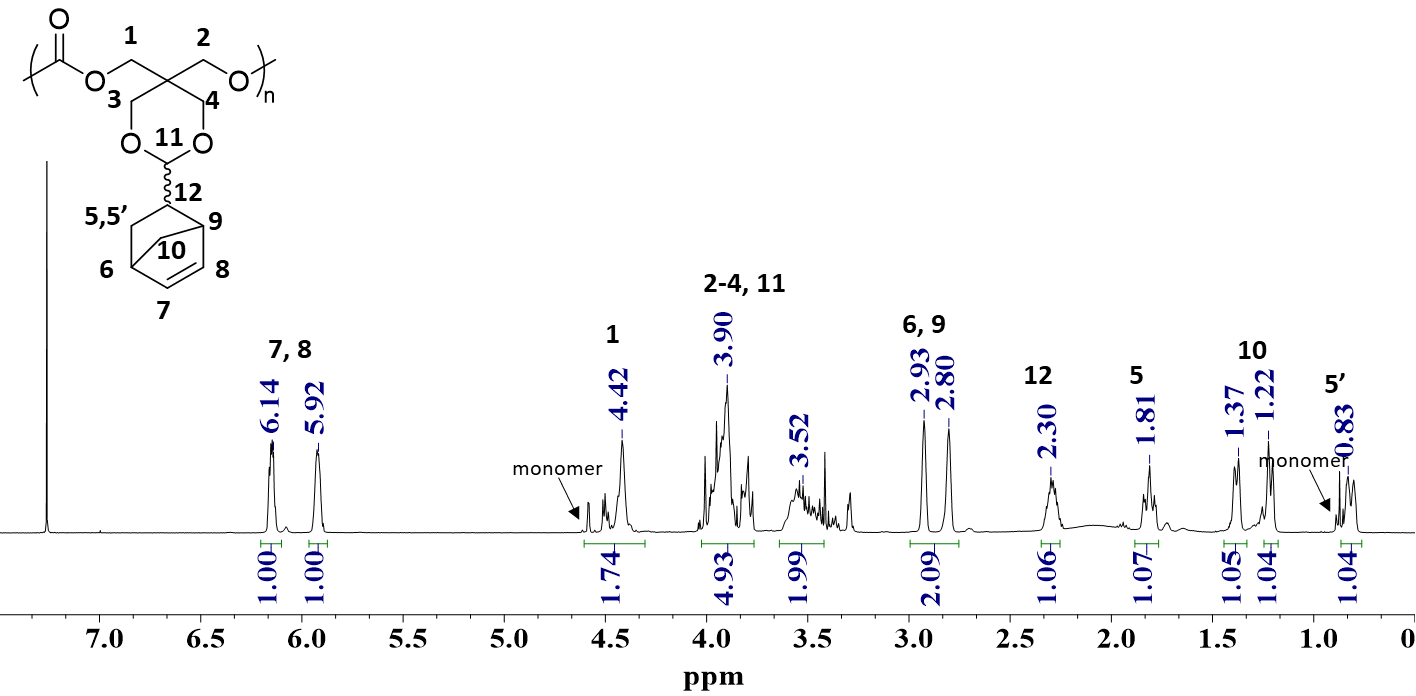


**Supplementary Figure 19**. ^1^H NMR spectrum of as-used polyNTC (CDCl_3_, 400 MHz, 300 K).


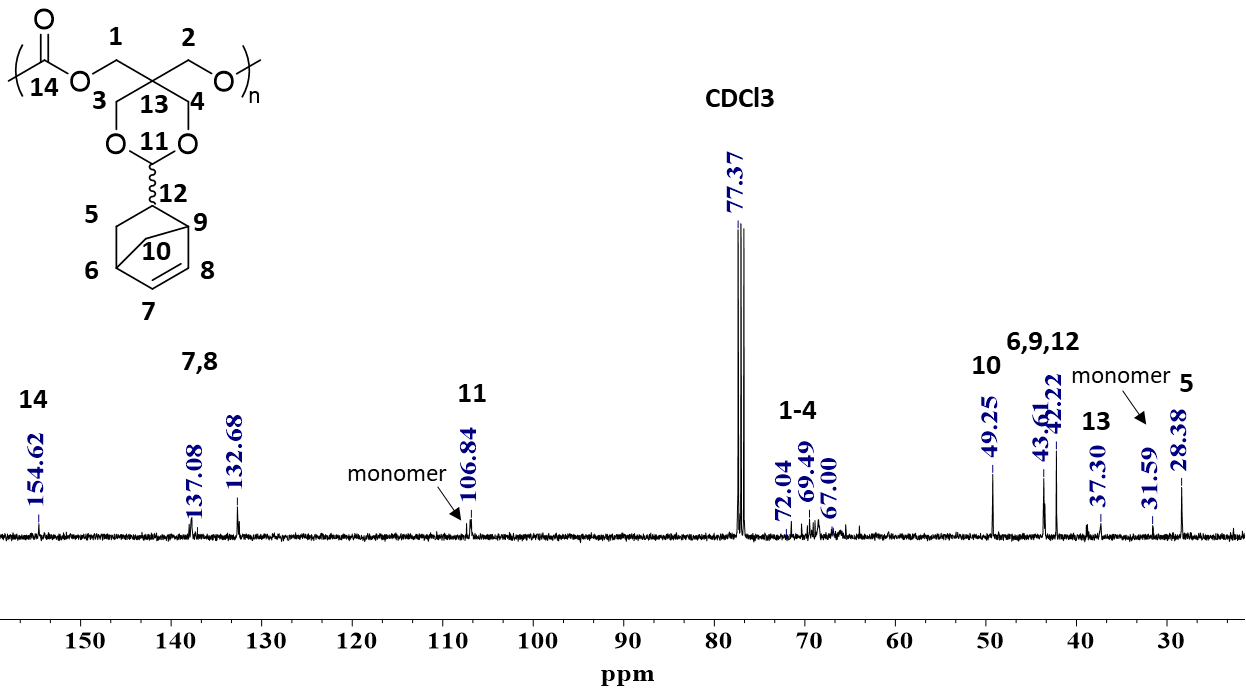


**Supplementary Figure 20**. ^13^C NMR spectrum of as-used polyNTC (CDCl_3_, 100 MHz, 300 K).


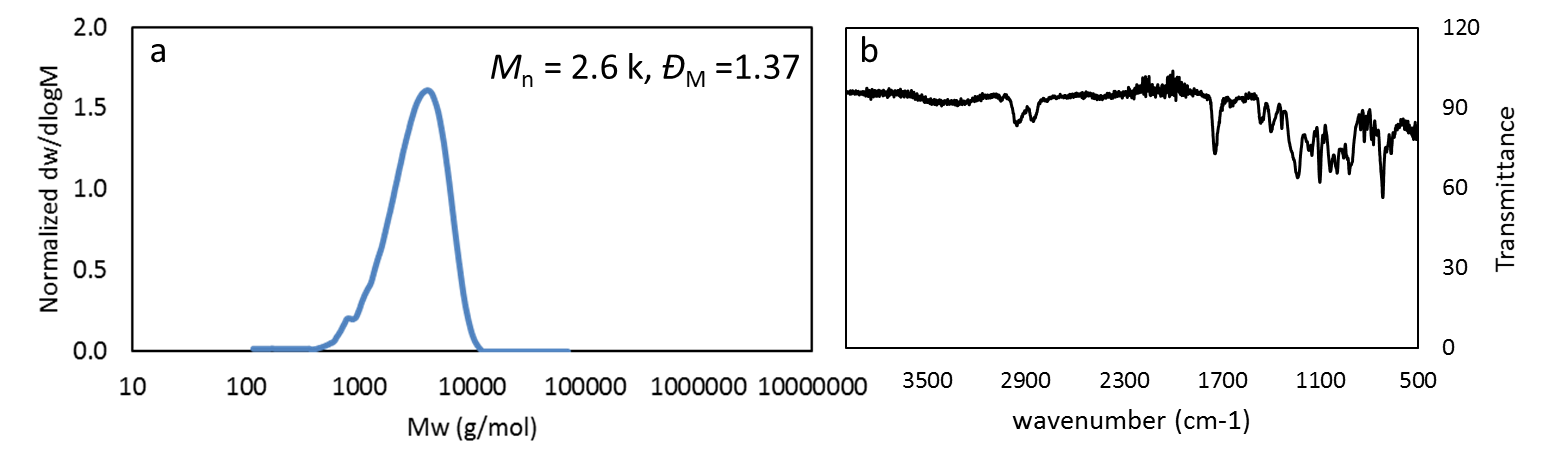


**Supplementary Figure 21**. (a) Size exclusion chromatogram and (b) FTIR spectrum of as-used polyNTC.


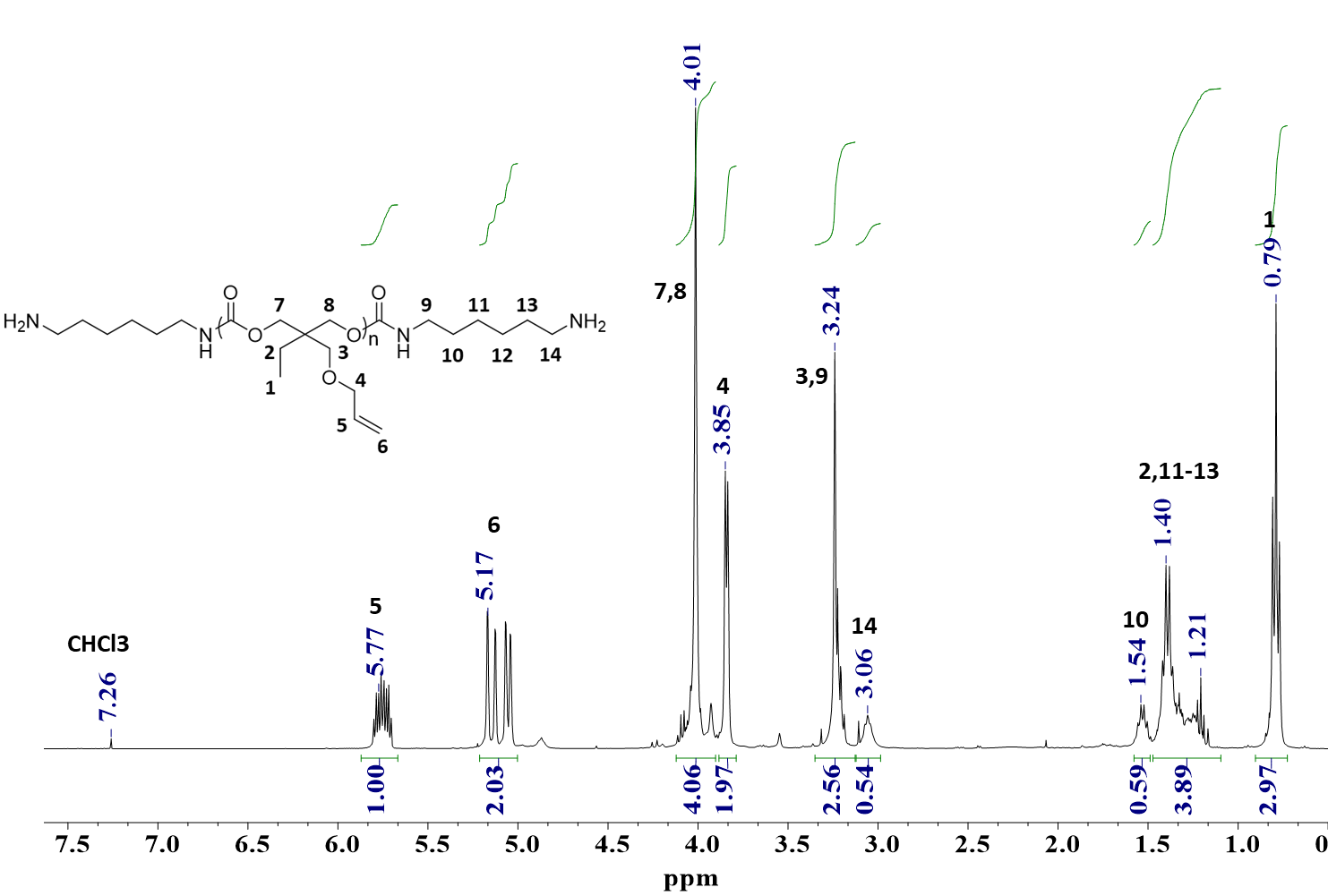


**Supplementary Figure 22**. ^1^H NMR spectrum of polyTMPAC chain extended with hexamethylene diisocyanate (CDCl_3_, 400 MHz, 300 K).


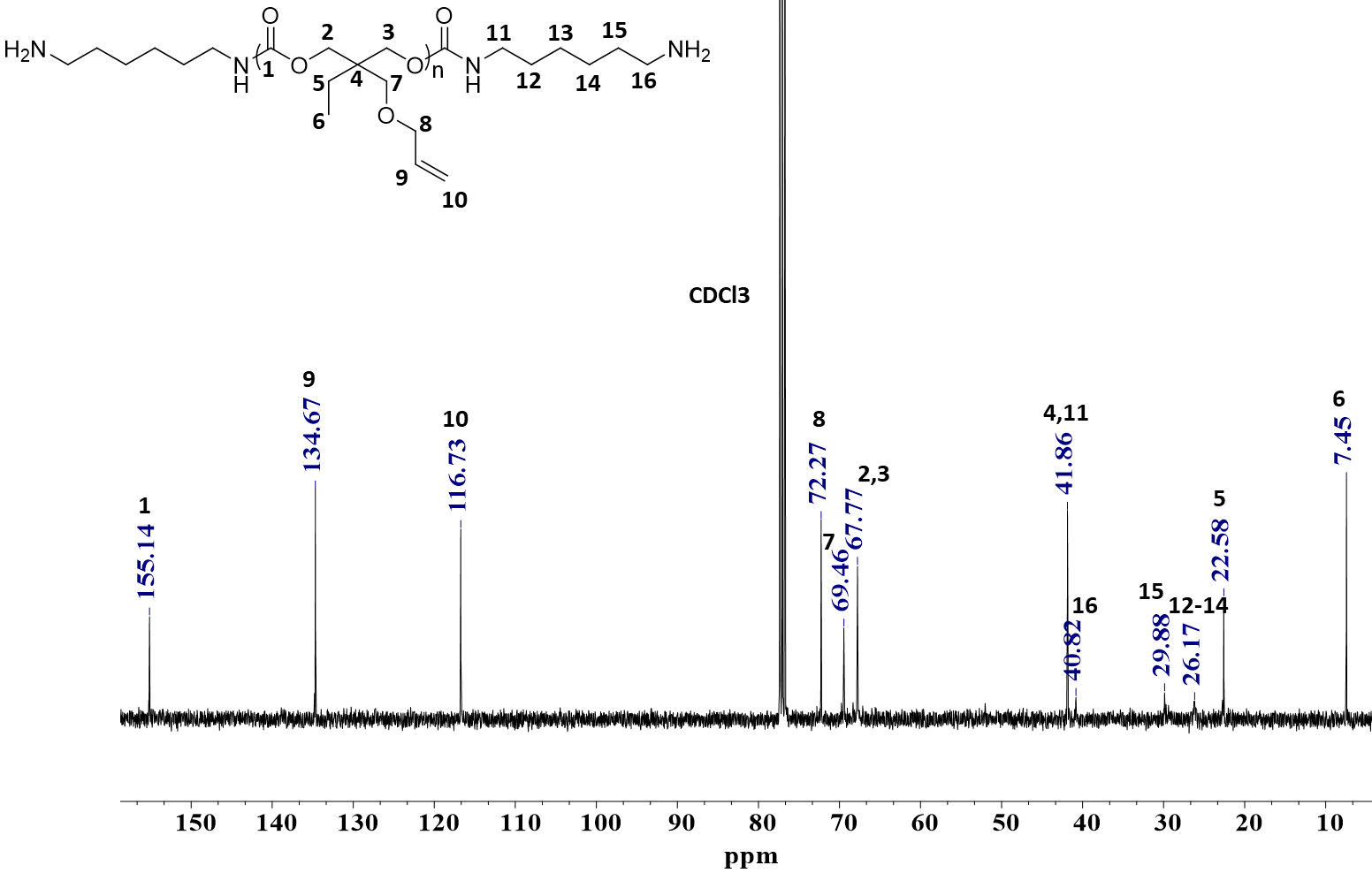


**Supplementary Figure 23**. ^13^C NMR spectrum of polyTMPAC chain extended with hexamethylene diisocyanate (CDCl_3_, 100 MHz, 300 K).


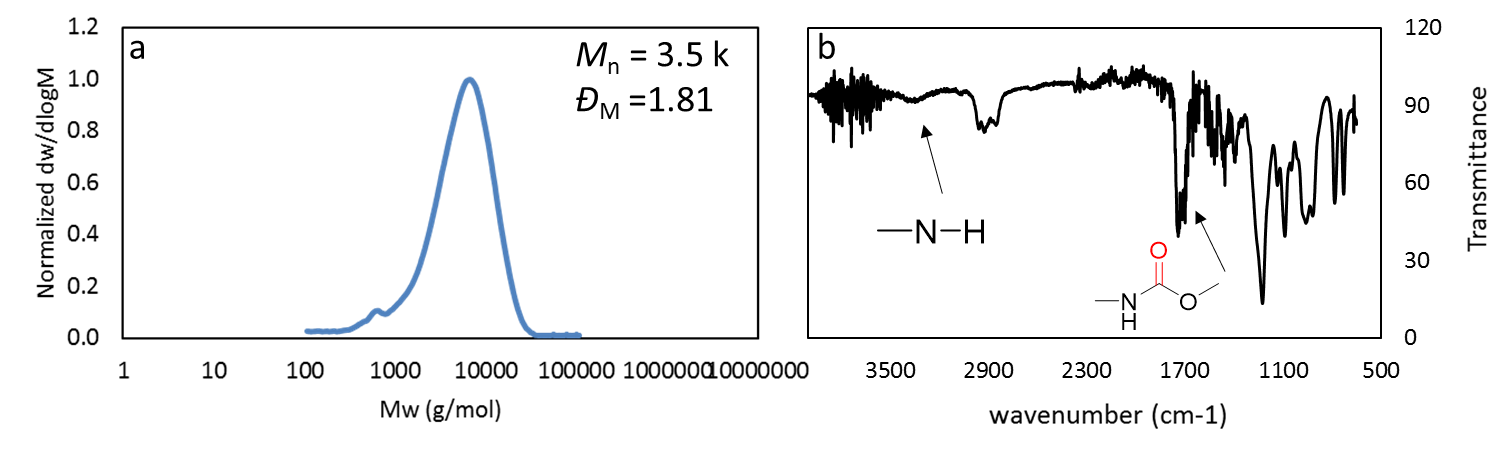


**Supplementary Figure 24**. (a) Size exclusion chromatogram and (b) FTIR spectrum of polyTMPAC chain extended with hexamethylene diisocyanate.


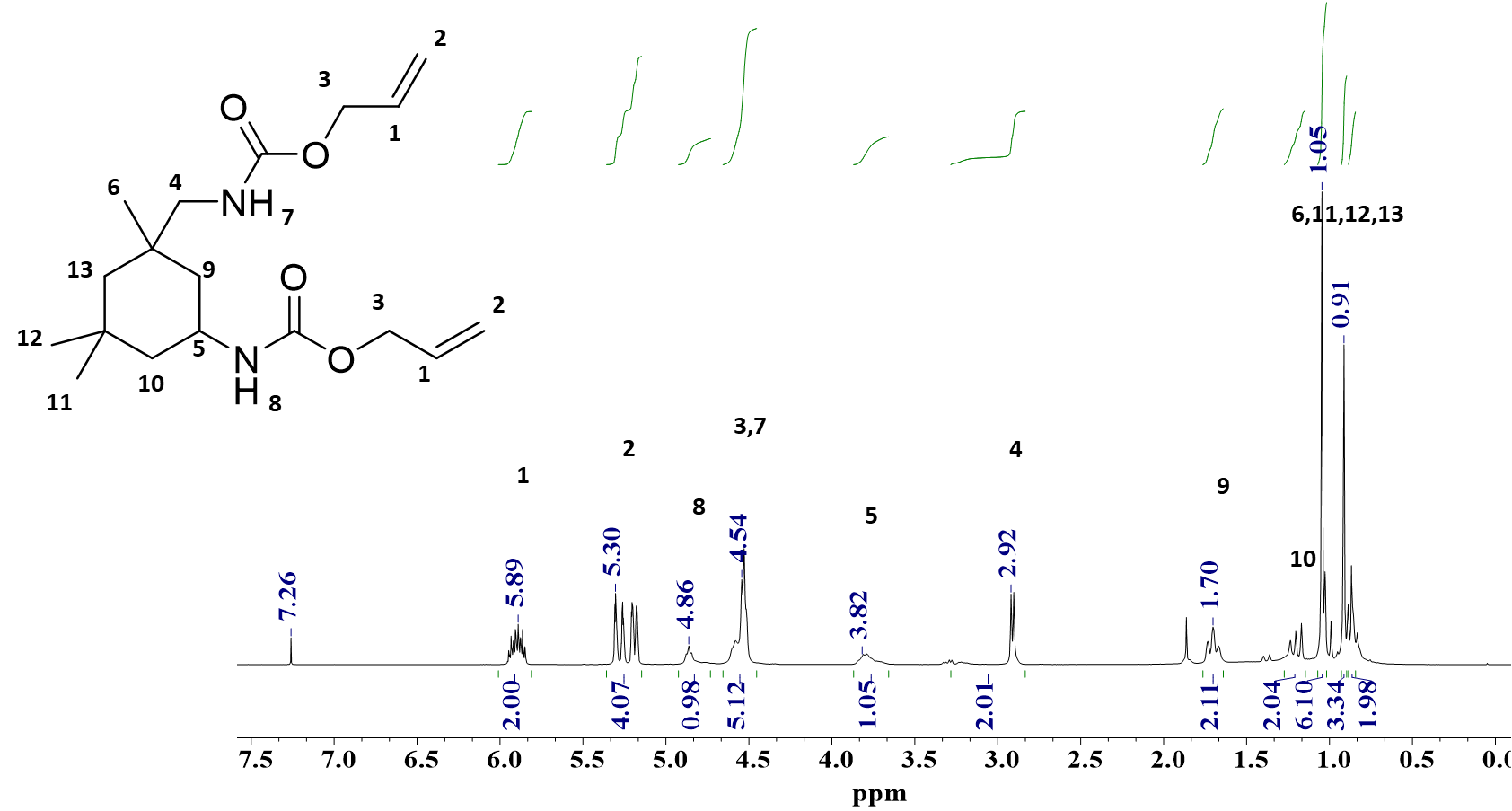


**Supplementary Figure 25**. ^1^H NMR spectrum of isophorone di(allyl urethane) monomer (CDCl_3_, 400 MHz, 300 K).


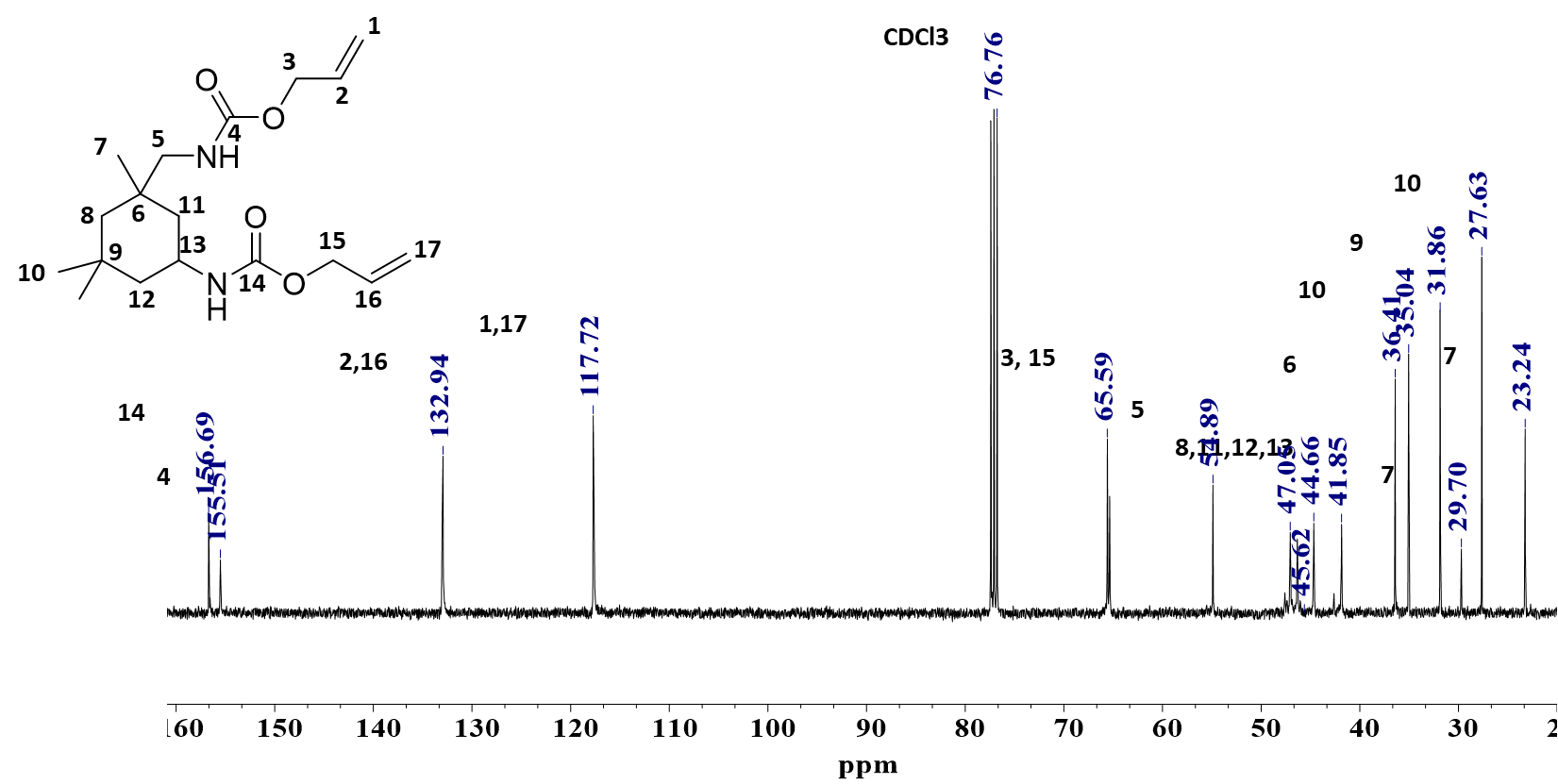


**Supplementary Figure 26**. ^13^C NMR spectrum of isophorone di(allyl urethane) monomer (CDCl_3_, 100 MHz, 300 K).


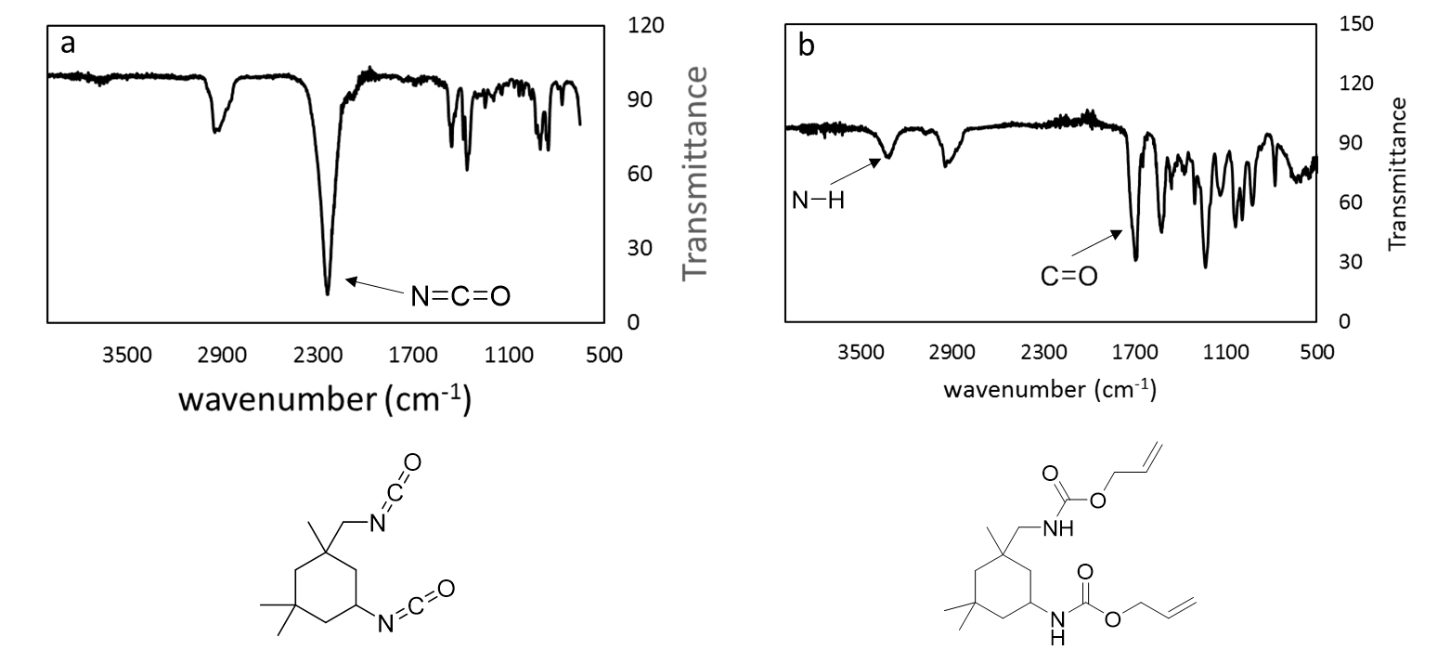


**Supplementary Figure 27**. FTIR spectrum of (a) isophorone diisocyanate and (b) isophorone di(allyl urethane) monomer.


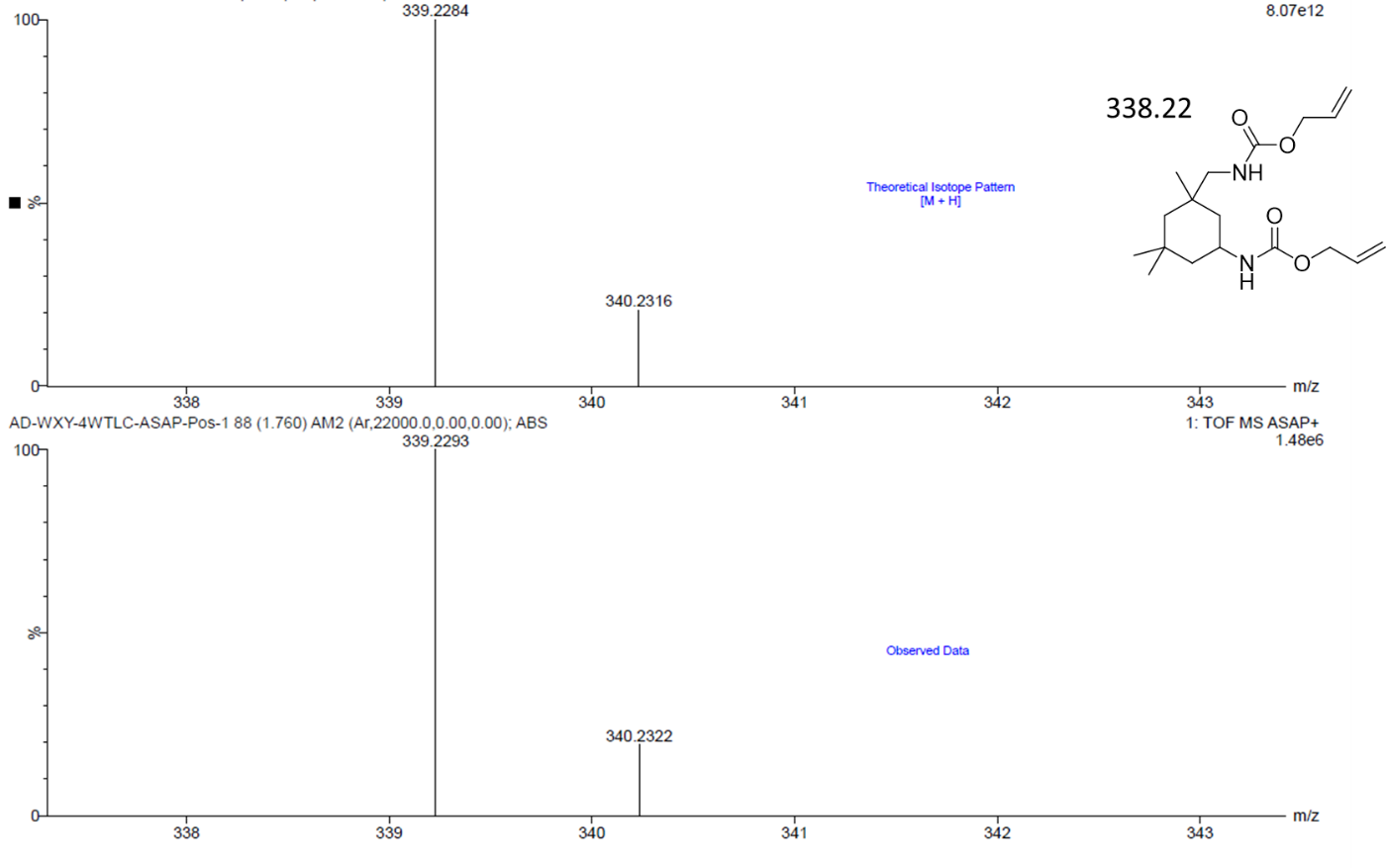


**Supplementary Figure 28**. Mass spectrum of isophorone di(allyl urethane) monomer (bottom) with theoretical isotope pattern (top).

**Supplementary Figure 29**. Representative scheme of crosslinking of resin upon exposure to light *via* radical thiol-ene addition.

**Polycarbonate Characterization**


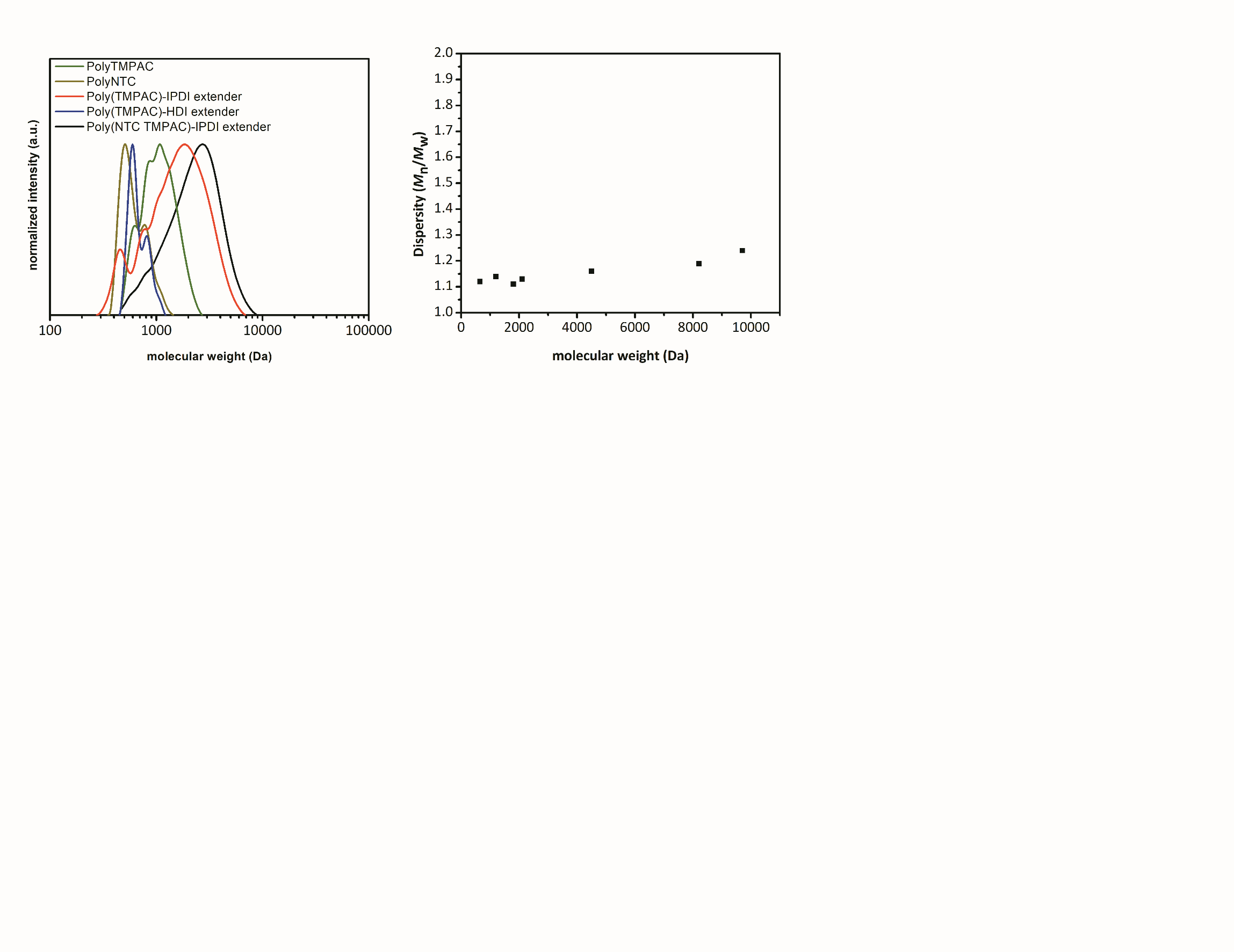


**Supplementary Figure 30**. Size exclusion chromatography (SEC) of polycarbonates of the number-average molecular weight *vs* dispersity for PolyTMPAC. *M*_n_ and *Đ*_M_ were determined in CHCl_3_ against poly(methyl methacrylate standards.


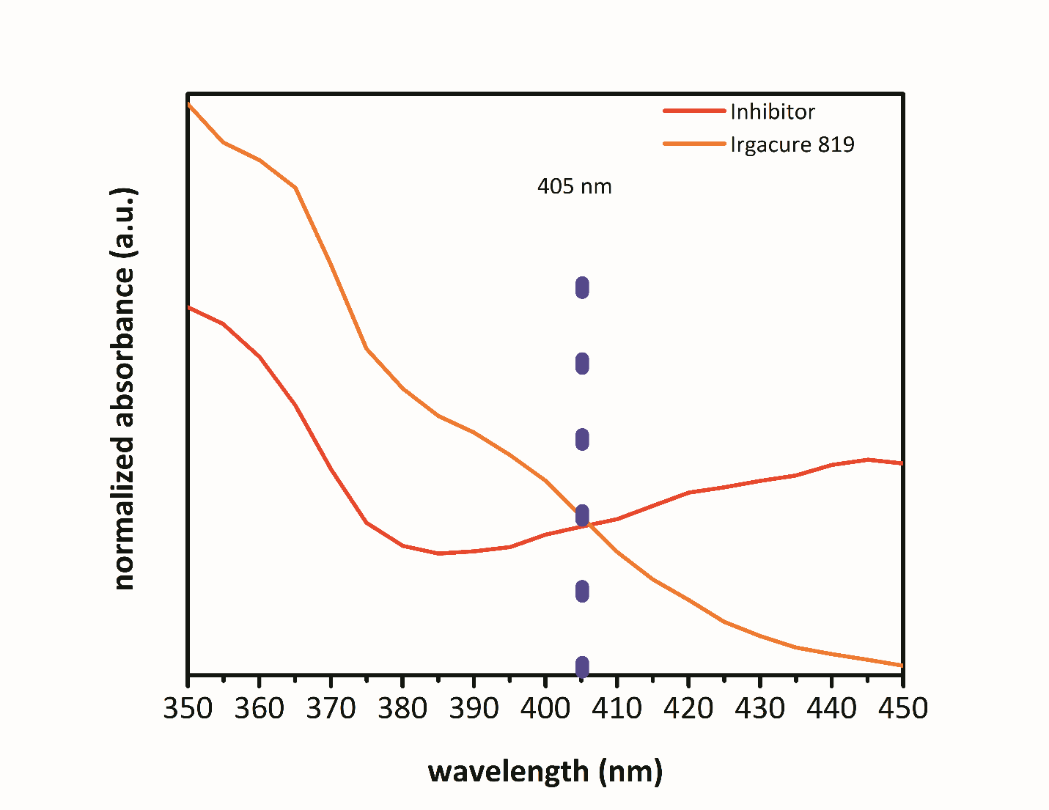


**Supplementary Figure 31**. Absorbance spectra of the photoinitiator and the photoinhibitors used in the polycarbonate resins, along with wavelength of interest (405 nm) of the printer marked.


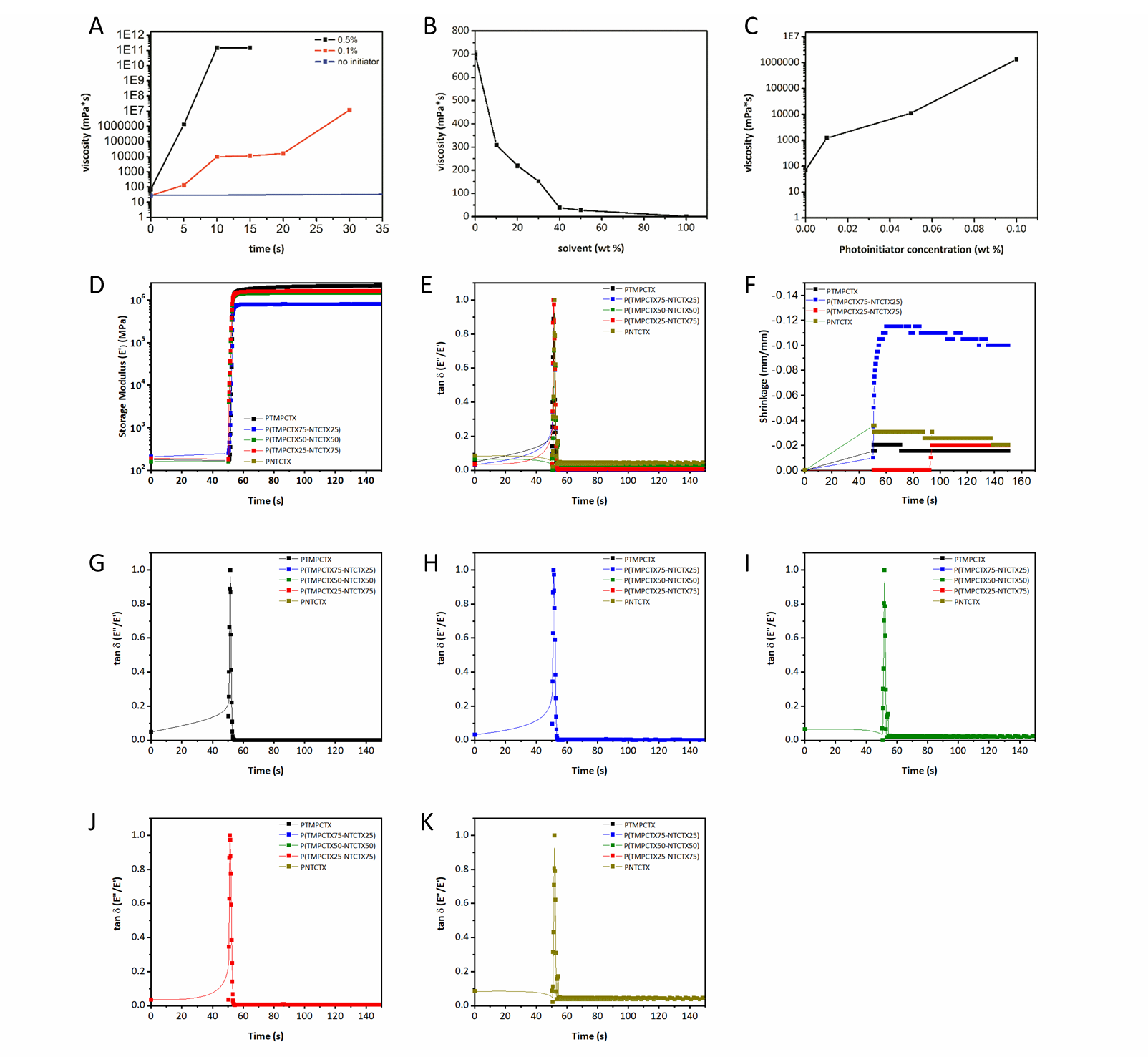


**Supplementary Figure 32**. The rate of crosslinking over time, viscosity vs photoinitiator concentration, and viscosity vs diluent concentration are plotted (A, B, C, respectively) for PTMPCTX resins. Photorheology of PCResinks with 1% wt photoinitiator and 1% photoinhibitor. Resin samples were sheared between two parallel plates, one made of glass and transparent, at 1 Hz for 50 s without irradiation at ambient conditions. After this time, the light source was switched on and measurements were taken every 0.2 s over the course of 2 min. Storage moduli (D) and loss factor (tan *δ*) (E) plots for resin compositions are displayed over time, accompanied by resin shrinkage over the course of film curing (F). Representative loss factor (tan *δ*) (E) plots for resin compositions are displayed over time (G-K) for PTMPCTX (G), P(TMPCTX75-NTCTX25) (H), P(TMPCTX50-NTCTX50) (I), P(TMPCTX25-NTCTX75) (J), P(NTCTX) (K).

**Printed Scaffold Morphological Characterization**


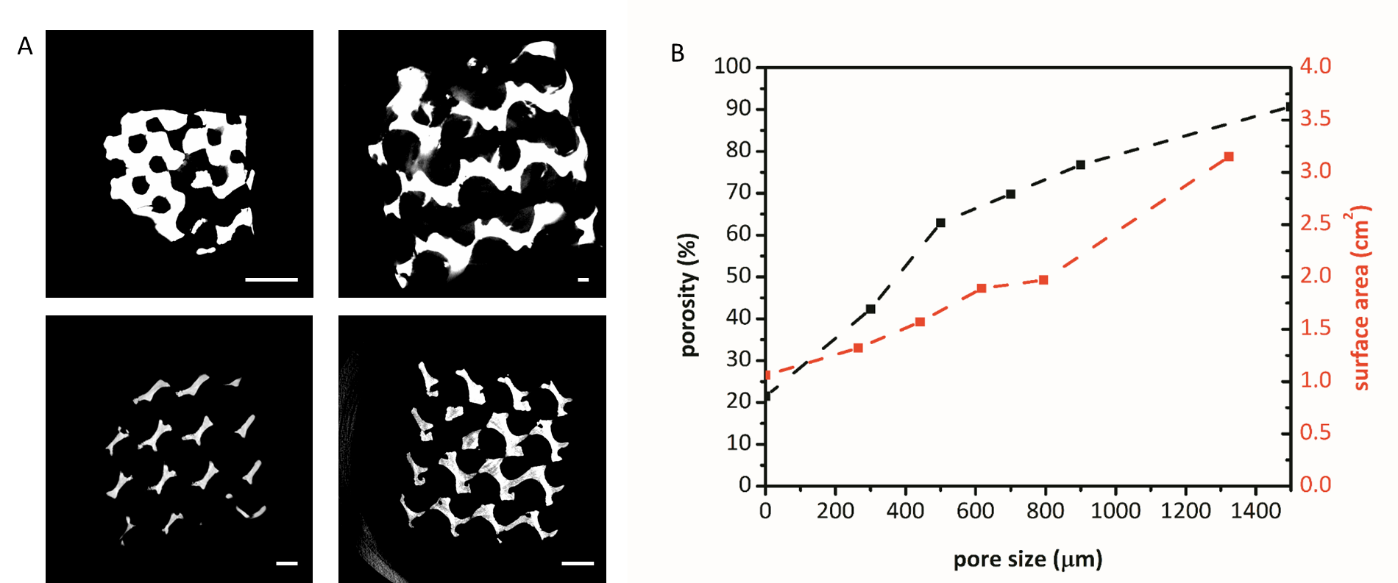


**Supplementary Figure 33**. (A) Representative CT scan cross-sections displaying different pore sizes for the printed scaffolds (scale bars = 1 mm). and (B) the relationship between the CAD design surface area and porosity with the printed part values. Images are representative of 5 formulations, 3 individual samples minimum per formulation, 200+ images per sample

**Cellular Proliferation and Imaging**


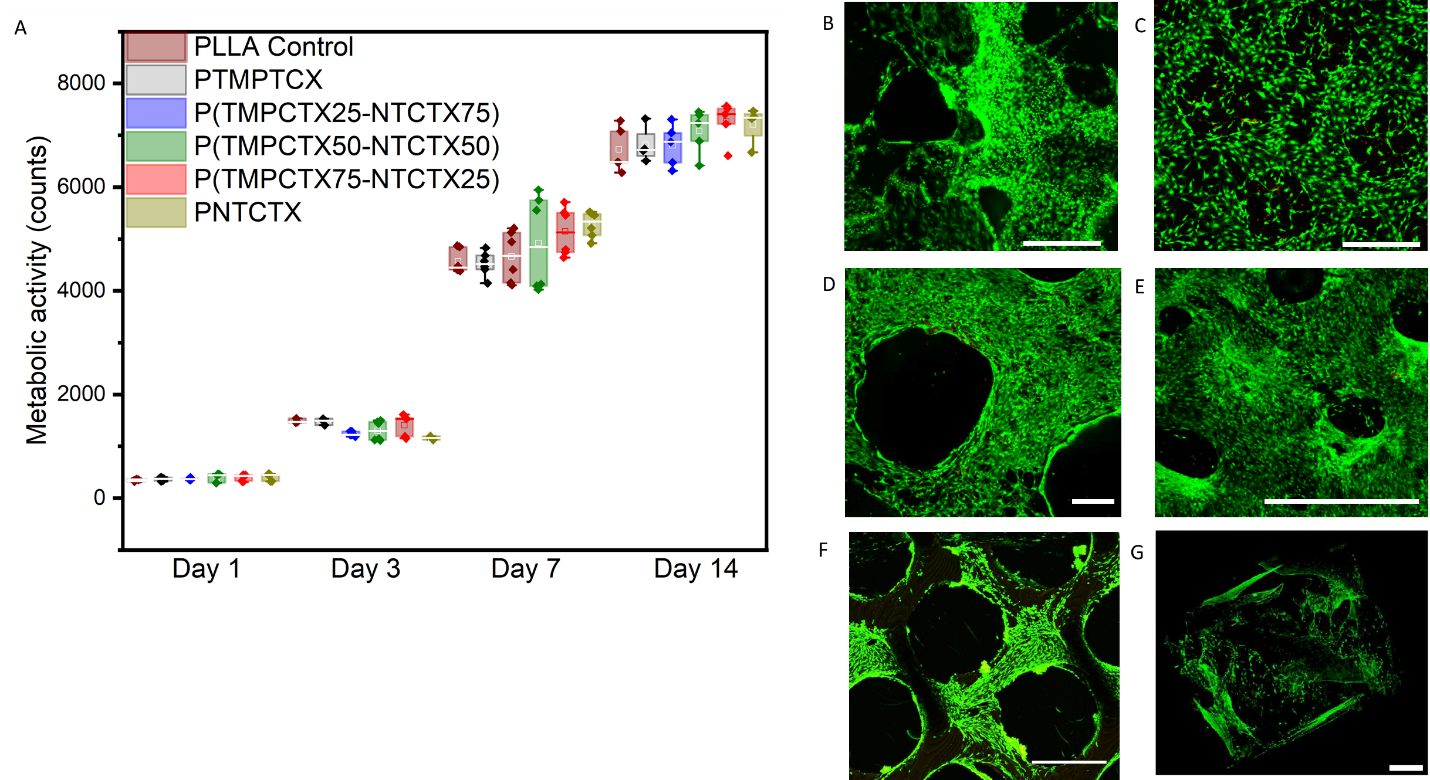


**Supplementary Figure 34**. Cellular proliferation (MC3T3 cell line) as determined from metabolic activity as measured by the Presto blue assay, noting the median along with the upper and lower quartile fluorescence values (box) with the maximum and minimum values displayed as bars, and the corresponding raw values as points within each plotted species. Experiments conducted with N=3, n = 4 (A), with corresponding representative images of 3D scaffolds at 24 h (B, C) and 7 days (D-F) to corresponding 1000 µm (B, D), 250 µm (C, E) and 700 µm (F) (Scale bars = 500 µm). A representative image of adipocytes seeded in solution with PTMPTCX materials (F, Scale bar = 10 µm), a reassembled 3D image of adipocyte proliferation throughout a scaffold (G, Scale bar = 100 µm).


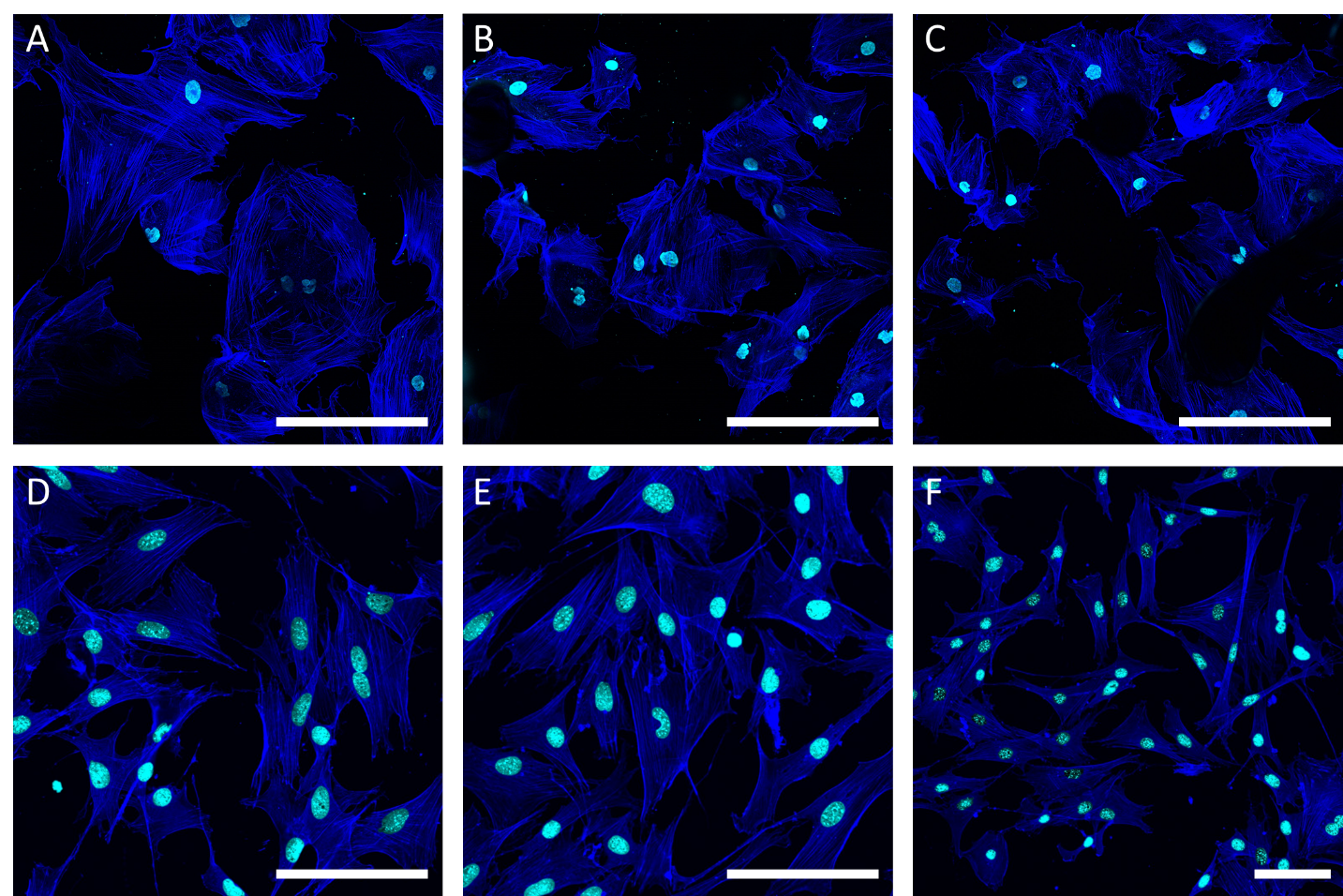


**Supplementary Figure 35**. Cellular spreading for fibroblasts (A-C) and adipocytes (D-F) on control samples (no polymer film, A & D), PTMPTCX film (B & E), and PNTCTX film (C & F ). (Scale bar = 10 µm)


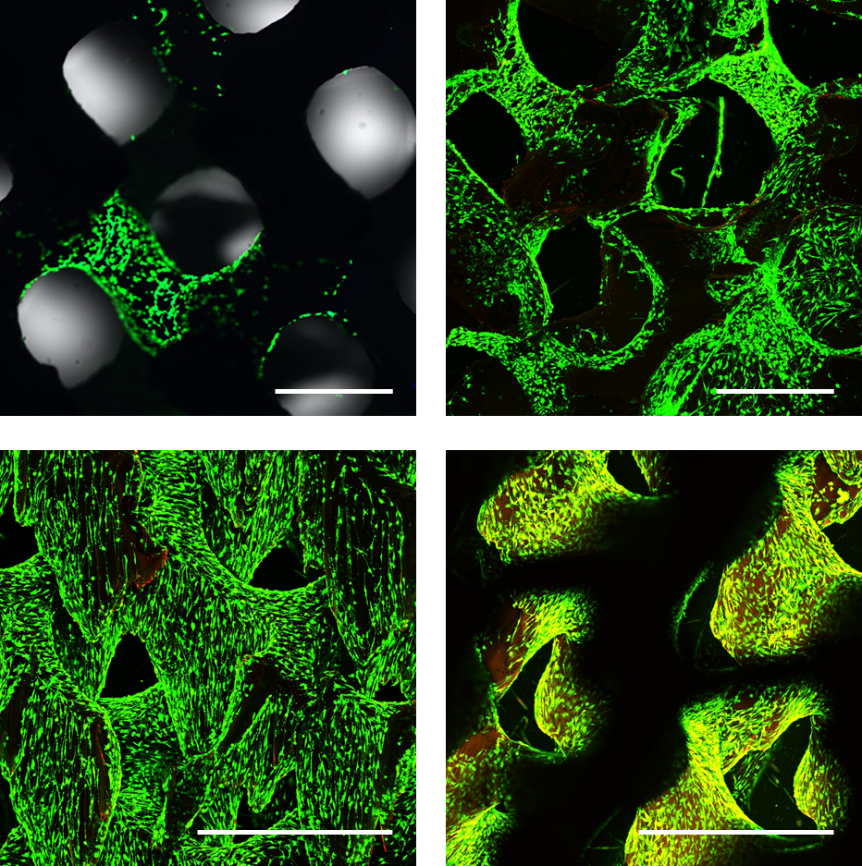


**Supplementary Figure 36**. Representative 3D images of NOR-10 cells proliferating along a PTMPTCX scaffold at 7 days, displaying different views of the scaffold. (Scale bar = 500 µm)

**Thermomechanical Characterization**


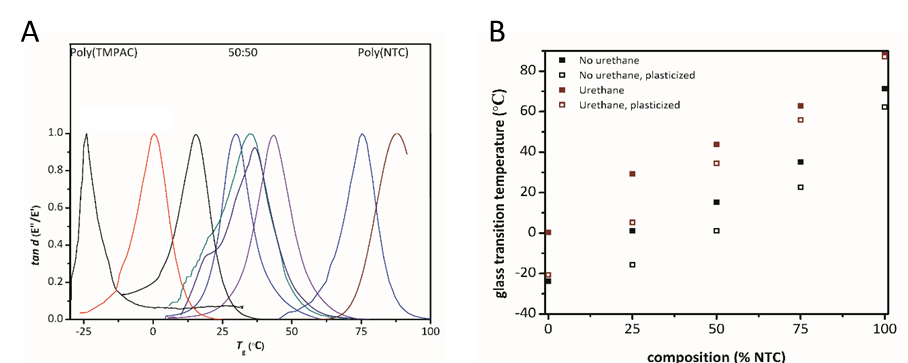


**Supplementary Figure 37**. Loss factor thermograms vs temperature and corresponding peak tan *δ* values as a function of NTC block composition for polycarbonate and poly(carbonate urethane) samples after submersion for 1 day in PBS. Samples were also assessed in PBS (A) and the shift in the *T*_g_ as a function of NTC composition and chain extension using IPDI in the polymer backbone (B). (n=5)

**
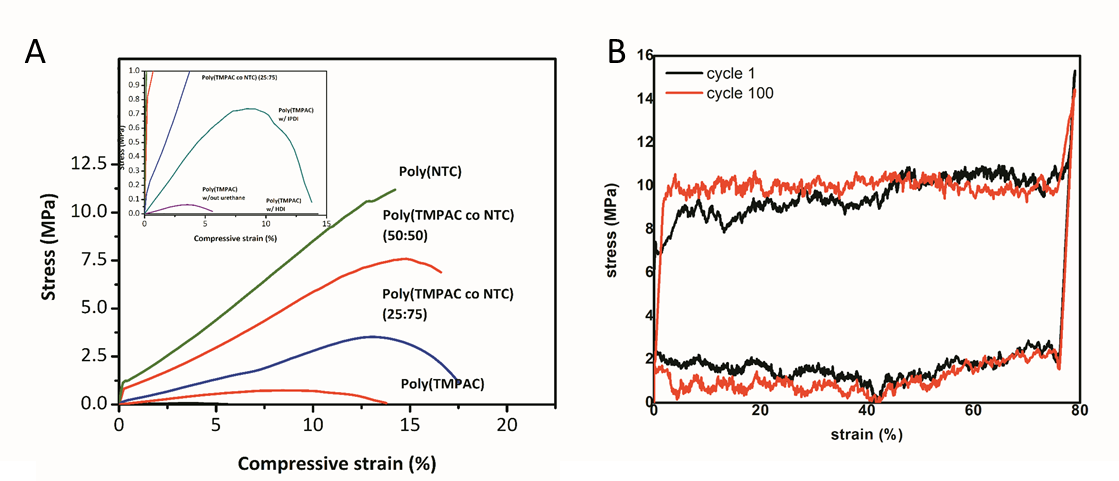
**

**Supplementary Figure 38**. (A) Compressive mechanical behaviors of scaffolds as measured by DMA compression (linear region only) and (B) the compressive behavior of the alginate-scaffold in PBS solution at 37 °C.

**
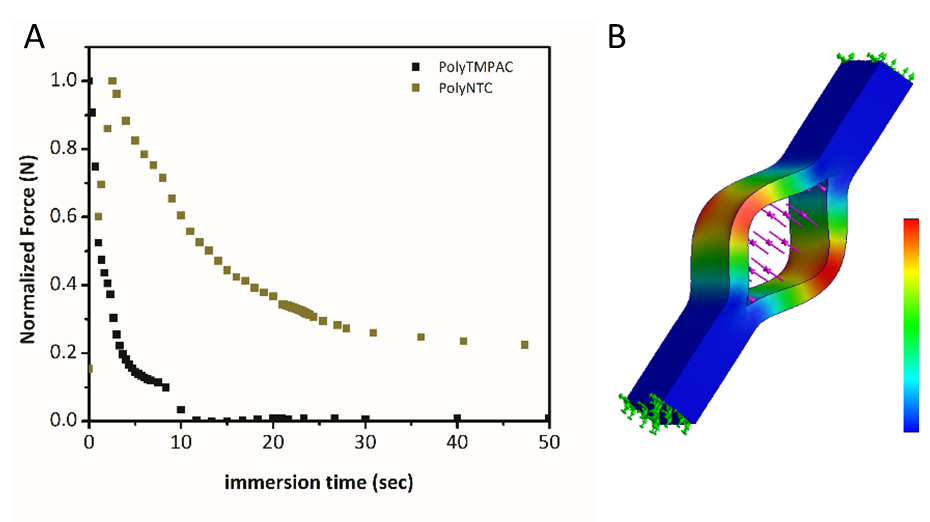
**

**Supplementary Figure 39**. Stress-strain recovery plots for compressed scaffolds immersed in 37 °C (A) with a corresponding FEA model of representative soft tissue void with the peak expansion force used to determine the deformation of the “soft tissue” (based on alginate mechanical properties) with an arbitrary gradient shown (B).

**Shape Memory Characterization**

**Supplementary Table 1.** Shape memory properties of the printed scaffolds.

| Composition | Strain fixation (*T*_g_-20°C), % | Strain fixation (*T*_g_), % | Strain recovery (*T*_g_ -20°C), % | Strain recovery (*T*_g_), % |
| --- | --- | --- | --- | --- |
| PTMPCTX | 100 | 0 | 51 | 100 |
| P(TMPCTX75-NTCTX25) | 100 | 83 | 0 | 100 |
| P(TMPCTX50-NTCTX50) | 100 | 97 | 0 | 100 |
| P(TMPCTX25-NTCTX75) | 100 | 100 | 0 | 100 |
| PNTCTX | 100 | 100 | 0 | 100 |


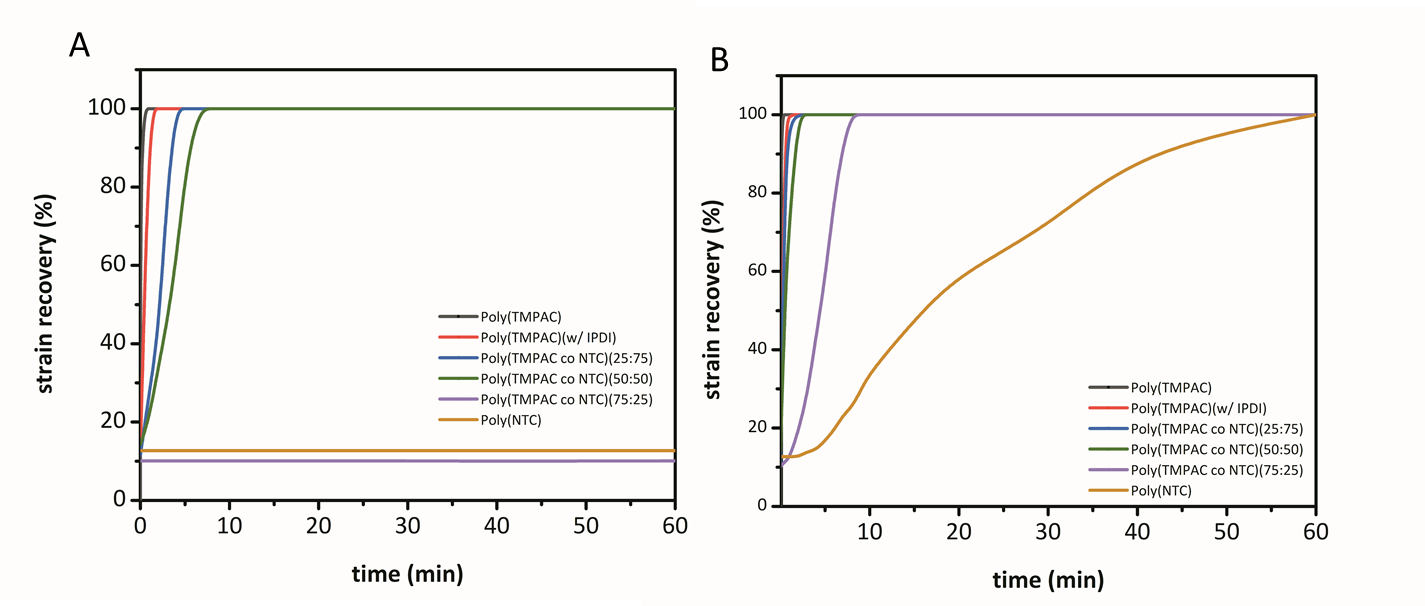


**Supplementary Figure 40**. Shape recovery behaviors of printed scaffolds as a function environmental conditions, where porous printed scaffolds were examined at 25 °C in ambient conditions (A) and at 37 °C in PBS (B).

**
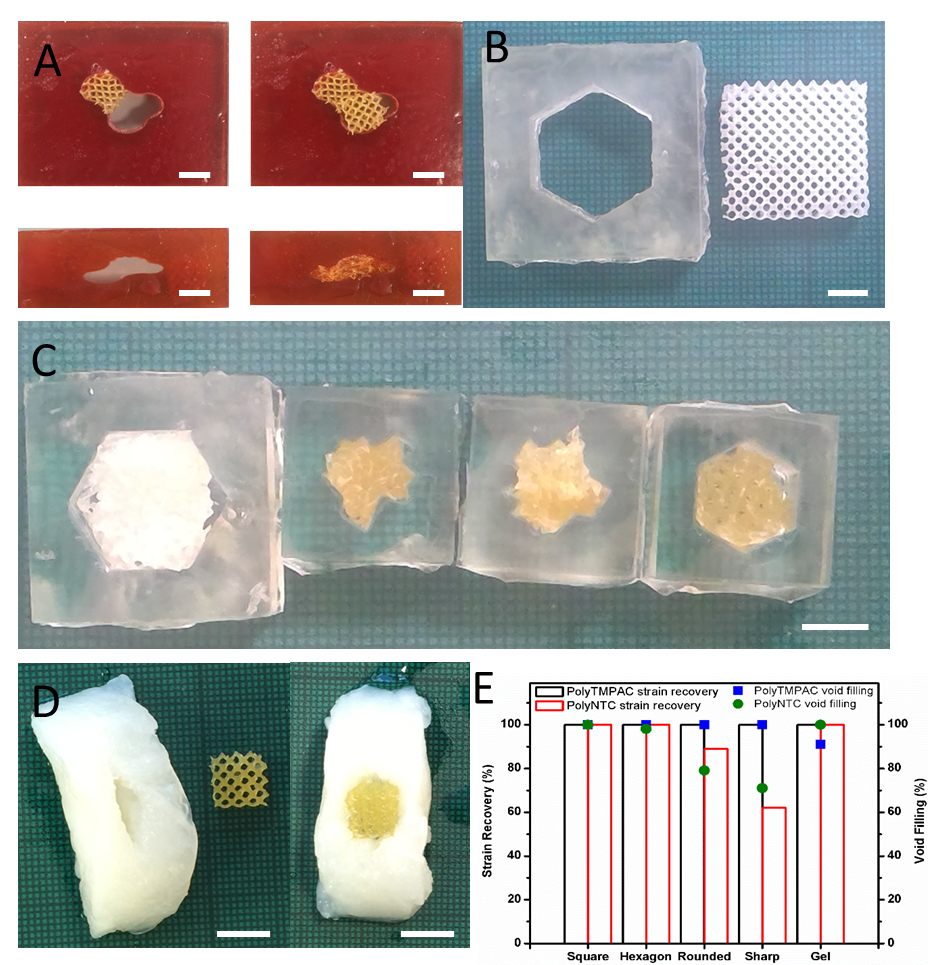
**

**Supplementary Figure 41**. Void filling of various irregular (A) and regular (B-C) hard and soft (D) voids, produced from 3D printed designs and alginate voids using mock subcutaneous openings. (E) Void filling was measured using cross sectional area after driving full recovery of the scaffold. (Scale bar = 1 cm)

**Gravimetric Analysis**


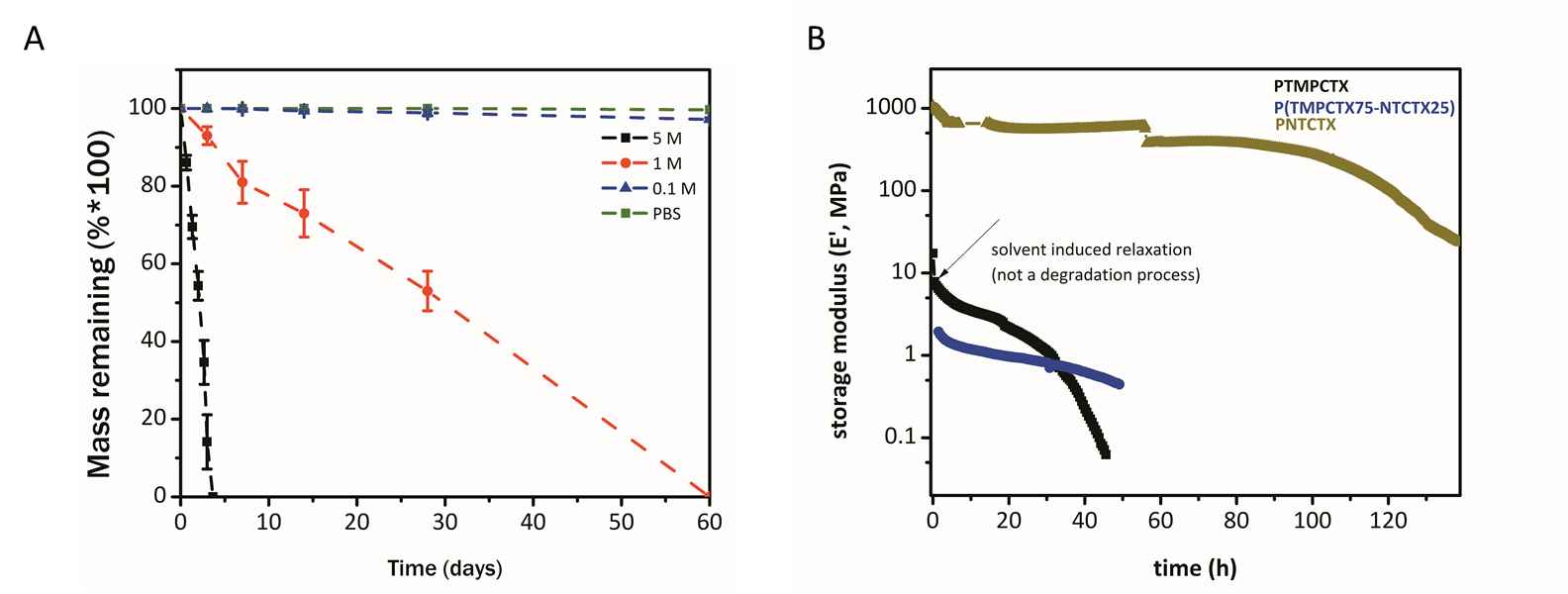


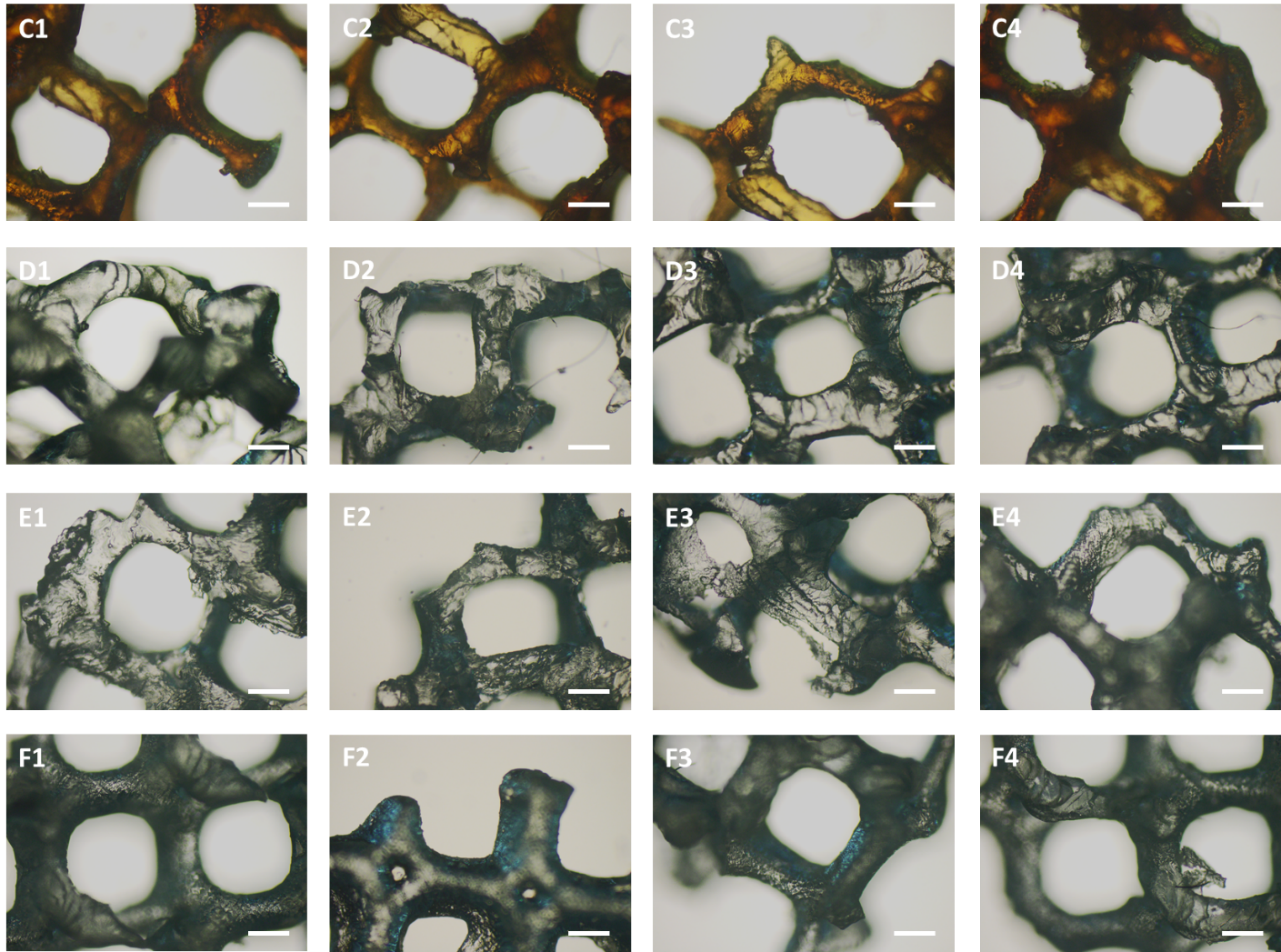


**Supplementary Figure 42**. Gravimetric degradation analysis in various hydrolytic solutions for PTMPCTX samples (A), and representative mechanical degradation profiles of films in 5 M NaOH, loaded at 1 Hz deformed 50 µm per cycle (B). (C-F) Recovered *in vivo* samples and virgin (C) material displaying surface erosion characteristics including erosion of layer lines from 1 month (D) to 2 (E) and 4 months (F). (A-B) Points are representative of mean degradation behavior, with standard deviations calculated from sample masses (n=6). For each time point (C-F), Images are representative of 2 formulations, 2 different physical forms, 3 individual samples minimum per formulation. (Scale bar = 300µm)

**Histology Analysis**


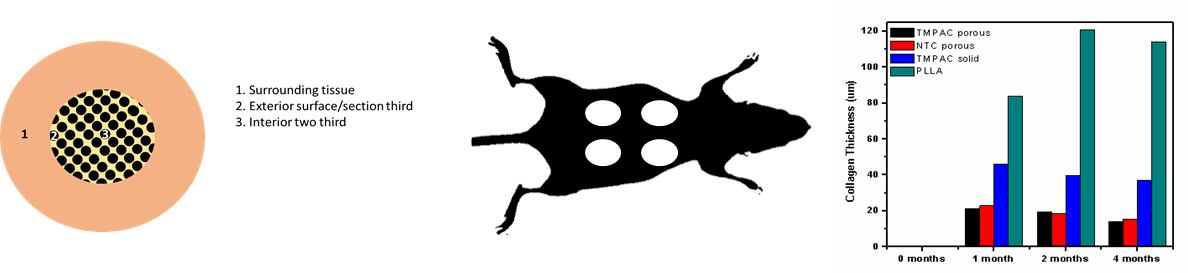


**Supplementary Figure 43**. Implant orientation for 4 months subcutaneous murine study, with section analysis nomenclature (exterior, interface, interior) labeled on a cartoon model of the porous implant.


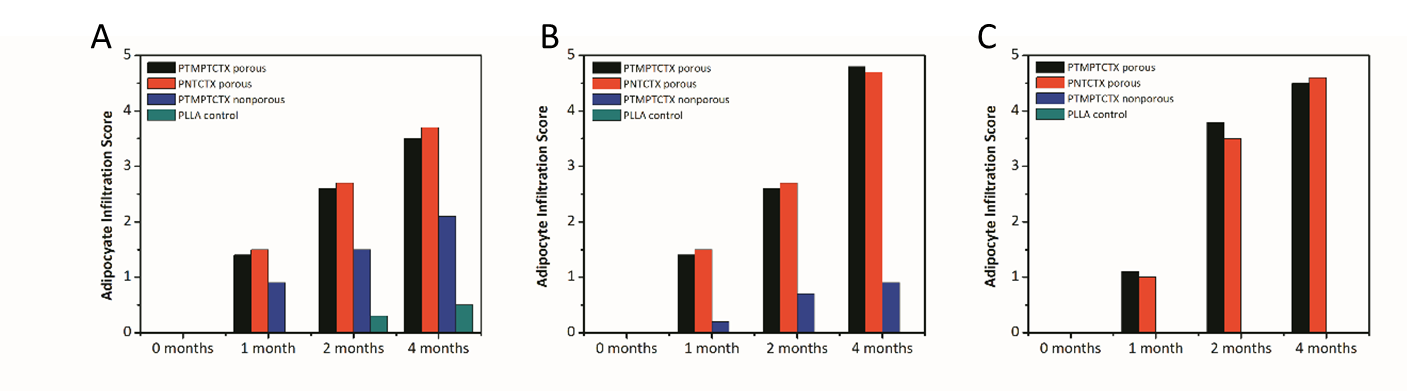


**Supplementary Figure 44**. Histological scoring of macrophage presence at the implant site, divided into the exterior of the tissue (A), at the interface of the implant and tissue (B), and within the implant (C).


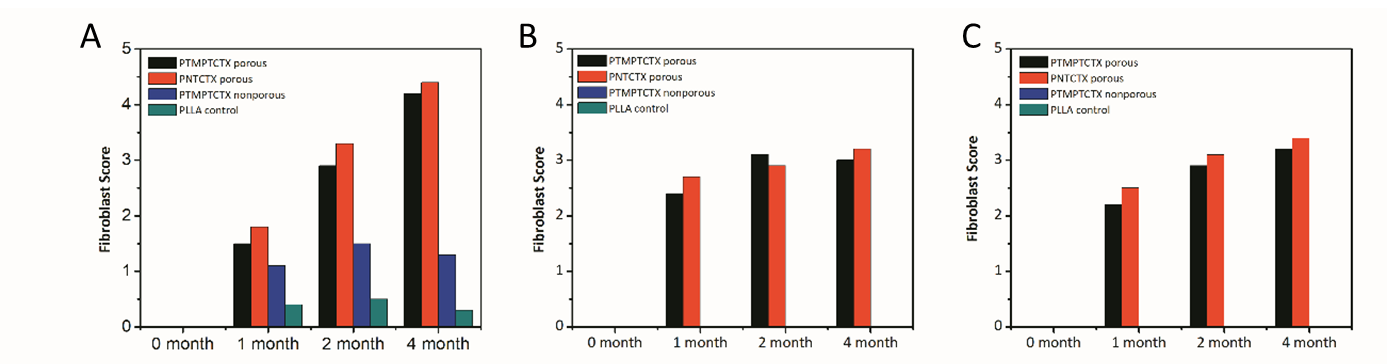


**Supplementary Figure 45**. Histological scoring of fibroblast presence at the implant site, divided into the exterior of the tissue (A), at the interface of the implant and tissue (B), and within the implant (C).


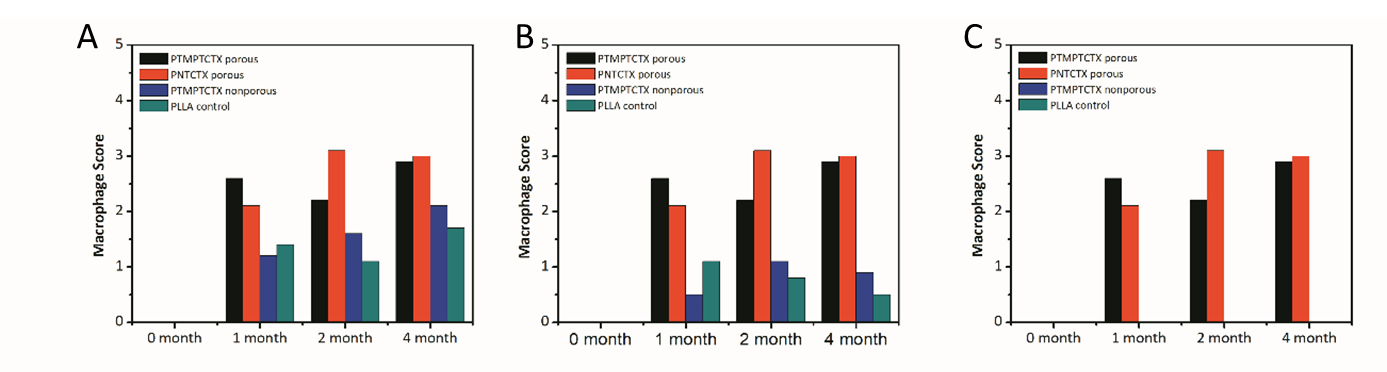


**Supplementary Figure 46.** Histological scoring of macrophage presence at the implant site, divided into the exterior of the tissue (A), at the interface of the implant and tissue (B), and within the implant (C).


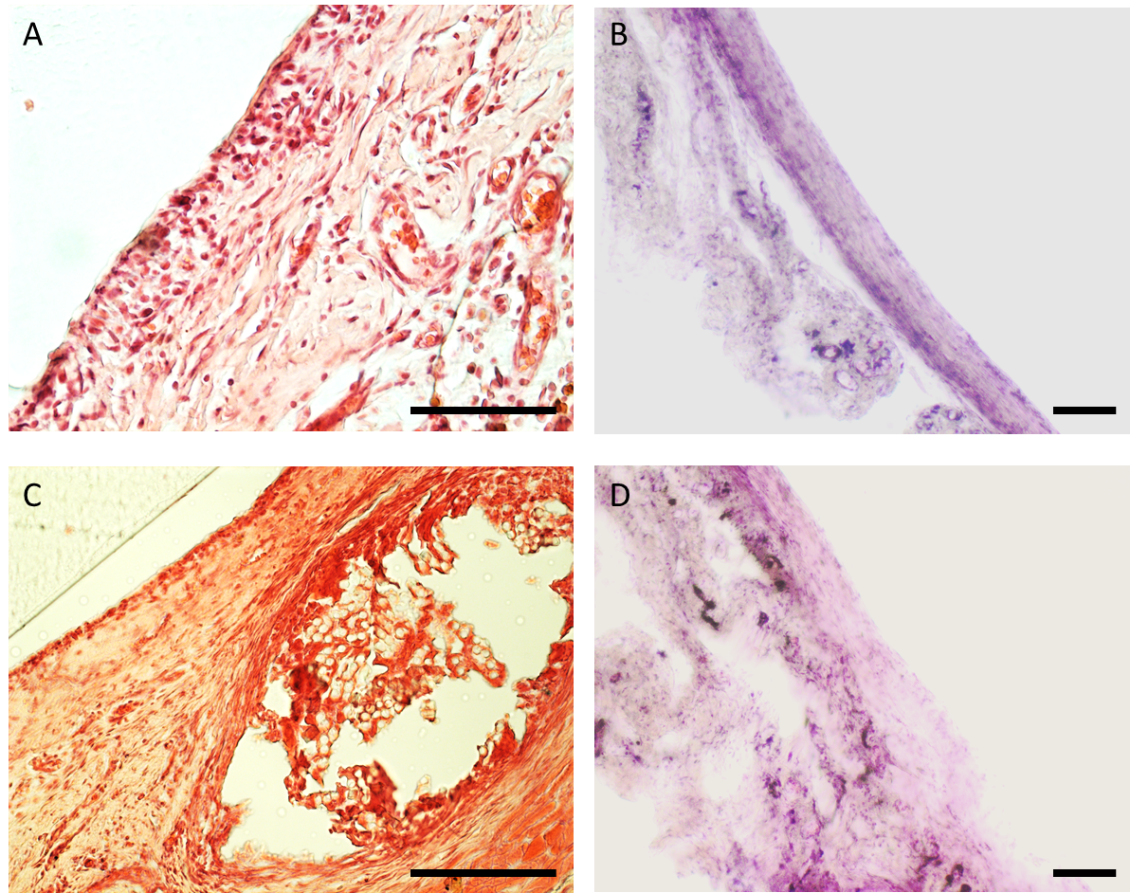


**Supplementary Figure 47**. Representative tissue cross sections of implanted PTMPCTX at 1 (A-B) and 4 (C-D) months stained with H&E (A,C) and Masson’s Trichrome (B,D). (Scale bar = 200 µm)


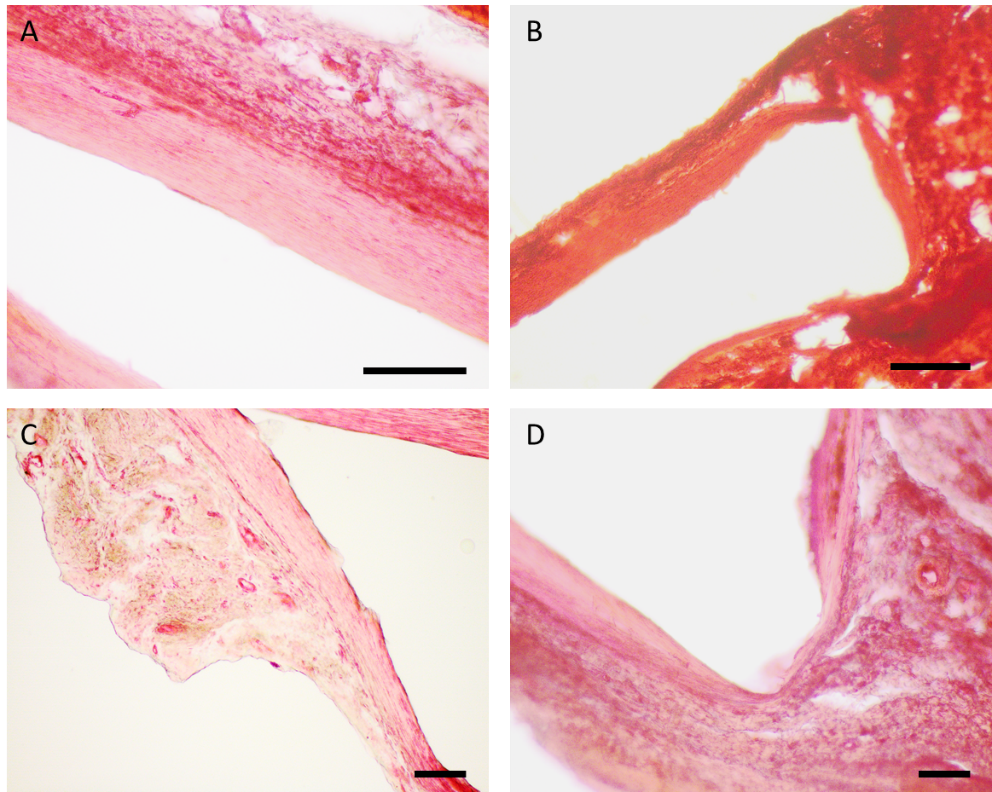


**Supplementary Figure 48**. Representative tissue cross sections of PLLA implant sites at 1 (A-B) and 4 (C-D) months, stained with H&E (A,C,D) and picrosirus red (B). (Scale bar = 200 µm)


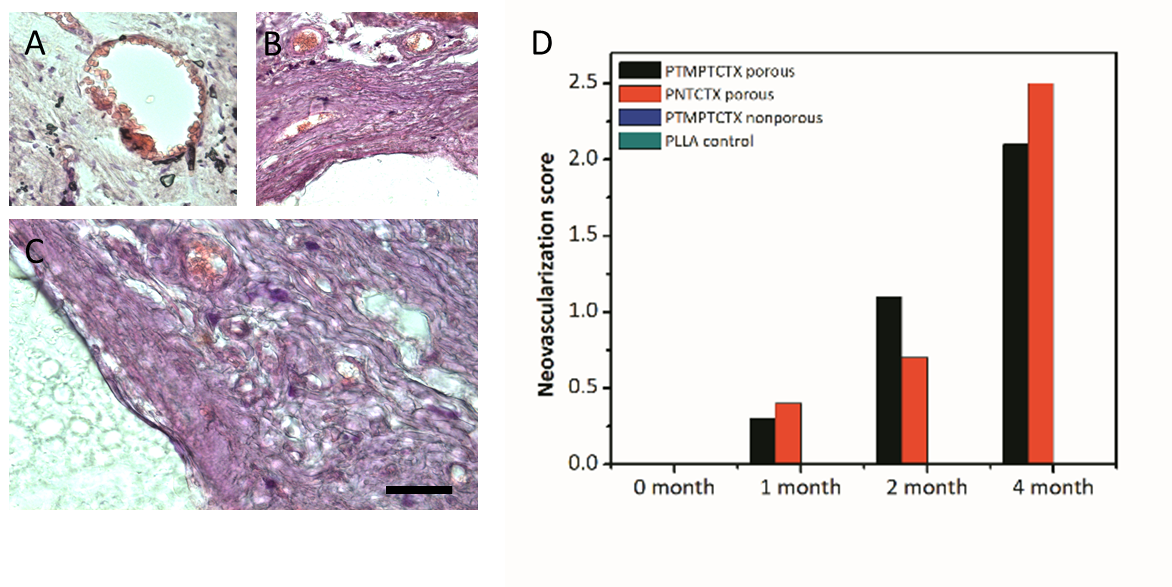


**Supplementary Figure 49**. Representative images of vascularization in the printed PTMPCTX (A), printed PNTCTX (B), and non-porous PTMPCTX (C) scaffolds at 2 months, and the corresponding neovascularization score (D). (Scale bar = 100 µm)


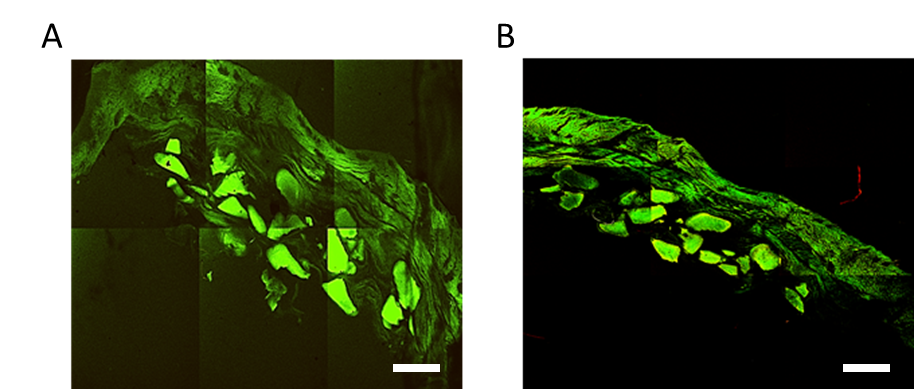


**Supplementary Figure 50**. Representative confocal images of H&E stained PolyNTC (a) and P PTMPCTX (b) scaffolds at 4 months, displaying no calcification areas. (Scale bar = 1 mm)
