# Supplementary material for: 4D polycarbonates via stereolithography as scaffolds for soft tissue repair
Source: Nat Commun. 2021 Jul 5;12:3771. doi: 10.1038/s41467-021-23956-6 (PMC8257657; doi:10.1038/s41467-021-23956-6)
Supplement: Supplementary file 2 — Description of Additional Supplementary Files [file 41467_2021_23956_MOESM2_ESM.docx]

**Description of Additional Supplementary Files**

**Supplementary Video 1:**

Cyclic compression (10 cycles) of PTMPCTX scaffold at ambient conditions

**Supplementary Video 2:**

Shape memory demonstrated of PTMPCTX scaffold expanding in alginate void mold

**Supplementary Video 3:**

Surface erosion of PTMPCTX scaffold pore in accelerated hydrolytic solution
