# Supplementary material for: 4D polycarbonates via stereolithography as scaffolds for soft tissue repair
Source: Nat Commun. 2021 Jul 5;12:3771. doi: 10.1038/s41467-021-23956-6 (PMC8257657; doi:10.1038/s41467-021-23956-6)
Supplement: Supplementary file 6 — Reporting Summary [file 41467_2021_23956_MOESM6_ESM.pdf]

nature research

Corresponding author(s): Andrew Dove

Last updated by author(s): 4th May 2021

Reporting Summary

Nature Research wishes to improve the reproducibility of the work that we publish. This form provides structure for consistency and transparency in reporting. For further information on Nature Research policies, see Authors & Referees and the Editorial Policy Checklist .

Statistics

For all statistical analyses, confirm that the following items are present in the figure legend, table legend, main text, or Methods section.

n/a	Confirmed	
☐	☒	The exact sample size (n) for each experimental group/condition, given as a discrete number and unit of measurement
☐	☒	A statement on whether measurements were taken from distinct samples or whether the same sample was measured repeatedly
☐	☒	The statistical test(s) used AND whether they are one- or two-sided<br>*Only common tests should be described solely by name; describe more complex techniques in the Methods section.*
☒	☐	A description of all covariates tested
☒	☐	A description of any assumptions or corrections, such as tests of normality and adjustment for multiple comparisons
☐	☒	A full description of the statistical parameters including central tendency (e.g. means) or other basic estimates (e.g. regression coefficient) AND variation (e.g. standard deviation) or associated estimates of uncertainty (e.g. confidence intervals)
☐	☒	For null hypothesis testing, the test statistic (e.g. F, t, r) with confidence intervals, effect sizes, degrees of freedom and P value noted<br>*Give P values as exact values whenever suitable.*
☒	☐	For Bayesian analysis, information on the choice of priors and Markov chain Monte Carlo settings
☒	☐	For hierarchical and complex designs, identification of the appropriate level for tests and full reporting of outcomes
☒	☐	Estimates of effect sizes (e.g. Cohen's d, Pearson's r), indicating how they were calculated

Our web collection on statistics for biologists contains articles on many of the points above.

Software and code

Policy information about availability of computer code

Data collection	RheoCompass ™ software (v1.20.496) was used for rheology on a Anton Paar Modular Compact Rheometer MCR302; STARe software (v10.00) was used for thermomechanical measurements on a STARe System DMA 1, Mettler Toledo; Solidworks software was used for stl file generation; SkyScan software (NRecon v1.6.2) was used to reconstruct microCT images into 3D structures. Sample processing was conducted using Origin v8 software (processing and figure generation)
Data analysis	Statistical analysis has been performed on the thermomechanical analysis (DMA, tensile testing), rheology, gravimetric analysis, histopathological scoring and on the cell viability data using a two-way ANOVA test or a Welch's unpaired t test, with p < 0.05, analysed with GraphPrism 8 software.

For manuscripts utilizing custom algorithms or software that are central to the research but not yet described in published literature, software must be made available to editors/reviewers. We strongly encourage code deposition in a community repository (e.g. GitHub). See the Nature Research guidelines for submitting code & software for further information.

Data

Policy information about availability of data

All manuscripts must include a data availability statement. This statement should provide the following information, where applicable:

- Accession codes, unique identifiers, or web links for publicly available datasets
- A list of figures that have associated raw data
- A description of any restrictions on data availability

The authors declare that the data supporting the findings of this study are available within the paper and its supplementary information file. Raw data underlying the figures presented are available from the corresponding authors upon reasonable request.

Field-specific reporting

Please select the one below that is the best fit for your research. If you are not sure, read the appropriate sections before making your selection.

- [x] Life sciences
- [ ] Behavioural & social sciences
- [ ] Ecological, evolutionary & environmental sciences

For a reference copy of the document with all sections, see nature.com/documents/nr-reporting-summary-flat.pdf

Life sciences study design

All studies must disclose on these points even when the disclosure is negative.

Sample size	6 replications were used for each material at each time point (sample power greater than 80%, degrees of freedom above 10, 5% uncertainty)
Data exclusions	No data was excluded from analysis.
Replication	For cell studies: three independent experiments were performed for each sample. In each independent experiment, samples were replicated at least three times. All attempts at replication were successful.<br>For animal studies: 6 replications were used for each material at each time point. All replications were successful.
Randomization	Cells and animals were randomly allocated into experimental groups.
Blinding	Investigators were blinded during data analysis for both cell studies and animal studies.

Reporting for specific materials, systems and methods

We require information from authors about some types of materials, experimental systems and methods used in many studies. Here, indicate whether each material, system or method listed is relevant to your study. If you are not sure if a list item applies to your research, read the appropriate section before selecting a response.

Materials & experimental systems

n/a	Involved in the study	
☐	☒	Antibodies
☐	☒	Eukaryotic cell lines
☒	☐	Palaeontology
☐	☒	Animals and other organisms
☒	☐	Human research participants
☒	☐	Clinical data

Methods

n/a	Involved in the study	
☒	☐	ChIP-seq
☒	☐	Flow cytometry
☒	☐	MRI-based neuroimaging

Antibodies

Antibodies used	Mouse primary anti-Vinculin antibody [VIN-54] (ab130007) from Abcam and Donkey anti-Mouse IgG (H+L) Highly Cross-Adsorbed Secondary Antibody, Alexa Fluor Plus 594 from Invitrogen.
Validation	Tested applications, suitable for: ICC/IF, WB, IHC-Fr. Species reactivity, reacts with: Mouse, Rat, Chicken, Human, Monkey

Eukaryotic cell lines

Policy information about cell lines

Cell line source(s)	Fibroblasts (human and murine), adipocytes, and macrophages (NOR-10 (murine fibroblasts), Hs 792 (human fibroblasts), IC21 (murine macrophages), and D16 (murine adipocytes)) were purchased from the American Type Culture Collection (ATCC). MC3T3 (murine pre-osteoblasts) were purchased from Public Health England.
Authentication	None of the cell lines were authenticated.
Mycoplasma contamination	None of the cell lines were tested for mycoplasma infection.
Commonly misidentified lines<br>(See ICLAC register)	No commonly misidentified cell lines were used in this study.

Animals and other organisms

Policy information about studies involving animals; ARRIVE guidelines recommended for reporting animal research

Laboratory animals	Adult (8 weeks old) male Sprague Dawley rats (200-300 g).
Wild animals	Did not involve wild animals
Field-collected samples	Did not involve field-collected samples
Ethics oversight	European Commission Directive 2010/63/EU (European Convention for the Protection of Vertebrate Animals used for Experimental and Other Scientific Purposes) and the United Kingdom Home Office (Scientific Procedures) Act (1986) with project approval from the institutional animal welfare and ethical review body (AWERB).

Note that full information on the approval of the study protocol must also be provided in the manuscript.
